# 2-Acetamido-2-deoxy-d-glucono-1,5-lactone Sulfonylhydrazones: Synthesis and Evaluation as Inhibitors of Human OGA and HexB Enzymes

**DOI:** 10.3390/ijms23031037

**Published:** 2022-01-18

**Authors:** Mariann Kiss, István Timári, Teréz Barna, Zuzana Mészáros, Kristýna Slámová, Pavla Bojarová, Vladimír Křen, Joseph M. Hayes, László Somsák

**Affiliations:** 1 Department of Organic Chemistry, University of Debrecen, POB 400, H-4002 Debrecen, Hungary; kiss.mariann@science.unideb.hu (M.K.); timari.istvan@science.unideb.hu (I.T.); 2 Department of Genetics and Applied Microbiology, University of Debrecen, POB 400, H-4002 Debrecen, Hungary; barna.terez@science.unideb.hu; 3 Laboratory of Biotransformation, Institute of Microbiology of the Czech Academy of Sciences, Vídeňská 1083, CZ-14220 Praha 4, Czech Republic; zuzana.meszaros@biomed.cas.cz (Z.M.); slamova@biomed.cas.cz (K.S.); bojarova@biomed.cas.cz (P.B.); kren@biomed.cas.cz (V.K.); 4 Faculty of Food and Biochemical Technology, University of Chemistry and Technology Prague, Technická 1903/3, CZ-16628 Praha 6, Czech Republic; 5 School of Pharmacy & Biomedical Sciences, University of Central Lancashire, Preston PR1 2HE, UK; JHayes@uclan.ac.uk

**Keywords:** hOGA, hHexB, inhibitor, glyconolactone sulfonylhydrazone, Prime refinement, QM/MM optimization

## Abstract

Inhibition of the human *O*-linked β-*N*-acetylglucosaminidase (hOGA, GH84) enzyme is pharmacologically relevant in several diseases such as neurodegenerative and cardiovascular disorders, type 2 diabetes, and cancer. Human lysosomal hexosaminidases (hHexA and hHexB, GH20) are mechanistically related enzymes; therefore, selective inhibition of these enzymes is crucial in terms of potential applications. In order to extend the structure–activity relationships of OGA inhibitors, a series of 2-acetamido-2-deoxy-d-glucono-1,5-lactone sulfonylhydrazones was prepared from d-glucosamine. The synthetic sequence involved condensation of *N*-acetyl-3,4,6-tri-*O*-acetyl-d-glucosamine with arenesulfonylhydrazines, followed by MnO_2_ oxidation to the corresponding glucono-1,5-lactone sulfonylhydrazones. Removal of the *O*-acetyl protecting groups by NH_3_/MeOH furnished the test compounds. Evaluation of these compounds by enzyme kinetic methods against hOGA and hHexB revealed potent nanomolar competitive inhibition of both enzymes, with no significant selectivity towards either. The most efficient inhibitor of hOGA was 2-acetamido-2-deoxy-d-glucono-1,5-lactone 1-naphthalenesulfonylhydrazone (**5f**, *K*_i_ = 27 nM). This compound had a *K*_i_ of 6.8 nM towards hHexB. To assess the binding mode of these inhibitors to hOGA, computational studies (Prime protein–ligand refinement and QM/MM optimizations) were performed, which suggested the binding preference of the glucono-1,5-lactone sulfonylhydrazones in an *s-cis* conformation for all test compounds.

## 1. Introduction

*O*-GlcNAcylation is a dynamic posttranslational modification in which a single β-*N*-acetylglucosamine (GlcNAc) moiety is added to or removed from the serine/threonine residues of nucleocytosolic and mitochondrial proteins. The dynamic cycling of *O*-GlcNAc modification on numerous, functionally distinct proteins is catalyzed by two enzymes: *O*-linked β-*N*-acetylglucosaminyltransferase (OGT, EC 2.4.1.255; GT41) and β-*N*-acetylglucosaminidase (OGA, EC 3.2.1.169; GH84) in response to cellular signals or cellular phases, similar to phosphorylation [1]. Indeed, in many cases, phosphorylation and *O*-GlcNAcylation target the same serine/threonine residues. Misregulation of the *O*-GlcNAc cycle can influence numerous cell signaling processes and contribute to the development of various diseases such as neurodegenerative and cardiovascular disorders, type 2 diabetes, and cancer [2]. Changes in the *O*-GlcNAcylation status of proteins can lead to alterations in protein folding, cellular localization, and catalytic activity [3], all of which can actively influence downstream biological processes associated with the protein in question and play a critical role in the development and progression of chronic diseases. Numerous papers [4,5,6,7] demonstrated that inhibition of OGA reduces the amount of the pathological tau protein in the brain, most likely by increasing its *O*-GlcNAcylation. Thus, by decreasing its phosphorylation, tau remains in the soluble, non-toxic form with reduced aggregation. Therefore, the development of OGA inhibitors has considerable significance in the design of new potential treatments for Alzheimer’s disease and other neurodegenerative tauopathies, which belong to the most serious health and socio-economic challenges of modern society.

PUGNAc (Table 1, **I**) was one of the first inhibitors of OGA [8]; however, it is not selective, since it also inhibits lysosomal hexosaminidases (HexA and HexB, EC 3.2.1.52; GH20) that cleave GlcNAc and GalNAc residues from oligosaccharides and glycosphingolipids. Neither the activity nor the selectivity of PUGNAc was significantly altered by modifications of the aromatic moiety [9]. OGA is active at neutral pH and is specific for the GlcNAc substrate, while hexosaminidases act at acidic (lysosomal) pH and show an affinity also for GalNAc. Both OGA and HexA/HexB utilize the substrate-assisted catalytic mechanism, in which the 2-acetamido group of the substrate forms an oxazoline intermediate [10], although they do not share sequence similarity. The synthesis of selective inhibitors can help avoid side effects by simultaneous inhibition of both enzymes. Any non-specific inhibition of hexosaminidases may lead to lysosomal ganglioside accumulation, resulting in the neurodegenerative Tay-Sachs and Sandhoff diseases [11].

In our previous work [12], 2-acetamido-2-deoxy-d-glucono-1,5-lactone 4-arylsemicarbazones (Table 1, **II**) were found to be nanomolar inhibitors of both hOGA and HexA/HexB enzymes. Nevertheless, these inhibitors showed only moderate selectivity (*K*_i(Hex)_/*K*_i(OGA)_ = 1–2). This was the first study in which modifications were made to the urethane linking moiety between the glycone and the aromatic residue related to PUGNAc. In the present work, our aim was to make a structure–affinity relationship study of the said linker to determine how respective modifications affect the inhibition of OGA and the selectivity towards HexA/HexB. For this purpose, a library of 2-acetamido-2-deoxy-d-glucono-1,5-lactone arenesulfonylhydrazones (Table 1, target compounds) was prepared and studied as inhibitors of the above enzymes using kinetic and computational methods.

## 2. Results and Discussion

### 2.1. Synthesis of Inhibitors

Although some glycono-1,5-lactone tosylhydrazones are known in the literature [13,14,15], no analogous derivatives of 2-acetamido-2-deoxy-d-glucono-1,5-lactone could be found. Therefore, the synthesis of the planned inhibitors (Table 2) was based on our previous work [12] encompassing the synthesis of 2-acetamido-2-deoxy-d-glucono-1,5-lactone semicarbazones. The starting material 2-acetamido-3,4,6-tri-*O*-acetyl-2-deoxy-d-glucopyranose (**1**) was obtained from d-glucosamine hydrochloride by a well-known two-step procedure [16,17] in 83% overall yield. Compound **1** was then converted by arenesulfonyl hydrazides **2** (either commercial or obtained from the corresponding arenesulfonyl chloride as described in the literature [18]) in the presence of 10 mol% *p*-toluenesulfonic acid catalyst in chloroform to afford *N*-glycosyl arenesulfonyl hydrazides **3**. The oxidation reactions to yield lactone hydrazones **4** were carried out with activated MnO_2_ in dichloromethane at reflux temperature. *O*-Deprotection of **4** was performed with a solution of NH_3_ in MeOH to result in test compounds **5**. The overall yields of these five-step synthetic sequences affording inhibitors **5** from d-glucosamine were in the range of 26–44%.

**Figure 1:**
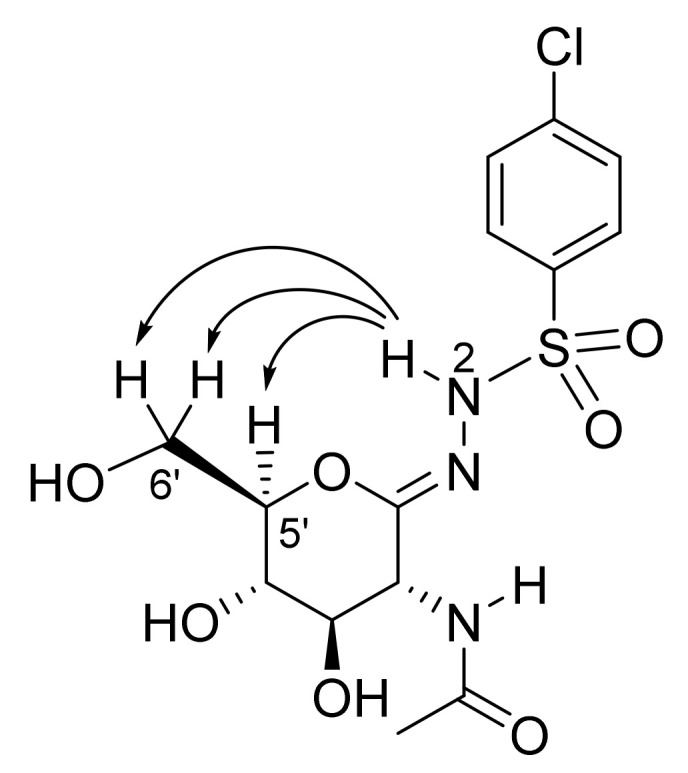
Nuclear Overhauser effects (NOEs) observed in compound **5d**.

To determine the configuration of the C=N bond in compounds **5**, a nuclear Overhauser effect (NOE)-based NMR approach was applied to **5d**. A 2D ^1^H-^1^H EASY ROESY experiment [19] was performed, which is a robust variant of NOE methods for small- and medium-sized molecules. The maximum spatial distance that can provide an NOE signal is about 5 Å. Thus, the *E-Z* isomeric forms of compound **5d** are expected to be distinguished based on informative NOE cross-peaks between the N(2)H and characteristic protons of the carbohydrate unit. In the ^1^H-^1^H ROESY spectrum (Figure S1) obtained for compound **5d**, NOE cross-peaks between the N(2)H resonance and H(5′), H(6′a,b) resonances can be observed (Figure 1), which indicate the vicinity in space of these protons and so the existence of the *Z* isomeric form. Since both the ^1^H and ^13^C NMR spectra for the other compounds **5** closely resemble those of **5d**, one can safely conclude the presence of the *Z* isomer also in those derivatives.

### 2.2. Inhibition of hOGA and hHexB by 2-Acetamido-2-deoxy-d-glucono-1,5-lactone Arenesulfonylhydrazones

The inhibitory potency of compounds **5a**–**f** was evaluated against recombinant hOGA and hHexB enzymes. The enzymes were prepared and purified as described previously [12,20]. For inhibition of hOGA, fluorescent 4-MU-GlcNAc was used as the substrate. Inhibition constants (*K*_i_) were determined by linear regression of data from Dixon plots using the competitive model as this mode of inhibition was proved by the Cornish–Bowden plots. In the case of inhibition of hHexB, Michaelis–Menten kinetics were evaluated using *p*NP-GlcNAc as a colorimetric substrate. Inhibition constants (*K*_i_) were determined by nonlinear regression analyses by employing the competitive inhibition model (cf. the Experimental section and Supplementary Materials).

The inhibition constants (*K*_i_) for most inhibitors **5** against hOGA were in the low nanomolar range (Table 3). The most potent inhibitors of hOGA were sulfonylhydrazones **5e** and **5f**, while **5b** proved to be the weakest one. On the other hand, lower *K*_i_ values against hHexB were obtained for all but one of the compounds, indicating that compounds **5** are better inhibitors of hHexB than of hOGA. Sulfonylhydrazone **5f** is the strongest inhibitor of both enzymes and the most selective towards hHexB after **5b** (**5b** is also the least potent inhibitor for both enzymes). Analogously to the parent structure PUGNAc, the newly prepared derivatives **5a**–**f** suffer from virtually no selectivity towards any of the tested enzymes, which suggests that the affinity of the aglycone part of the inhibitors might be similar for both glycosidases.

### 2.3. Computationally Predicted Enzyme–Inhibitor Binding in hOGA

To predict and analyze the hOGA binding modes of inhibitors **5**, Prime protein–ligand refinements [21] were performed based on their successful application to the previously reported 2-acetamido-2-deoxy-d-glucono-1,5-lactone semicarbazone series of inhibitors [12]. In this previous study, it was found that Glide native ligand redocking calculations (PDB code: 5UHO) did not reproduce the PUGNAc crystallographic conformation, specifically the orientation of the phenylurethane moiety, so that the Prime refinement and a QM/MM approach was applied [12]. In the present case, however, due to the shortened length of the linker and the greater variance in the structures of the target 2-acetamido-2-deoxy-d-glucono-1,5-lactone arenesulfonylhydrazones compared to PUGNAc in the starting model hOGA–PUGNAc complex (PDB code: 5UHO), refinements were performed in the hierarchical optimization mode (as opposed to the local optimization mode), which involved systematic sampling of inhibitor positions, orientations and conformations, along with enzyme binding site residues. The five output models for each inhibitor were then further refined in the local optimization mode (releasing hydrogen bond constraints, cf. Experimental details), but also used in QM/MM optimizations that should, in theory, better describe the energetic features of the predicted binding modes. More specifically, QM/MM has important applications in areas where standard force field-based methods may not be entirely accurate. A QM description of this novel set of compounds would address any potential shortcomings in force field parameters, particularly in the description of key dihedrals such as the rotations around the N1–N2 bond (cf. atom numbering scheme in Table 4) and the surrounding dihedrals. The binding of **5a**–**f** to hOGA was predicted using this approach; the PUGNAc inhibitor was also included for initial validation by geometrically comparing its predicted complex with its solved crystallographic structure (PDB code: 5UHO).

The energy results from the Prime enzyme–inhibitor refinements and QM/MM calculations are shown in Table 4. In some cases, close to equivalent poses were obtained and coincided with similar absolute/relative energies. As expected, all the energies are lower following the second Prime enzyme–inhibitor refinement in the local optimization mode; the lowest energy enzyme–inhibitor complex pose was also subject to change in some cases. As mentioned, QM/MM approaches can provide a more accurate picture of the preferred binding mode [22,23]. For PUGNAc, the lowest energy QM/MM optimized poses 4 and 5 were equivalent based on energy and RMSD (heavy atoms) comparisons. Comparing these predicted enzyme–inhibitor models with the crystal structure complex following backbone superimposition, the RMSDs (heavy atoms) for ligand and flexible binding site residues were just 0.762 Å and 0.452 Å, respectively, an initial validation of the refinement protocol. For **5a**–**f**, with the inhibitors described by QM, the key conformations through rotations around the N1–N2 bond (and associated dihedrals) can be more accurately described. Therefore, it was not overly surprising that there was a more considerable change in preferred binding mode following the QM/MM optimizations compared to the Prime enzyme–inhibitor refinements, both in terms of geometry and associated energetics. Most importantly, all inhibitors were observed to have a preference for the *s-cis* binding conformation around the N1–N2 bond (cf. Table 4), conformations that were originally ranked lower in the initial Prime refinements (where the preference was generally *s-trans*). The QM/MM optimized lowest energy complexes had inhibitor C1′=N1-N2-H dihedral angles in a similar range −168.0° to −172.2° for **5a**–**e**, and slightly less planar (−154.4°) for **5f**. It is noted that solvation effects are not accounted for in the QM/MM optimizations.

**Figure 2:**
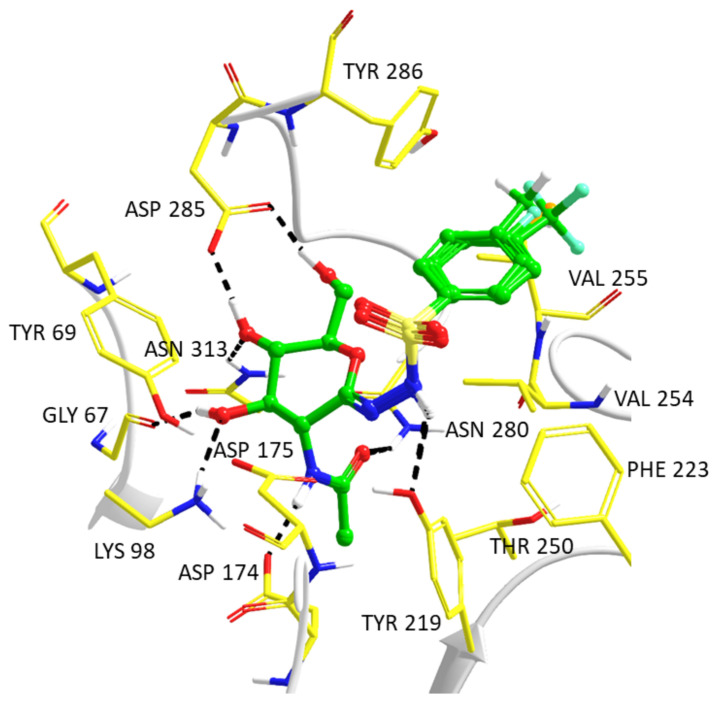
Predicted model complexes of the phenyl-substituted analogs **5a**–**d** following QM/MM optimizations of complexes from Prime hierarchical optimization mode protein–ligand refinements. Shown are the relative positions of the inhibitors following enzyme backbone superimposition; the protein from the predicted hOGA-**5a** complex only is displayed for clarity. Fluorine ligand atoms are shown as cyan and chlorine as orange.

The predicted QM/MM optimized binding geometries of the phenyl-substituted inhibitors **5a**–**d** with hOGA are similar and shown in Figure 2. There are key interactions similar to those reported previously for the 2-acetamido-2-deoxy-d-glucono-1,5-lactone semicarbazone series of compounds and PUGNAc [12]. Notably, the *N*-acetyl-glucosamine moiety hydrogen bond interactions are conserved as follows (cf. atom numbering in Table 4): inhibitor O3′-hydroxyl with both Gly67 backbone O and Lys98 sidechain; O4′-hydroxyl O with Asn313 sidechain amide and Asp285 sidechain carboxylate; O6′ hydroxyl with Asp285 sidechain carboxylate; and the *N*-acetyl group has hydrogen bonds involving N2′H with the Asp174 sidechain carboxylate and acetyl carbonyl O with the Asn280 sidechain amide. In terms of the sulfonylhydrazone linker between the *N*-acetyl-glucosamine and phenyl groups, N2H hydrogen bonds with the Tyr219 sidechain hydroxyl O atom and there is also potential for hydrogen bonding of this hydroxyl with ligand N1 atoms. The differences in **5a–d** are their phenyl *para*-substituents. All ligand phenyls have favorable interactions with the Val254 sidechain. There are interactions between the *para*-substituents (-CH_3_, -CF_3_, -F, and -Cl) and hydrophobic sidechains of Tyr286 and Val255. The CF_3_ group (**5b**) is shifted away from Tyr286, an interaction which may be related to its lower hOGA inhibition potency (*K*_i_ = 230 nM); alternatively, the methyl-substituted **5a** (*K*_i_ = 78 nM) can form good CH–π contacts with the Tyr286 ring. There are also NH–halogen (enzyme–inhibitor, respectively) interactions for **5b**–**d** involving the Val255 backbone NH. Notably, in the case of the most potent phenyl-substituted compound **5d** (Cl substituent, *K*_i_ = 70 nM), the halogen–hydrogen bond donor (HBD) interactions are consistent with a survey of crystal structure data; the predicted C–Cl–HBD angle (85.4°) and the HBD–Cl (4.4 Å) distance are close to the observed most prominent values (HBD defined by the NH nitrogen atom) [24].

**Figure 3:**
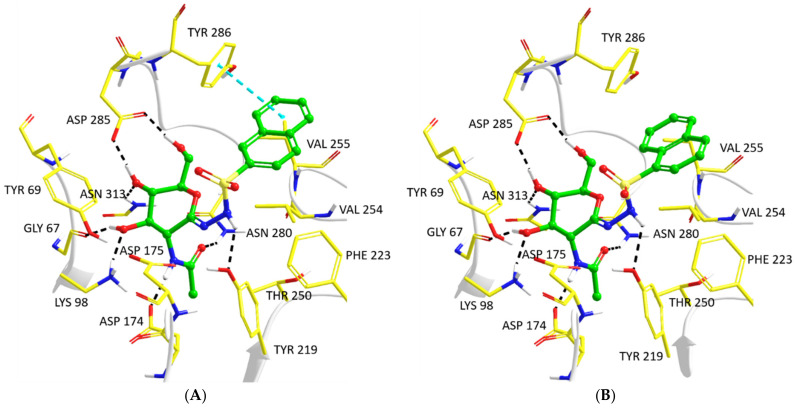
Predicted models of hOGA-**5e** (**A**) and hOGA-**5f** (**B**) following QM/MM optimizations of complexes from Prime hierarchical optimization mode enzyme–inhibitor refinements.

The most potent hOGA inhibitors were the naphthyl analogs, **5e** (2-naphthyl) and **5f** (1-naphthyl), with these ligands close to equipotent (*K*_i_-s ~ 30 nM). The predicted binding of these inhibitors from the QM/MM optimizations are shown in Figure 3A,B, respectively. Both ligands are able to exploit favorable interactions with the Val254 sidechain and have the potential for NH–π contacts from the backbone NH of the flexible loop residue Val255, all of which could be the source of their observed superior potencies. The 2-naphthyl substituent of **5e** is also aligned with the Tyr286 ring in a T-shaped configuration for π–π stacking interactions with a ring centroid–centroid distance of 5.1 Å. This can be considered consistent with geometric preferences of protein–ligand T-shaped π–π interactions from Protein Data Bank analysis, with T-shaped binding conformations also found to be the most predominant [25]. In the case of **5f**, a 180° flip of the 1-naphthalene ring around the S–C(Ar) bond would lead to alternate π–π interactions with Phe223.

## 3. Materials and Methods

### 3.1. Synthesis

#### 3.1.1. General Methods

Melting points were measured on a Kofler hot stage and are uncorrected. Optical rotations were determined using a Jasco P-2000 (Easton, MD, USA) polarimeter at room temperature. 1D NMR spectra were recorded using Bruker AM Avance 360 (360/90 MHz for ^1^H/^13^C) or Bruker 400 (400/100 MHz for ^1^H/^13^C) spectrometers (Bruker, Karlsruhe, Germany). Chemical shifts are referenced to TMS as an internal reference (^1^H) or to residual solvent signals (^13^C). 2D ^1^H-^13^C HSQC-CLIP-COSY experiment with HSQC vs. COSY peak sign editing [26] and 2D ^1^H-^1^H EASY ROESY experiment [19] with 300 ms mixing time were carried out for the unambiguous structure elucidation of compound **5d** on a Bruker Avance Neo 700 MHz NMR spectrometer (Bruker, Karlsruhe, Germany) equipped with a Prodigy TCI cryoprobe. Mass spectra were recorded with MicroTOF-Q type Qq-TOF MS and maXis II UHR ESI-QTOF MS (Bruker Daltonik, Bremen, Germany) instruments in the positive ion mode with the electrospray ionization or atmospheric pressure chemical ionization technique. TLC plates were visualized under UV light and by spray reagent with gentle heating (the plate was sprayed with the following solution: abs. EtOH (95 mL), conc. H_2_SO_4_ (5 mL), and anisaldehyde (1 mL)). Kieselgel 60 (Merck, particle size 0.063–0.200 mm) was used for column chromatography. CH_2_Cl_2_ was distilled from P_2_O_5_ and stored over 4 Å molecular sieves. *p*-Toluenesulfonyl hydrazide (**2a**) was purchased from Sigma-Aldrich Ltd. (Budapest, Hungary). 2-Acetamido-3,4,6-tri-*O*-acetyl-2-deoxy-α,β-d-glucopyranose (**1**) [16,17], substituted benzenesulfonyl hydrazides (**2b-d**), and 2- or 1-naphthalenesulfonyl hydrazides (**2e-f**) [18] were synthesized according to literature procedures. Unless otherwise indicated, all chemicals used in the biochemical experiments were of analytical grade and were purchased from Sigma-Aldrich Ltd. (Budapest, Hungary).

#### 3.1.2. General Procedure A for the Synthesis of 1-(2-Acetamido-3,4,6-tri-*O*-acetyl-2-deoxy-β-d-glucopyranosyl)-2-arenesulfonyl Hydrazines (**3**)

A solution of 200 mg (0.58 mmol) of 2-acetamido-3,4,6-tri-*O*-acetyl-2-deoxy-α,β-d-glucopyranose (**1**), 0.87 mmol of an arenesulfonyl hydrazide **2** and 0.058 mmol of *p*-toluenesulfonic acid in 4 mL of chloroform was boiled under reflux for 4 h until the reaction was complete (monitored by TLC hexane:acetone, 1:1). The mixture was cooled in an ice bath and the white precipitate was filtered off. If the reagents contaminated the product, column chromatography was performed (hexane:acetone, 3:2).

##### 1-(2-Acetamido-3,4,6-tri-*O*-acetyl-2-deoxy-β-d-glucopyranosyl)-2-(*p*-toluenesulfonyl) Hydrazine (**3a**)

Prepared according to General procedure A from 200 mg (0.58 mmol) of **1**, 160 mg (0.87 mmol) of *p*-toluenesulfonyl hydrazide (**2a**) and 12 mg (0.058 mmol) *p*-toluenesulfonic acid. The excess of the reagent was filtered off from the reaction mixture and the solvent was evaporated: 230 mg (77%) of white powder was isolated. m.p.: 172–174 °C; [α]_D_ = −60 (c = 0.43, CHCl_3_). ^1^H NMR (CDCl_3_) *δ* (ppm): 7.72 (d, 2H, *J* = 7.7 Hz, Ar), 7.31 (d, 2H, *J* = 7.6 Hz, Ar), 6.65 (s, 1H, NH), 6.13 (d, 1H, *J* = 8.5 Hz, NH), 5.28 (pt, 1H, *J* = 9.8 Hz, H-3), 4.98 (pt, 1H, *J* = 9.6 Hz, H-4), 4.41 (pt, 1H, *J* = 8.4 Hz, H-1), 4.28 (d, 1H, *J* = 7.9 Hz, NH), 4.19 (dd, 1H, *J* = 3.8, 12.1 Hz, H-6a), 4.09 (dd, 1H, *J* = 1.8, 12.1 Hz, H-6b), 3.86 (ddd (’q’), 1H, *J* = 9.4 Hz, H-2), 3.70–3.66 (m, 1H, H-5), 2.43 (s, 3H, CH_3_), 2.08, 2.03, 2.02, 1.99 (4 s, 12H, 4 CH_3_). ^13^C NMR (CDCl_3_) *δ* (ppm): 171.19, 171.09, 170.84, 169.54 (C=O), 144.42, 134.77, 129.68, 128.15 (Ar), 90.10 (C-1), 73.10, 72.59, 68.63 (C-3, C-4, C-5), 62.16 (C-6), 51.97 (C-2), 23.30, 21.68, 20.83, 20.77, 20.69 (5 CH_3_). ESI-MS positive mode (*m/z*): calcd. for C_21_H_29_N_3_O_10_S (515.16) [M + H]^+^ = 516.16, found: [M + H]^+^ = 516.92.

##### 1-(2-Acetamido-3,4,6-tri-*O*-acetyl-2-deoxy-β-d-glucopyranosyl)-2-(*p*-trifluoromethylbenzenesulfonyl) Hydrazine (**3b**)

Prepared according to General procedure A from 200 mg (0.58 mmol) of **1**, 209 mg (0.87 mmol) of *p*-trifluoromethylbenzenesulfonyl hydrazide (**2b**) and 12 mg (0.058 mmol) *p*-toluenesulfonic acid. The excess of the reagent was filtered off from the reaction mixture, the solvent was removed and the residue was purified by column chromatography: 281 mg (85%) of white powder was isolated. m.p.: 185–187 °C (decomposition); [α]_D_ = −41 (c = 0.32, CH_2_Cl_2_). ^1^H NMR (CDCl_3_) *δ* (ppm): 7.98 (d, 2H, *J* = 7.7 Hz, Ar), 7.79 (t, 2H, *J* = 7.7 Hz, Ar), 6.85 (s, 1H, NH), 6.08 (d, 1H, *J* = 8.3 Hz, NH), 5.23 (pt, 1H, *J* = 9.7 Hz, H-3), 5.00 (pt, 1H, *J* = 9.4 Hz, H-4), 4.46–4.28 (m, 2H, H-1, NH), 4.19 (dd, 1H, *J* = 2.9, 11.7 Hz, H-6a), 4.11 (dd, 1H, *J* = 1.3, 12.2 Hz, H-6b), 3.87 (ddd (’q’), 1H, *J* = 8.8 Hz, H-2), 3.77–3.62 (m, 1H, H-5), 2.08, 2.04, 2.03, 1.98 (4 s, 12H, 4 CH_3_). ^13^C NMR (CDCl_3_) *δ* (ppm): 171.32, 171.16, 170.85, 169.51 (C=O), 141.58, 134.94 (q, ^2^*J*_CF_ = 33.6 Hz), 128.63, 126.26 (q, ^4^*J*_CF_ = 3.6 Hz, Ar) 126.07 (q, ^1^*J*_CF_ = 280.6 Hz, *C*F_3_), 90.47 (C-1), 73.37, 72.52, 68.45 (C-3, C-4, C-5), 62.10 (C-6), 52.13 (C-2), 23.33, 20.87, 20.79, 20.71 (4 CH_3_). ESI-MS positive mode (*m*/*z*): calcd. for C_21_H_26_F_3_N_3_O_10_S (569.13) [M + H]^+^ = 570.14, found: [M + H]^+^ = 570.17.

##### 1-(2-Acetamido-3,4,6-tri-*O*-acetyl-2-deoxy-β-d-glucopyranosyl)-2-(*p*-fluorobenzenesulfonyl) Hydrazine (**3c**)

Prepared according to General procedure A from 200 mg (0.58 mmol) of **1**, 165 mg (0.87 mmol) of *p*-fluorobenzenesulfonyl hydrazide (**2c**), and 12 mg (0.058 mmol) *p*-toluenesulfonic acid. The excess of the reagent was filtered off from the reaction mixture, the solvent was removed, and the residue was purified by column chromatography: 259 mg (86%) of white powder was isolated. m.p.: 187–190 °C; [α]_D_ = −38 (c = 0.30, CH_2_Cl_2_). ^1^H NMR (CDCl_3_) *δ* (ppm): 7.86 (dd, 2H, *J* = 5.0, 8.7 Hz, Ar), 7.20 (t, 2H, *J* = 8.5 Hz, Ar), 6.63 (s, 1H, NH), 6.04 (d, 1H, *J* = 8.5 Hz, NH), 5.24 (pt, 1H, *J* = 9.9 Hz, H-3), 5.00 (pt, 1H, *J* = 9.7 Hz, H-4), 4.40 (pt, 1H, *J* = 9.1 Hz, H-1), 4.30 (d, 1H, *J* = 9.0 Hz, NH), 4.20 (dd, 1H, *J* = 4.5, 12.3 Hz, H-6a), 4.09 (dd, 1H, *J* = 1.2, 12.0 Hz, H-6b), 3.86 (ddd (’q’), 1H, *J* = 9.5 Hz, H-2), 3.68 (ddd, 1H, *J* = 2.2, 4.2, 9.9 Hz, H-5), 2.08, 2.04, 2.03, 1.98 (4 s, 12H, 4 CH_3_). ^13^C NMR (CDCl_3_) *δ* (ppm): 171.28, 171.08, 170.83, 169.52 (C=O), 165.66 (d, ^1^*J*_CF_ = 255.8 Hz), 133.86, 130.92 (d, ^3^*J*_CF_ = 9.3 Hz), 116.41 (d, ^2^*J*_CF_ = 22.7 Hz, Ar), 90.34 (C-1), 73.29, 72.52, 68.44 (C-3, C-4, C-5), 62.11 (C-6), 52.10 (C-2), 23.36, 20.88, 20.81, 20.73 (4 CH_3_). ESI-MS positive mode (*m/z*): calcd. for C_20_H_26_FN_3_O_10_S (519.13) [M + H]^+^ = 520.14, found: [M + H]^+^ = 520.17.

##### 1-(2-Acetamido-3,4,6-tri-*O*-acetyl-2-deoxy-β-d-glucopyranosyl)-2-(*p*-chlorobenzenesulfonyl) Hydrazine (**3d**)

Prepared according to General procedure A from 200 mg (0.58 mmol) of **1**, 180 mg (0.87 mmol) of *p*-chlorobenzenesulfonyl hydrazide (**2d**), and 12 mg (0.058 mmol) *p*-toluenesulfonic acid. The excess of the reagent was filtered off from the reaction mixture, the solvent was removed, and the residue was purified by column chromatography: 277 mg (89%) of white powder was isolated. m.p.: 195–198 °C (decomposition); [α]_D_ = −42 (c = 0.23, CH_2_Cl_2_). ^1^H NMR (CDCl_3_) *δ* (ppm): 7.77 (d, 2H, *J* = 8.6 Hz, Ar), 7.49 (d, 2H, *J* = 8.6 Hz, Ar), 6.46 (s, 1H, NH), 5.85 (d, 1H, *J* = 8.5 Hz, NH), 5.22 (pt, 1H, *J* = 10.0 Hz, H-3), 5.01 (pt, 1H, *J* = 9.7 Hz, H-4), 4.39 (pt, 1H, *J* = 9.1 Hz, H-1), 4.26 (d, 1H, *J* = 8.9 Hz, NH), 4.20 (dd, 1H, *J* = 4.6, 12.4 Hz, H-6a), 4.10 (dd, 1H, *J* = 1.9, 12.3 Hz, H-6b), 3.86 (ddd (’q’), 1H, *J* = 9.6 Hz, H-2), 3.66 (ddd, 1H, *J* = 2.4, 4.6, 10.2 Hz, H-5), 2.09, 2.04, 2.03, 1.98 (4 s, 12H, 4 CH_3_). ^13^C NMR (CDCl_3_) *δ* (ppm): 171.34, 171.00, 170.78, 169.49 (C=O), 140.22, 136.38, 129.55, 129.47 (Ar), 90.41 (C-1), 73.43, 72.52, 68.35 (C-3, C-4, C-5), 62.08 (C-6), 52.18 (C-2), 23.40, 20.90, 20.81, 20.74 (4 CH_3_). ESI-MS positive mode (*m/z*): calcd. for C_20_H_26_ClN_3_O_10_S (535.10) [M + H]^+^ = 536.11, found: [M + H]^+^ = 536.17.

##### 1-(2-Acetamido-3,4,6-tri-*O*-acetyl-2-deoxy-β-d-glucopyranosyl)-2-(2-naphthalenesulfonyl) Hydrazine (**3e**)

Prepared according to General procedure A from 200 mg (0.58 mmol) of **1**, 193 mg (0.87 mmol) of 2-naphthalenesulfonyl hydrazide (**2e**), and 12 mg (0.058 mmol) *p*-toluenesulfonic acid. The excess of the reagent was filtered off from the reaction mixture, the solvent was removed, and the residue was purified by column chromatography: 243 mg (76%) of white powder was isolated. m.p.: 145–148 °C; [α]_D_ = −33 (c = 0.23, CH_2_Cl_2_). ^1^H NMR (CDCl_3_) *δ* (ppm): 8.41 (s, 1H, Ar), 7.97–7.86 (m, 3H, Ar), 7.80 (dd, 1H, *J* = 1.8, 8.7 Hz, Ar), 7.65 (td, 1H, *J* = 1.6, 6.8 Hz, Ar), 7.60 (td, 1H, *J* = 1.2, 6.5 Hz, Ar), 6.99 (s, 1H, NH), 6.30 (d, 1H, *J* = 8.9 Hz, NH), 5.26 (pt, 1H, *J* = 10.1 Hz, H-3), 4.96 (pt, 1H, *J* = 9.8 Hz, H-4), 4.45–4.37 (m, 2H, H-1, NH), 4.15 (dd, 1H, *J* = 4.5, 12.4 Hz, H-6a), 4.06 (dd, 1H, *J* = 2.2, 12.3 Hz, H-6b), 3.86 (ddd (’q’), 1H, *J* = 9.3 Hz, H-2), 3.66 (ddd, 1H, *J* = 2.3, 4.3, 10.0 Hz, H-5), 2.04, 2.02, 2.00, 1.95 (4 s, 12H, 4 CH_3_). ^13^C NMR (CDCl_3_) *δ* (ppm): 171.22, 171.07, 170.87, 169.54 (C=O), 135.17, 134.66, 132.09, 129.84, 129.33, 129.30, 129.20, 128.06, 127.73, 122.95 (Ar), 90.17 (C-1), 73.05, 72.62, 68.62 (C-3, C-4, C-5), 62.11 (C-6), 51.94 (C-2), 23.25, 20.80, 20.75, 20.67 (4 CH_3_). ESI-MS positive mode (*m/z*): calcd. for C_24_H_29_N_3_O_10_S (551.16) [M + H]^+^ = 552.16, found: [M + H]^+^ = 552.08.

##### 1-(2-Acetamido-3,4,6-tri-*O*-acetyl-2-deoxy-β-d-glucopyranosyl)-2-(1-naphthalenesulfonyl) Hydrazine (**3f**)

Prepared according to General procedure A from 200 mg (0.58 mmol) of **1**, 193 mg (0.87 mmol) of 1-naphthalenesulfonyl hydrazide (**2f**), and 12 mg (0.058 mmol) *p*-toluenesulfonic acid. The excess of the reagent was filtered off from the reaction mixture, the solvent was removed, and the residue was purified by column chromatography: 285 mg (89%) of colorless foam was isolated. [α]_D_ = −19 (c = 0.22, CH_2_Cl_2_). ^1^H NMR (CDCl_3_) *δ* (ppm): 8.64 (d, 1H, *J* = 8.6 Hz, Ar), 8.24 (dd, 1H, *J* = 1.1, 7.4 Hz, Ar), 8.11 (d, 1H, *J* = 8.2 Hz, Ar), 7.95 (d, 1H, *J* = 7.6 Hz, Ar), 7.67 (td, 1H, *J* = 1.4, 7.1 Hz, Ar), 7.61 (td, 1H, *J* = 1.2, 8.1 Hz, Ar), 7.53 (t, 1H, *J* = 7.6 Hz, Ar), 6.96 (s, 1H, NH), 5.99 (d, 1H, *J* = 8.9 Hz, NH), 5.14 (pt, 1H, *J* = 10.0 Hz, H-3), 4.87 (pt, 1H, *J* = 9.7 Hz, H-4), 4.33 (d, 1H, *J* = 8.0 Hz, NH), 4.26 (t, 1H, *J* = 9.0 Hz, H-1), 4.05 (dd, 1H, *J* = 4.5, 12.4 Hz, H-6a), 4.00 (dd, 1H, *J* = 2.5, 12.4 Hz, H-6b), 3.79 (ddd (’q’), 1H, *J* = 9.2 Hz, H-2), 3.54 (ddd, 1H, *J* = 2.6, 4.3, 10.1 Hz, H-5), 2.06, 2.00, 1.99, 1.90 (4 s, 12H, 4 CH_3_). ^13^C NMR (CDCl_3_) *δ* (ppm): 171.15, 171.05, 170.80, 169.47 (C=O), 135.23, 134.27, 132.55, 131.58, 129.24, 128.59, 128.43, 127.11, 124.65, 124.02 (Ar), 89.94 (C-1), 73.06, 72.58, 68.49 (C-3, C-4, C-5), 62.12 (C-6), 51.83 (C-2), 23.23, 20.85, 20.75, 20.67 (4 CH_3_). ESI-MS positive mode (*m/z*): calcd. for C_24_H_29_N_3_O_10_S (551.16) [M + H]^+^ = 552.16, found: [M + H]^+^ = 552.08.

#### 3.1.3. General Procedure B for the Synthesis of 2-Acetamido-3,4,6-tri-*O*-acetyl-2-deoxy-d-glucono-1,5-lactone Arenesulfonylhydrazones (**4**)

Activated MnO_2_ (quantity specified with the particular compounds) was added to a solution of 100 mg of compound **3** in anhydrous CH_2_Cl_2_. The mixture was boiled for 20 h. After the reaction was completed (TLC hexane:acetone, 1:1), the MnO_2_ was filtered off under diminished pressure, and the filtrate was concentrated. The residue was purified by column chromatography (hexane:acetone, 2:1).

##### 2-Acetamido-3,4,6-tri-*O*-acetyl-2-deoxy-d-glucono-1,5-lactone *p*-Toluenesulfonylhydrazone (**4a**)

Compound **4a** was obtained according to General procedure B from **3a** (100 mg, 0.194 mmol) in 4 mL of anhydrous CH_2_Cl_2_, and 340 mg (3.88 mmol) of activated MnO_2_. The crude product was purified by column chromatography to give 69 mg (69%) of white powder. m.p.: 137–141 °C; [α]_D_ = +15 (c = 0.48, CHCl_3_). ^1^H NMR (CDCl_3_) *δ* (ppm): 8.07 (s, 1H, NH), 7.75 (d, 2H, *J* = 8.3 Hz, Ar), 7.31 (d, 2H, *J* = 8.3 Hz, Ar), 6.15 (d, 1H, *J* = 8.7 Hz, NH), 5.29 (pt, 1H, *J* = 9.2 Hz, H-3), 5.19 (pt, 1H, *J* = 9.2 Hz, H-4), 4.76 (dd, 1H, *J* = 8.7, 9.2 Hz, H-2), 4.30–4.21 (m, 3H, H-6a, H-6b, H-5), 2.43 (s, 3H, CH_3_), 2.08, 2.04, 2.04, 2.00 (4 s, 12H, 4 CH_3_). ^13^C NMR (CDCl_3_) *δ* (ppm): 170.51, 170.47, 170.42, 169.19 (C=O), 146.12 (C=N), 144.55, 134.97, 129.74, 127.99 (Ar), 77.05, 71.81, 67.19 (C-3, C-4, C-5), 61.29 (C-6), 50.20 (C-2), 23.09, 21.74, 20.73, 20.60 (5 CH_3_). ESI-MS positive mode (*m/z*): calcd. for C_21_H_27_N_3_O_10_S (513.14) [M + H]^+^ = 514.15, found: [M + H]^+^ = 514.25.

##### 2-Acetamido-3,4,6-tri-*O*-acetyl-2-deoxy-d-glucono-1,5-lactone *p*-Trifluoromethylbenzenesulfonylhydrazone (**4b**)

Compound **4b** was obtained according to General procedure B from **3b** (100 mg, 0.176 mmol) in 4 mL anhydrous CH_2_Cl_2_ and 306 mg (3.52 mmol) of activated MnO_2_. The crude product was purified by column chromatography to give 60 mg (60%) of white powder. m.p.: 182–185 °C (decomposition); [α]_D_ = +16 (c = 0.25, CH_2_Cl_2_). ^1^H NMR (CDCl_3_) *δ* (ppm): 8.21 (s, 1H, NH), 8.01 (d, 2H, *J* = 8.2 Hz, Ar), 7.79 (d, 2H, *J* = 8.4 Hz, Ar), 6.12 (d, 1H, *J* = 8.6 Hz, NH), 5.29 (pt, 1H, *J* = 9.5 Hz, H-3), 5.22 (pt, 1H, *J* = 9.6 Hz, H-4), 4.73 (dd, 1H, *J* = 8.7, 9.7 Hz, H-2), 4.38–4.25 (m, 3H, H-6a, H-6b, H-5), 2.07, 2.05, 2.05, 1.99 (4 s, 12H, 4 CH_3_). ^13^C NMR (CDCl_3_) *δ* (ppm): 170.60, 170.56, 170.43, 169.20 (C=O), 146.95 (C=N), 141.63, 135.06 (q, ^2^*J*_CF_ = 33.3 Hz), 128.50, 126.24 (q, ^4^*J*_CF_ = 3.4 Hz, Ar) 123.26 (q, ^1^*J*_CF_ = 271.7 Hz, *C*F_3_), 77.13, 71.50, 67.25 (C-3, C-4, C-5), 61.31 (C-6), 50.31 (C-2), 22.99, 20.67, 20.55 (4 CH_3_). ESI-MS positive mode (*m/z*): calcd. for C_21_H_24_F_3_N_3_O_10_S (567.11) [M + H]^+^ = 568.12, found: [M + H]^+^ = 568.14.

##### 2-Acetamido-3,4,6-tri-*O*-acetyl-2-deoxy-d-glucono-1,5-lactone *p*-Fluorobenzenesulfonylhydrazone (**4c**)

Compound **4c** was obtained according to General procedure B from **3c** (100 mg, 0.193 mmol) in 4 mL anhydrous CH_2_Cl_2_ and 336 mg (3.86 mmol) of activated MnO_2_. The crude product was purified by column chromatography to give 72 mg (72%) of amorphous foam. [α]_D_ = +14 (c = 0.24, CH_2_Cl_2_). ^1^H NMR (CDCl_3_) *δ* (ppm): 8.16 (s, 1H, NH), 7.89 (dd, 2H, *J* = 5.0, 8.9 Hz, Ar), 7.19 (t, 2H, *J* = 8.6 Hz, Ar), 6.20 (d, 1H, *J* = 8.6 Hz, NH), 5.30 (pt, 1H, *J* = 9.1 Hz, H-3), 5.21 (pt, 1H, *J* = 9.6 Hz, H-4), 4.75 (dd, 1H, *J* = 8.7, 10.0 Hz, H-2), 4.38–4.22 (m, 3H, H-6a, H-6b, H-5), 2.08, 2.05, 2.04, 2.00 (4 s, 12H, 4 CH_3_). ^13^C NMR (CDCl_3_) δ (ppm): 170.55, 170.48, 170.45, 169.19 (C=O), 146.48 (C=N), 165.68 (d, ^1^*J*_CF_ = 256.1 Hz), 134.01 (d, ^4^*J*_CF_ = 3.1 Hz), 130.80 (d, ^3^*J*_CF_ = 9.4 Hz), 116.41 (d, ^2^*J*_CF_ = 22.7 Hz, Ar), 77.12, 71.61, 67.23 (C-3, C-4, C-5), 61.30 (C-6), 50.26 (C-2), 23.07, 20.73, 20.69, 20.59 (4 CH_3_). ESI-MS positive mode (*m/z*): calcd. for C_20_H_24_FN_3_O_10_S (517.12) [M + H]^+^ = 518.12, found: [M + H]^+^ = 518.17.

##### 2-Acetamido-3,4,6-tri-*O*-acetyl-2-deoxy-d-glucono-1,5-lactone *p*-Chlorobenzenesulfonylhydrazone (**4d**)

Compound **4d** was obtained according to General procedure B from **3d** (100 mg, 0.187 mmol) in 4 mL anhydrous CH_2_Cl_2_ and 325 mg (3.74 mmol) of activated MnO_2_. The crude product was purified by column chromatography to give 77 mg (77%) of amorphous foam. [α]_D_ = +24 (c = 0.25, CH_2_Cl_2_). ^1^H NMR (CDCl_3_) *δ* (ppm): 8.19 (s, 1H, NH), 7.80 (d, 2H, *J* = 8.6 Hz, Ar), 7.49 (d, 2H, *J* = 8.6 Hz, Ar), 6.21 (d, 1H, *J* = 8.7 Hz, NH), 5.30 (pt, 1H, *J* = 9.1 Hz, H-4), 5.22 (pt, 1H, *J* = 9.6 Hz, H-3), 4.74 (dd, 1H, *J* = 8.7, 9.6 Hz, H-2), 4.36–4.20 (m, 3H, H-6a, H-6b, H-5), 2.08, 2.05, 2.04, 2.00 (4 s, 12H, 4 CH_3_). ^13^C NMR (CDCl_3_) *δ* (ppm): 170.49, 170.43, 170.37, 169.14 (C=O), 146.58 (C=N), 140.10, 136.39, 129.36, (Ar), 77.02, 71.49, 67.14 (C-3, C-4, C-5), 61.23 (C-6), 50.21 (C-2), 23.01, 20.66, 20.64, 20.53 (4 CH_3_). ESI-MS positive mode (*m/z*): calcd. for C_20_H_24_ClN_3_O_10_S (533.09) [M + H]^+^ = 534.09, found: [M + H]^+^ = 534.08.

##### 2-Acetamido-3,4,6-tri-*O*-acetyl-2-deoxy-d-glucono-1,5-lactone 2-Naphthalenesulfonylhydrazone (**4e**)

Compound **4e** was obtained according to General procedure B from **3e** (100 mg, 0.181 mmol) in 4 mL anhydrous CH_2_Cl_2_ and 315 mg (3.62 mmol) of activated MnO_2_. The crude product was purified by column chromatography to give 56 mg (56 %) of amorphous foam. [α]_D_ = +26 (c = 0.25, CH_2_Cl_2_). ^1^H NMR (CDCl_3_) *δ* (ppm): 8.47 (s, 1H, Ar), 8.06 (s, 1H, NH), 8.00–7.88 (m, 3H, Ar), 7.84 (dd, 1H, *J* = 1.8, 8.7 Hz, Ar), 7.70–7.57 (m, 2H, Ar), 6.01 (d, 1H, *J* = 8.7 Hz, NH), 5.26 (pt, 1H, *J* = 9.0 Hz, H-4), 5.17 (pt, 1H, *J* = 9.6 Hz, H-3), 4.72 (dd, 1H, *J* = 8.8, 9.7 Hz, H-2), 4.33–4.16 (m, 3H, H-6a, H-6b, H-5), 2.04, 2.03, 2.01, 1.94 (4 s, 12H, 4 CH_3_). ^13^C NMR (CDCl_3_) *δ* (ppm): 170.50, 170.43, 169.18 (C=O), 146.26 (C=N), 135.35, 134.92, 132.22, 129.65, 129.47, 129.39, 129.31, 128.14, 127.79, 122.88 (Ar), 77.21, 71.78, 67.22 (C-3, C-4, C-5), 61.33 (C-6), 50.28 (C-2), 23.06, 20.70, 20.60 (4 CH_3_). ESI-MS positive mode (*m/z*): calcd. for C_24_H_27_N_3_O_10_S (549.14) [M + H]^+^ = 550.15, found: [M + H]^+^ = 550.17.

##### 2-Acetamido-3,4,6-tri-*O*-acetyl-2-deoxy-d-glucono-1,5-lactone 1-Naphthalenesulfonylhydrazone (**4f**)

Compound **4f** was obtained according to General procedure B from **3f** (100 mg, 0.181 mmol) in 4 mL anhydrous CH_2_Cl_2_ and 315 mg (3.62 mmol) of activated MnO_2_. The crude product was purified by column chromatography to give 66 mg (66%) of amorphous foam. [α]_D_ = −19 (c = 0.22, CH_2_Cl_2_). ^1^H NMR (CDCl_3_) *δ* (ppm): 8.76 (d, 1H, *J* = 8.6 Hz, Ar), 8.49 (s, 1H, NH), 8.29 (dd, 1H, *J* = 1.1, 7.4 Hz, Ar), 8.11 (d, 1H, *J* = 8.3 Hz, Ar), 7.95 (d, 1H, *J* = 7.5 Hz, Ar), 7.71–7.51 (m, 3H, Ar), 6.09 (d, 1H, *J* = 8.6 Hz, NH), 5.23 (pt, 1H, *J* = 9.1 Hz, H-4), 5.10 (pt, 1H, *J* = 9.9 Hz, H-3), 4.68 (dd, 1H, *J* = 8.7, 10.0 Hz, H-2), 4.33–4.20 (m, 2H, H-6a, H-6b), 4.16 (ddd, 1H, *J* = 2.8, 5.8, 9.2 Hz, H-5), 2.03, 2.00, 1.98, 1.86 (4 s, 12H, 4 CH_3_). ^13^C NMR (CDCl_3_) *δ* (ppm): 170.47, 170.32, 170.29, 169.14 (C=O), 146.16 (C=N), 135.24, 134.29, 133.34, 130.82, 129.21, 128.58, 128.45, 127.06, 124.75, 124.21 (Ar), 76.99, 71.94, 67.16 (C-3, C-4, C-5), 61.25 (C-6), 49.97 (C-2), 22.96, 20.67, 20.64, 20.54 (4 CH_3_). ESI-MS positive mode (*m/z*): calcd. for C_24_H_27_N_3_O_10_S (549.14) [M + H]^+^ = 550.15, found: [M + H]^+^ = 550.20.

#### 3.1.4. General Procedure C for the Synthesis of 2-Acetamido-2-deoxy-d-glucono-1,5-lactone Arenesulfonylhydrazones (**5**)

A solution of 100 mg of a compound **4** in 1 mL of MeOH was stirred at 25 °C and 1 mL of a saturated NH_3_/MeOH solution was added. After completion of the reaction (4 h, monitored by TLC, CHCl_3_: MeOH, 7: 3), the solvent was removed and the residue was purified by column chromatography (CHCl_3_: MeOH, 20: 1).

##### 2-Acetamido-2-deoxy-d-glucono-1,5-lactone *p*-Toluenesulfonylhydrazone (**5a**)

Prepared from **4a** (100 mg, 0.195 mmol) according to General procedure C. The crude product was purified by column chromatography to give 71 mg (94%) of white powder. m.p.: 165–166 °C; [α]_D_ = +55 (c = 0.55, MeOH); ^1^H NMR (MeOH-*d*_4_) *δ* (ppm): 7.73 (d, 2H, *J* = 8.3 Hz, Ar), 7.34 (d, 2H, *J* = 8.0 Hz, Ar), 4.31 (d, 1H, *J* = 10.1 Hz, H-2), 3.98 (dd, 1H, *J* = 5.8, 10.4 Hz, H-5), 3.76–3.64 (m, 2H, H-6a, H-6b), 3.56 (dd, 1H, *J* = 9.0, 10.0, Hz, H-3), 3.45 (t, 1H, *J* = 9.2 Hz, H-4), 2.41 (s, 3H, CH_3_), 2.02 (s, 3H, CH_3_). ^13^C NMR (MeOH-*d*_4_) *δ* (ppm): 174.08 (C=O), 149.44 (C=N), 145.53, 136.57, 130.58, 128.70 (Ar), 82.84, 74.18, 70.24 (C-3, C-4, C-5), 62.50 (C-6), 53.46 (C-2), 22.83 (CH_3_), 21.56 (CH_3_). ESI-HRMS positive mode (*m/z*): calcd. for C_15_H_21_N_3_O_7_S (387.1100) [M + Na]^+^ = 410.0992, found: [M + Na]^+^ = 410.0989.

##### 2-Acetamido-2-deoxy-d-glucono-1,5-lactone *p*-Trifluoromethylbenzenesulfonylhydrazone (**5b**)

Prepared from **4b** (100 mg, 0.176 mmol) according to General procedure C. The crude product was purified by column chromatography to give 65 mg (83%) of white powder. m.p: 175–176 °C (decomposition); [α]_D_ = +59 (c = 0.35, MeOH); ^1^H NMR (MeOH-*d*_4_) *δ* (ppm): 8.04 (d, 2H, *J* = 8.2 Hz, Ar), 7.87 (d, 2H, *J* = 8.3 Hz, Ar), 4.31 (d, 1H, *J* = 10.1 Hz, H-2), 3.99 (dd, 1H, *J* = 1.3, 11.7 Hz, H-5), 3.81–3.65 (m, 2H, H-6a, H-6b), 3.59 (dd, 1H, *J* = 9.1, 10.0, Hz, H-3), 3.46 (t, 1H, *J* = 9.2 Hz, H-4), 2.02 (s, 3H, CH_3_). ^13^C NMR (MeOH-*d*_4_) *δ* (ppm): 173.66 (C=O), 149.60 (C=N), 144.60, 135.12 (q, ^2^*J*_CF_ = 32.6 Hz), 129.52, 127.06 (q, ^4^*J*_CF_ = 3.6 Hz, Ar) 121.92 (q, ^1^*J*_CF_ = 271.9 Hz, *C*F_3_), 83.16, 74.30, 70.54 (C-3, C-4, C-5), 62.88 (C-6), 53.65 (C-2), 22.76 (CH_3_). ESI-HRMS positive mode (*m/z*): calcd. for C_15_H_18_F_3_N_3_O_7_S (441.0818) [M + Na]^+^ = 464.0710, found: [M + Na]^+^ = 464.0708.

##### 2-Acetamido-2-deoxy-d-glucono-1,5-lactone *p*-Fluorobenzenesulfonylhydrazone (**5c**)

Prepared from **4c** (100 mg, 0.193 mmol) according to General procedure C. The crude product was purified by column chromatography to give 64 mg (85%) of white powder. m.p.: 175–177 °C; [α]_D_ = +78 (c = 0.44, MeOH); ^1^H NMR (MeOH-*d*_4_) *δ* (ppm): 7.90 (dd, 2H, *J* = 5.2, 8.6 Hz, Ar), 7.28 (t, 2H, *J* = 8.7 Hz, Ar), 4.32 (d, 1H, *J* = 10.1 Hz, H-2), 4.00 (dd, 1H, *J* = 7.2, 10.9 Hz, H-5), 3.79–3.65 (m, 2H, H-6a, H-6b), 3.60 (dd, 1H, *J* = 8.3, 10.6, Hz, H-3), 3.47 (t, 1H, *J* = 9.0 Hz, H-4), 2.03 (s, 3H, CH_3_). ^13^C NMR (MeOH-*d*_4_) *δ* (ppm): 173.64 (C=O), 149.34 (C=N), 166.57 (d, ^1^*J*_CF_ = 252.6 Hz), 136.46 (d, ^4^*J*_CF_ = 3.1 Hz), 131.69 (d, ^3^*J*_CF_ = 9.5 Hz), 116.96 (d, ^2^*J*_CF_ = 23.0 Hz, Ar), 83.09, 74.34, 70.55 (C-3, C-4, C-5), 62.85 (C-6), 53.58 (C-2), 22.78 (CH_3_). ESI-HRMS positive mode (*m/z*): calcd. for C_14_H_18_FN_3_O_7_S (391.0849) [M + Na]^+^ = 414.0742, found: [M + Na]^+^ = 414.0738.

##### 2-Acetamido-2-deoxy-d-glucono-1,5-lactone *p*-Chlorobenzenesulfonylhydrazone (**5d**)

Prepared from **4d** (100 mg, 0.188 mmol) according to General procedure C. The crude product was purified by column chromatography to give 60 mg (78%) of white powder. m.p.: 180–182 °C (decomposition); [α]_D_ = +78 (c = 0.22, MeOH); ^1^H NMR (MeOH-*d*_4_) *δ* (ppm): 7.82 (d, 2H, *J* = 8.8 Hz, Ar), 7.56 (d, 2H, *J* = 8.8 Hz, Ar), 4.30 (d, 1H, *J* = 10.1 Hz, H-2), 3.97 (dd, 1H, *J* = 8.3, 10.5 Hz, H-5), 3.78–3.65 (m, 2H, H-6a, H-6b), 3.58 (dd, 1H, *J* = 9.0, 10.1, Hz, H-3), 3.46 (t, 1H, *J* = 9.2 Hz, H-4), 2.02 (s, 3H, CH_3_). ^13^C NMR (MeOH-*d*_4_) *δ* (ppm): 173.66 (C=O), 149.42 (C=N), 140.17, 139.07, 130.46, 130.17 (Ar), 83.16, 74.36, 70.58, (C-3, C-4, C-5), 62.90 (C-6), 53.63 (C-2), 22.76 (CH_3_). ESI-HRMS positive mode (*m*/*z*): calcd. for C_14_H_18_ClN_3_O_7_S (407.0554) [M + Na]^+^ = 430.0446, found: [M + Na]^+^ = 430.0440.

##### 2-Acetamido-2-deoxy-d-glucono-1,5-lactone 2-Naphthalenesulfonylhydrazone (**5e**)

Prepared from **4e** (100 mg, 0.182 mmol) according to General procedure C. The crude product was purified by column chromatography to give 58 mg (75%) of white powder. m.p.: 172–173 °C; [α]_D_ = +68 (c = 0.21, MeOH); ^1^H NMR (MeOH-*d*_4_) *δ* (ppm): 8.44 (d, 1H, *J* = 1.2 Hz, Ar), 8.06–7.92 (m, 3H, Ar), 7.85 (dd, 1H, *J* = 1.8, 8.7 Hz, Ar), 7.71–7.58 (m, 2H, Ar), 4.29 (d, 1H, *J* = 10.0 Hz, H-2), 3.98 (dd, 1H, *J* = 1.3, 11.6 Hz, H-5), 3.78–3.65 (m, 2H, H-6a, H-6b), 3.54 (dd, 1H, *J* = 9.0, 10.0, Hz, H-3), 3.43 (t, 1H, *J* = 9.2 Hz, H-4), 1.92 (s, 3H, CH_3_). ^13^C NMR (MeOH-*d*_4_) *δ* (ppm): 173.59 (C=O), 149.28 (C=N), 137.42, 136.38, 133.51, 130.34, 130.04, 129.95, 129.92, 128.96, 128.53, 124.08 (Ar), 83.12, 74.48, 70.55 (C-3, C-4, C-5), 62.91 (C-6), 53.62 (C-2), 22.73 (CH_3_). ESI-HRMS positive mode (*m/z*): calcd. for C_18_H_21_N_3_O_7_S (423.1100) [M + Na]^+^ = 446.0992, found: [M + Na]^+^ = 446.0991.

##### 2-Acetamido-2-deoxy-d-glucono-1,5-lactone 1-Naphthalenesulfonylhydrazone (**5f**)

Prepared from compound **4f** (100 mg, 0.182 mmol) according to General procedure C. The crude product was purified by column chromatography to give 62 mg (80%) of white powder. m.p.: 145–147 °C; [α]_D_ = +58 (c = 0.26, MeOH); ^1^H NMR (MeOH-*d*_4_) *δ* (ppm): 8.81 (d, 1H, *J* = 8.5 Hz, Ar), 8.25 (d, 1H, *J* = 7.4 Hz, Ar), 8.16 (d, 1H, *J* = 8.2 Hz, Ar), 7.99 (d, 1H, *J* = 8.0 Hz, Ar), 7.72–7.55 (m, 3H, Ar), 4.25 (d, 1H, *J* = 9.8 Hz, H-2), 4.00 (dd, 1H, *J* = 5.2, 9.9 Hz, H-5), 3.75–3.66 (m, 2H, H-6a, H-6b), 3.47 (dd, 1H, *J* = 8.9, 9.9, Hz, H-3), 3.40 (t, 1H, *J* = 8.9 Hz, H-4), 1.90 (s, 3H, CH_3_). ^13^C NMR (MeOH-*d*_4_) *δ* (ppm): 173.48 (C=O), 148.44 (C=N), 135.76, 135.66, 135.59, 131.46, 129.91, 129.84, 128.96, 127.84, 126.23, 125.23 (Ar), 83.27, 74.70, 70.62 (C-3, C-4, C-5), 63.08 (C-6), 53.46 (C-2), 22.70 (CH_3_). ESI-HRMS positive mode (*m/z*): calcd. for C_18_H_21_N_3_O_7_S (423.1100) [M + Na]^+^ = 446.0992, found: [M + Na]^+^ = 446.0992.

### 3.2. Biochemical Materials and Methods

#### 3.2.1. Protein Expression and Purification

The detailed protocol for the expression of hOGA and the subsequent purification procedure of the recombinant protein have been described in our previous work [12]. The gene construct encoding the full-length human OGA fused to N-terminal His_6_-tag was kindly provided by Prof. D. Vocadlo (SFU, Burnaby, BC, Canada). Human β-*N*-acetylhexosaminidase B (hHexB) was expressed in *Pichia pastoris*, and the extracellularly produced enzyme was isolated according to the procedure described by Krejzová et al. [20].

#### 3.2.2. Inhibition Studies

The inhibitory potency of compounds **5a–f** was evaluated against recombinant enzymes hOGA and hHexB. In the case of hOGA inhibition, 4-MU-GlcNAc was used as a substrate at three concentrations (7 µM, 12 µM, and 25 µM) in the reaction mixtures containing 50 mM potassium phosphate buffer (pH 7.5), an inhibitor **5a–f** in a final concentration of 0 to 750 nM, and 3 nM hOGA. In all cases, the enzyme and the inhibitors were preincubated at 37 °C, then the reaction was initiated by the addition of the substrate. All reactions were carried out in a fluorescence cuvette (Hellma Analytics, Müllheim, Germany), held in the thermostat cell holder of a Jasco FP-8200 fluorescence spectrophotometer (Easton, MD, USA) equipped with a Xe lamp light source. The excitation of the reactions was performed at 360 nM and emission was detected at 450 nM every 10 s for 10 min. All measurements were performed in triplicates. Initial velocities were determined and the reciprocal of initial velocities was plotted against inhibitor concentrations. The inhibitory mode of the compounds on hOGA was assessed by the Cornish–Bowden method, which involves the plotting of [S]/v_0_ vs. inhibitor concentrations [27]. The linear fitting to the data points resulted in parallel lines referring to competitive inhibition. Inhibition constants (*K*_i_) were determined according to the competitive model of the Dixon method by plotting the reciprocal of initial velocities vs. inhibitor concentrations [28]. The intersection point of the linearly fitted lines gave *K*_i_ in nM.

In the case of hHexB inhibition, Michaelis–Menten kinetics were evaluated using *p*NP-GlcNAc as a substrate. The kinetic reactions took place in Eppendorf tubes in a final volume of 400 μL containing 24 nM HexB, *p*NP-GlcNAc substrate in the range of 0.1–3 mM, and various inhibitor concentrations ranging from 0 to 300 nM in 50 mM citrate-phosphate buffer, pH 5.0. The reactions were incubated at 35 °C and then 50 μL samples were taken every 60 s. Samples were mixed with a quenching solution of 150 μL 0.1 M Na_2_CO_3_ and the absorbance of the solutions was measured at 420 nm by a Tecan Sunrise plate reader (Männedorf, Switzerland). All measurements were performed in triplicates. Initial velocities were determined from the data points by linear fit, and then nonlinear regression analyses were performed using the competitive inhibition model to calculate *K*_i_ values. GraphPad Prism software was used for the calculations.

### 3.3. Computational Details

#### 3.3.1. Protein Preparation

hOGA was prepared for Prime protein–ligand refinement calculations using its co-crystallized complex with PUGNAc (PDB code 5UHO; 3.21 Å resolution) and Schrödinger’s Protein Preparation Wizard [21]. Water molecules within 5 Å of the native ligand were initially retained (deleted for subsequent calculations), bond orders were assigned and hydrogen atoms added, with the protonation states for basic/acidic residues based on PROPKA calculated p*K*_a_’s at pH 7 [29]. Subsequent optimization of hydroxyl groups, histidine sidechain C/N atom flips and protonation states, and any sidechain O/N atom flips of Gln and Asn residues was based on optimizing hydrogen-bonding networks. The system was finally minimized using OPLS-AA (2005) force field [30] under the constraint of RMSD (heavy atoms) to be maintained within 0.3 Å of the crystallographic atomic positions.

#### 3.3.2. Enzyme–Inhibitor Complex Predictions

Initial models of enzyme–inhibitor complexes for calculations were prepared by mutation of PUGNAc in chain A of the prepared crystallographic complex into the inhibitors **5a–f**. Prime v5.4 enzyme–inhibitor refinements [21] were then performed in the hierarchical optimization mode for each new complex. The default OPLSe force field [31] and the VSGB model of solvation [32] were employed. Residues within 5 Å of PUGNAc in 5UHO were free in all refinement calculations (same 360 atoms), but with hydrogen bond constraints (force constant 100 kcal mol^−1^ Å^−2^) to the *N*-acetyl-glucosamine moiety to reflect the solved hOGA-PUGNAc crystallographic data; specifically (cf. Table 4), Asp285 carboxylate–inhibitor O4′ and O6′ hydrogens, Gly67 backbone O–ligand O3′ hydrogen, Asp174 and Asp175 sidechain carboxylates–ligand N2′ hydrogen, and Asn280 sidechain -NH_2_ with inhibitor acetyl carbonyl O atom. The rest of the protein atoms (beyond 5 Å) were constrained. The number of structures (enzyme–inhibitor models) to return in each case was set to 5. Initial validation of the protocol involved the application of this protocol to the cognate PUGNAc ligand for the reproduction of its solved crystallographic binding mode.

The five output complexes for each inhibitor were then further refined using two methods for comparison: additional Prime v5.4 refinements and QM/MM optimizations. For the Prime refinements, in this case, the local optimization mode was used, releasing the hydrogen bond constraints used in the initial hierarchical optimization mode calculations. The same protein residues (360 atoms) were free, with the rest of the protein constrained, as before. For the QM/MM optimizations, again the same protein residues were free and constrained as per the Prime calculations; no hydrogen bond constraints were used. In this case, the respective inhibitor was described using QM (QM region) at the M06-2X/6-31+G** level of theory [33,34,35], and the protein (MM region) was modeled using the OPLS-AA(2005) force field [30]. No cut-offs were used for non-bonded interactions. All QM/MM calculations were performed using QSite (Jaguar v10.2; Impact v81012) [21].

## 4. Conclusions

A library of 2-acetamido-2-deoxy-d-glucono-1,5-lactone arenesulfonylhydrazones was prepared from d-glucosamine in a five-step synthetic sequence in 26–44% overall yields. These compounds were assayed for their inhibitory activity against human OGA and HexB enzymes by using fluorimetric and spectrophotometric detection, respectively. Both enzymes were inhibited by the sulfonylhydrazones in the nanomolar range, and hence the compounds were considerably potent. However, the selectivity (*K*_i(hHexB)_/*K*_i(hOGA)_) varied from 1.3 to 0.21 to show no significant bias to any of the enzymes. The best inhibitor of hOGA was 2-acetamido-2-deoxy-d-glucono-1,5-lactone 1-naphthalenesulfonylhydrazone with a *K*_i_ of 27 nM and this compound had a *K*_i_ of 6.8 nM towards hHexB. The possible binding modes of the inhibitors to the hOGA enzyme were analyzed by computational methods (Prime protein–ligand refinement and QM/MM optimizations) to reveal the predicted interactions responsible for the strong binding of the compounds in a preferred *s-cis* conformation along the (C=)N-N(-H;-SO_2_) rotatable bond. This study has extended the relatively few structure–activity relationships of OGA inhibitors. However, the nonselective nature of the inhibition towards hHexB necessitates further structural modifications of the glyconolactone sulfonylhydrazone-type inhibitors.

## Figures and Tables

**Table 1:** Selected inhibitors of OGA with their activity against HexA/B enzymes and the target compounds of this study.

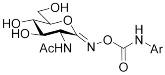 **I**			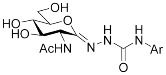 **II**		
Ar	*K*_i_ (nM)		Ar	*K*_i_ (nM)	
	OGA	HexB		OGA	HexA/B ^a^
Ph (PUGNAc)	46 [10]	36 [10]	Ph	190 [12]	205 [12]
4-Me-C_6_H_4_-	28 [9]	21 [9]	4-Me-C_6_H_4_-	155 [12]	332 [12]
4-Br-C_6_H_4_-	56 [9]	47 [9]	4-Cl-C_6_H_4_-	83 [12]	170 [12]
			2-Naphthyl	36 [12]	47 [12]
Target compounds			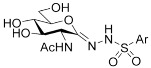		

^a^ This enzyme contains two predominant isozymes, Hex A, a heterodimer, and Hex B, a homodimer.

**Table 2:** Synthesis of test compounds **5**.

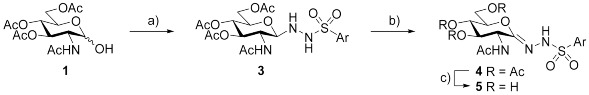					
Reagents and conditions: (a) ArSO_2_NHNH_2_ (**2**, 1.5 equiv.), *p*-TsOH·H_2_O (0.1 equiv.), CHCl_3_, reflux; (b) activated MnO_2_, abs. CH_2_Cl_2_, reflux; (c) NH_3_/MeOH, r.t.					
	Ar	Products and yields (%)			
		**3**	**4**	**5**	Overall yield of **5** from d-glucosamine
**a**	*p*-MePh	77	69	94	41
**b**	*p*-CF_3_Ph	85	60	83	35
**c**	*p*-FPh	86	72	85	44
**d**	*p*-ClPh	89	77	78	44
**e**	2-naphthyl	76	56	75	26
**f**	1-naphthyl	89	66	80	39

**Table 3:** Binding affinities of compounds **5** toward hOGA and hHexB enzymes compared to those of some previously known inhibitors.

Compound		*K*_i_ (nM)		*K*_i(hHexB)_/ *K*_i(hOGA)_^a^
		hOGA	hHexB	
PUGNAc	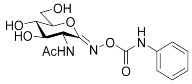	46 [10]	36 [10]	0.8
2-acetamido-2-deoxy- d-glucono-1,5-lactone 4-(2-naphthyl)-semicarbazone	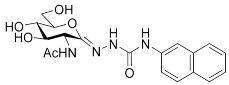	36 [12]	47 [12]	1.3
**5a**	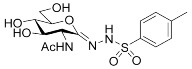	78 ± 1	21 ± 2	0.27
**5b**	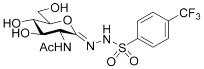	230 ± 17	48 ± 4	0.21
**5c**	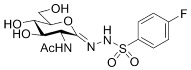	95 ± 11	45 ± 3	0.43
**5d**	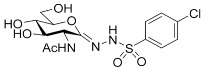	70 ± 3	39 ± 2	0.56
**5e**	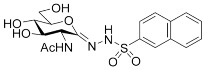	30 ± 2	30 ± 3	1
**5f**	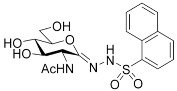	27 ± 7	6.8 ± 1.8	0.25

^a^ The ratio defines selectivity towards hOGA.

**Table 4:** Energy results for the predicted binding poses of PUGNAc and the six inhibitors **5a**–**f** with hOGA. The atom numbering scheme and the two key potential binding conformations (*s-trans* and *s-cis*) around the N1–N2 bond are highlighted.

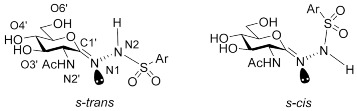							
Inhibitor/pose	Prime Hierarchical		Prime Local		QM/MM		
	Prime Energy (kcal/mol)	Relative Energy (kcal/mol)	Prime Energy (kcal/mol)	Relative Energy (kcal/mol)	Absolute Energy (Hartrees)	Relative Energy (kcal/mol)	Dihedral ^a^ (°) C1′=N1-N2-H
PUGNAc							
Pose 1	−34,316.1	0.0	−34,320.1	0.6	−1328.97031	1.2	-
Pose 2	−34,315.9	0.2	−34,320.7	0.0	−1328.96987	1.5	-
Pose 3	−34,315.8	0.3	−34,320.1	0.6	−1328.97033	1.2	-
Pose 4	−34,315.6	0.5	−34,319.9	0.8	−1328.97226	0.0	-
Pose 5	−34,315.6	0.5	−34,320.5	0.2	−1328.97227	0.0	-
**5a**							
Pose 1	−34,317.1	0.0	−34,320.7	0.9	−1728.25188	8.8	−14.9
Pose 2	−34,315.8	1.3	−34,318.0	3.6	−1728.26592	0.0	−172.2
Pose 3	−34,315.2	1.9	−34,320.2	1.4	−1728.25192	8.8	−15.1
Pose 4	−34,315.1	2.0	−34,318.6	3.0	−1728.26597	0.0	−171.8
Pose 5	−34,314.8	2.3	−34,321.6	0.0	−1728.25203	8.7	−15.1
**5b**							
Pose 1	−34,303.5	0.0	−34,310.8	0.0	−2025.91547	13.3	8.1
Pose 2	−34,302.4	1.1	−34,304.6	6.2	−2025.93662	0.0	−168.0
Pose 3	−34,302.0	1.5	−34,305.1	5.7	−2025.92286	8.6	−22.0
Pose 4	−34,301.5	2.0	−34,305.0	5.8	−2025.92249	8.9	−21.4
Pose 5	−34,301.5	2.0	−34,305.2	5.6	−2025.92290	8.6	−22.0
**5c**							
Pose 1	−34,318.0	0.0	−34,322.8	0.3	−1788.16899	8.7	−17.9
Pose 2	−34,317.5	0.5	−34,323.1	0.0	−1788.16894	8.8	−17.5
Pose 3	−34,317.4	0.6	−34,322.3	0.8	−1788.18292	0.0	−170.7
Pose 4	−34,317.3	0.7	−34,321.8	1.3	−1788.18290	0.0	−170.6
Pose 5	−34,316.8	1.2	−34,319.1	4.0	−1788.18236	0.4	−149.3
**5d**							
Pose 1	−34,318.3	0.0	−34,320.1	0.2	−2148.53075	9.0	−19.6
Pose 2	−34,316.6	1.7	−34,320.3	0.0	−2148.53084	8.9	−19.7
Pose 3	−34,315.9	2.4	−34,319.4	0.9	−2148.53121	8.7	−20.5
Pose 4	−34,315.8	2.5	−34,319.1	1.2	−2148.53073	9.0	−19.3
Pose 5	−34,315.1	3.2	−34,319.5	0.8	−2148.54505	0.0	−169.2
**5e**							
Pose 1	−34,303.0	0.0	−34,308.7	0.0	−1842.54825	8.6	−11.8
Pose 2	−34,302.9	0.1	−34,306.0	2.7	−1842.56165	0.2	−169.3
Pose 3	−34,300.8	2.2	−34,306.6	2.1	−1842.56197	0.0	−169.6
Pose 4	−34,300.6	2.4	−34,306.9	1.8	−1842.56199	0.0	−169.5
Pose 5	−34,300.3	2.7	−34,306.7	2.0	−1842.56189	0.1	−169.7
**5f**							
Pose 1	−34,308.4	0.0	−34,311.4	1.5	−1842.54544	4.7	−0.8
Pose 2	−34,304.6	3.8	−34,309.9	3.0	−1842.54384	5.7	3.6
Pose 3	−34,303.2	5.2	−34,312.9	0.0	−1842.53822	9.2	−2.1
Pose 4	−34,302.0	6.4	−34,305.5	7.4	−1842.54411	5.6	−169.9
Pose 5	−34,301.4	7.0	−34,308.7	4.2	−1842.55296	0.0	−154.1

^a^ Dihedral angles close to 0° correspond to *s-trans* and those close to ±180° to *s-cis* conformations.

## Data Availability

Not applicable.

## Supplementary Materials

The following supporting information can be downloaded at: https://www.mdpi.com/article/10.3390/ijms23031037/s1.
