# Supplementary material for: 2-Acetamido-2-deoxy-d-glucono-1,5-lactone Sulfonylhydrazones: Synthesis and Evaluation as Inhibitors of Human OGA and HexB Enzymes
Source: Int J Mol Sci. 2022 Jan 18;23(3):1037. doi: 10.3390/ijms23031037 (PMC8834866; doi:10.3390/ijms23031037)
Supplement: Supplementary file 1 [file ijms-23-01037-s001.zip › ijms-1534082-supplementary.pdf]

Supporting Information

2-Acetamido-2-deoxy-D-glucono-1,5-lactone Sulfonylhydrazones: Synthesis and Evaluation as Inhibitors of Human OGA and HexB Enzymes

Mariann Kiss,[1] István Timári,[1] Teréz Barna,[2] Zuzana Mészáros,[3] Kristýna Slámová,[3] Pavla Bojarová,[3] Vladimír Křen,[3] Joseph M. Hayes,[4] László Somsák[1*]

[1] Department of Organic Chemistry, University of Debrecen, POB 400, H-4002 Debrecen, Hungary; kiss.mariann@science.unideb.hu (M. K.); timari.istvan@science.unideb.hu (I. T.)
[2] Department of Genetics and Applied Microbiology, University of Debrecen, POB 400, H-4002 Debrecen, Hungary; barna.terez@science.unideb.hu (T. B.)
[3] Institute of Microbiology of the Czech Academy of Sciences, Laboratory of Biotransformation, Vídeňská 1083, Praha 4, CZ-142 20 Czech Republic; zuzana.meszaros@biomed.cas.cz (Z. M.); slamova@biomed.cas.cz (K. S.); bojarova@biomed.cas.cz (P. B.); kren@biomed.cas.cz (V. K.)
[4] School of Pharmacy & Biomedical Sciences, University of Central Lancashire, Preston PR1 2HE, United Kingdom; JHayes@uclan.ac.uk (J. H.)
* Correspondence: somsak.laszlo@science.unideb.hu (L. S.)

Contents

Copies of ^{1}H and ^{13}C NMR spectra 2

Figure S1 20

Dixon and Cornish-Bowden plots of hOGA inhibition data 21

Michaelis-Menten kinetic curves and Lineweaver-Burk plots of hHexB inhibition 23

Copies of ^{1}H and ^{13}C NMR spectra

^{1}H and ^{13}C NMR spectra of compound **3a**

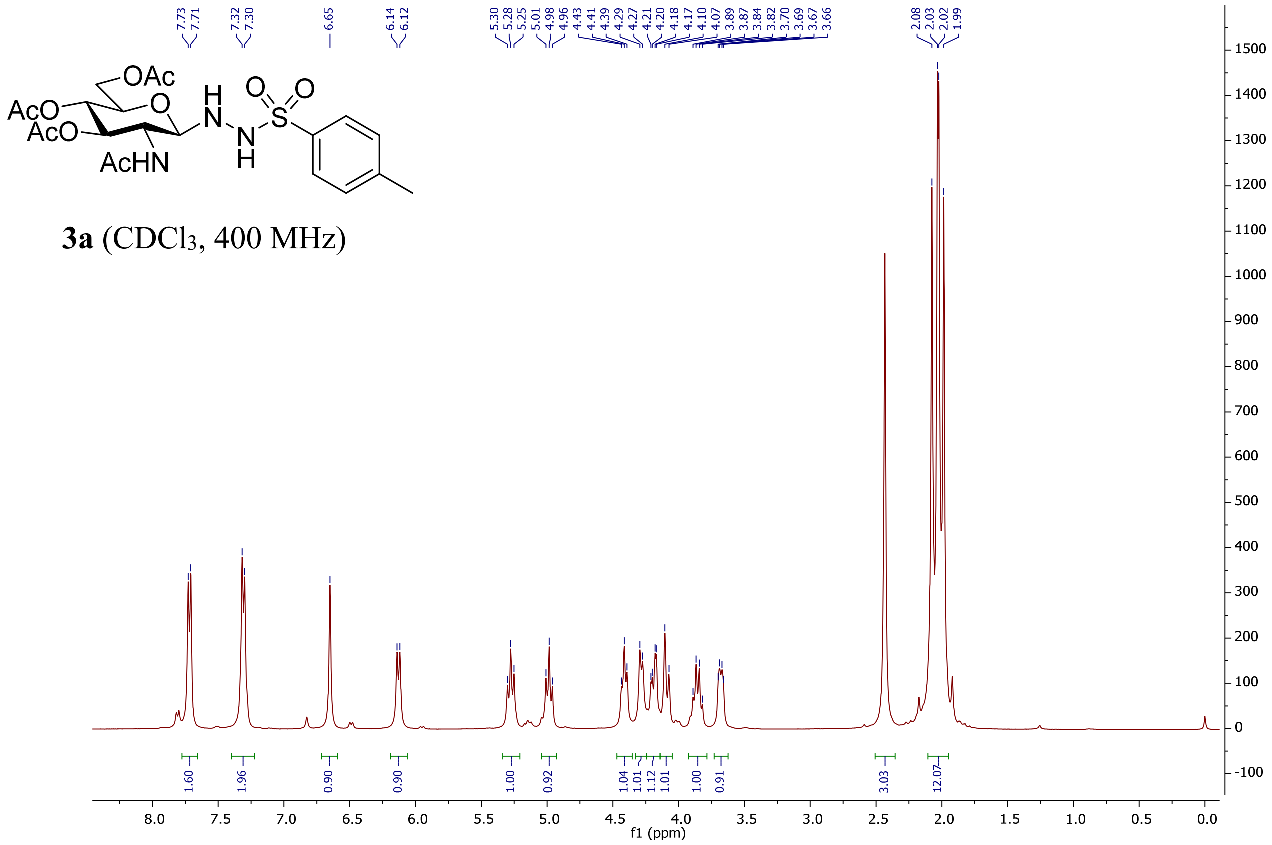

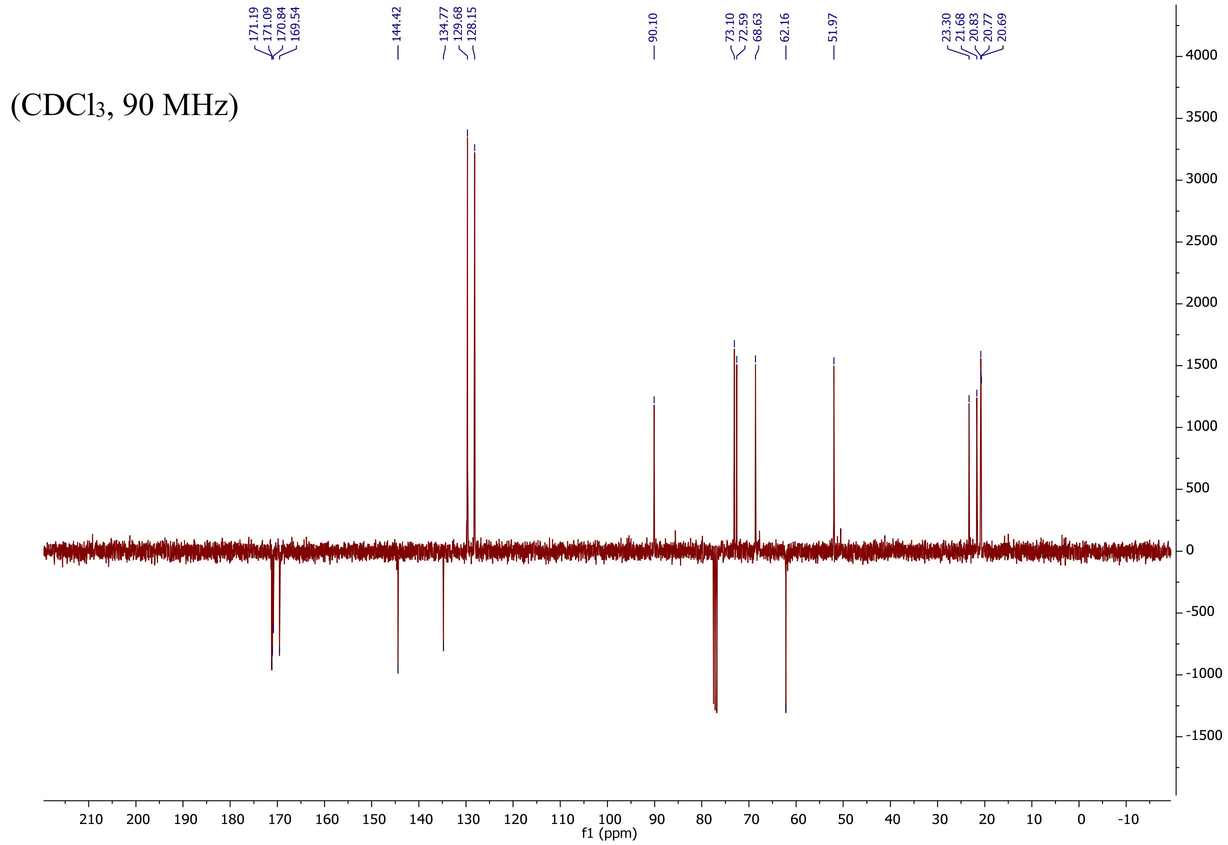

^{1}H and ^{13}C NMR spectra of compound **3b**

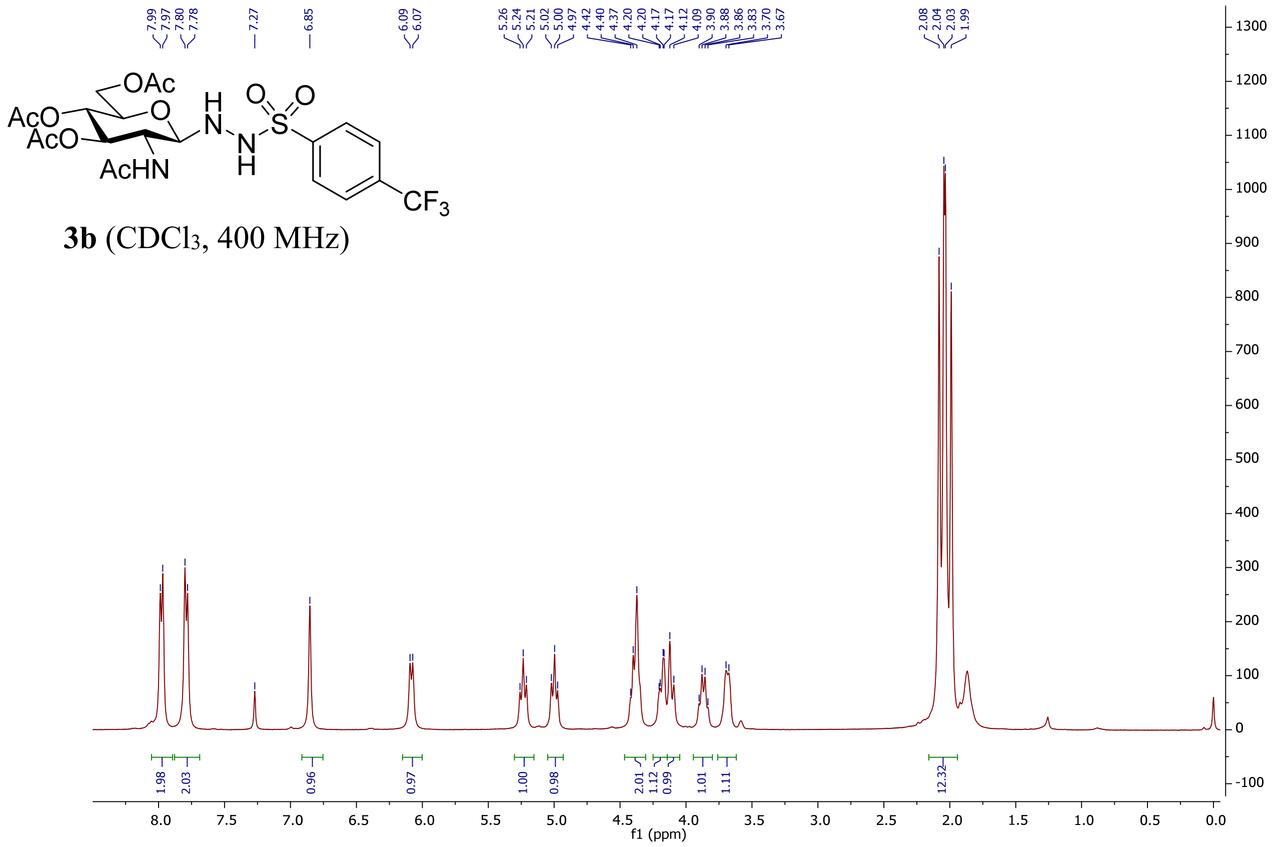

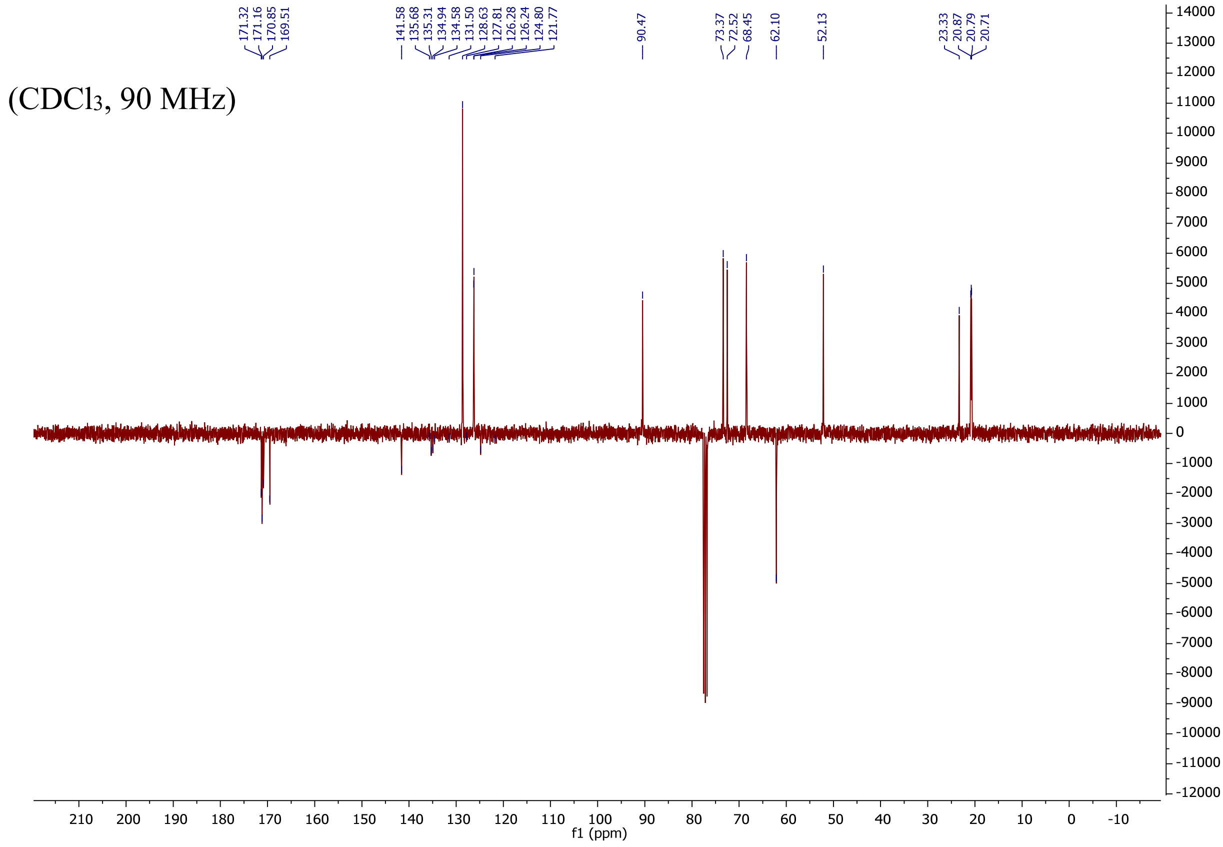

^{1}H and ^{13}C NMR spectra of compound **3c**

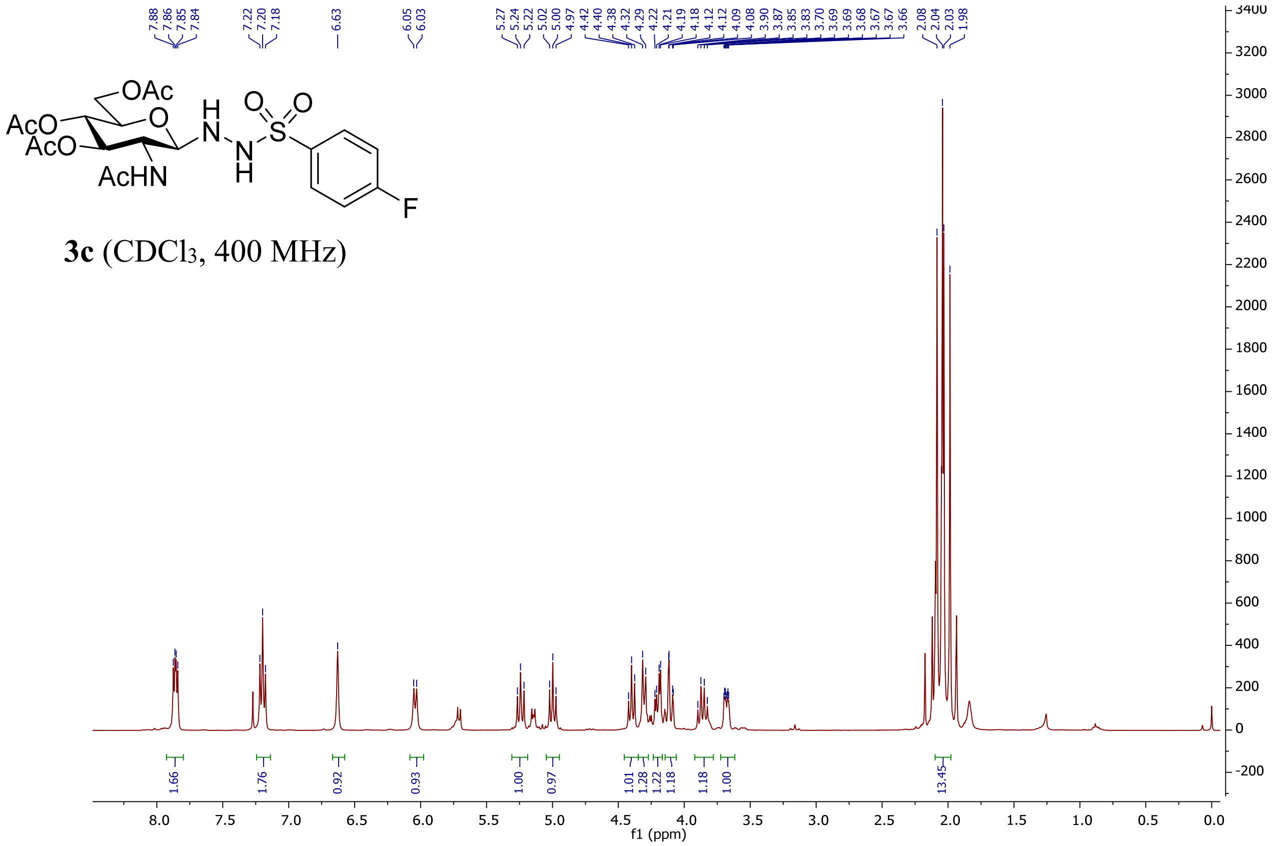

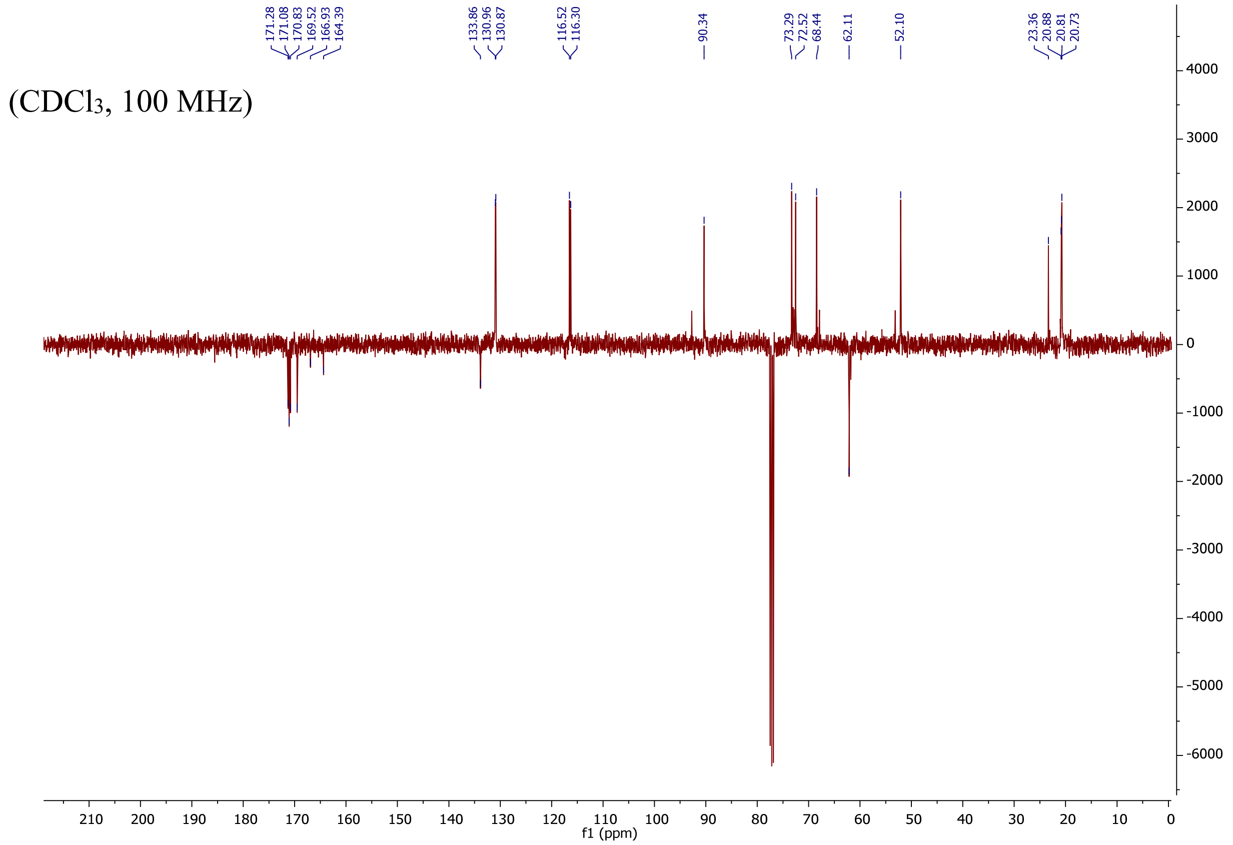

^{1}H and ^{13}C NMR spectra of compound **3d**

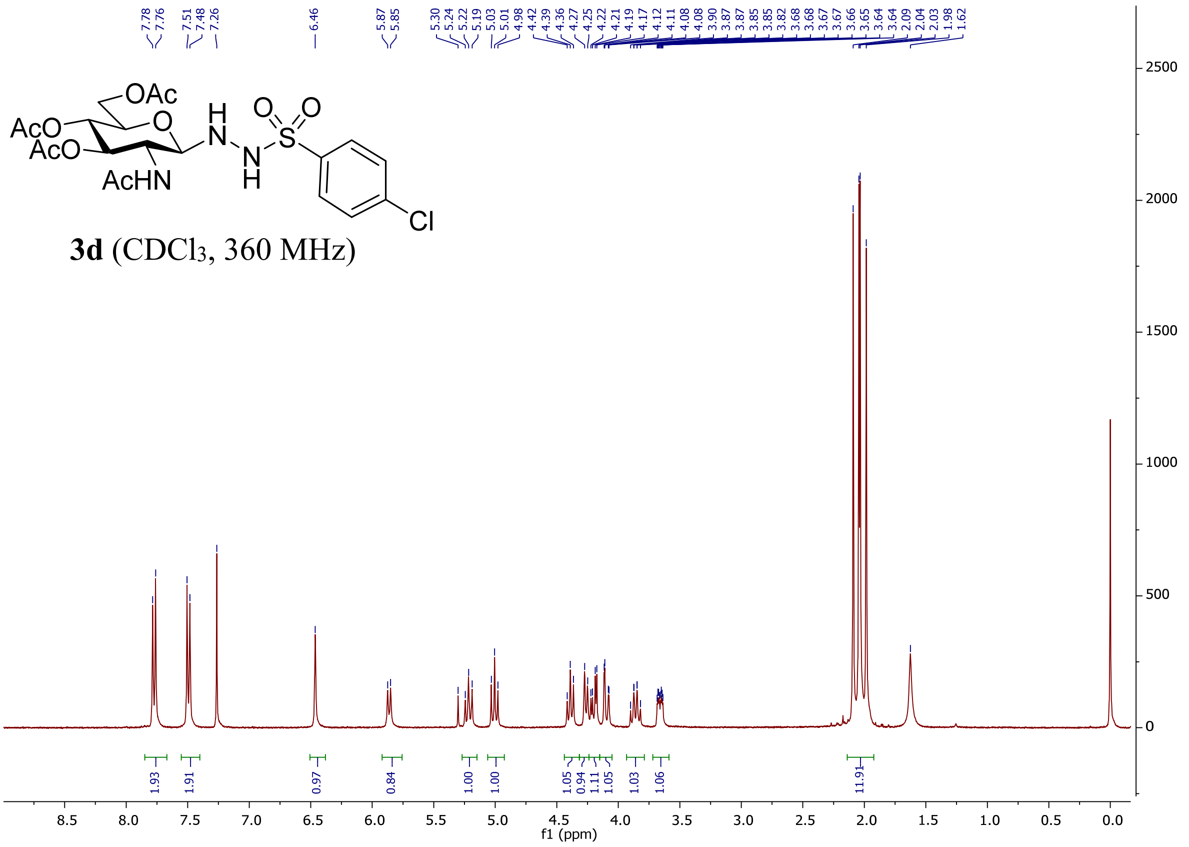

7.78
7.76
7.51
7.48
7.26
6.46
5.87
5.85
OAc
AcO
AcO
O
AcHN
H
N
N
H
O
O
S
Cl
3d (CDCl3, 360 MHz)
1.93
1.91
0.97
0.84
1.00
1.00
11.91
f1 (ppm)

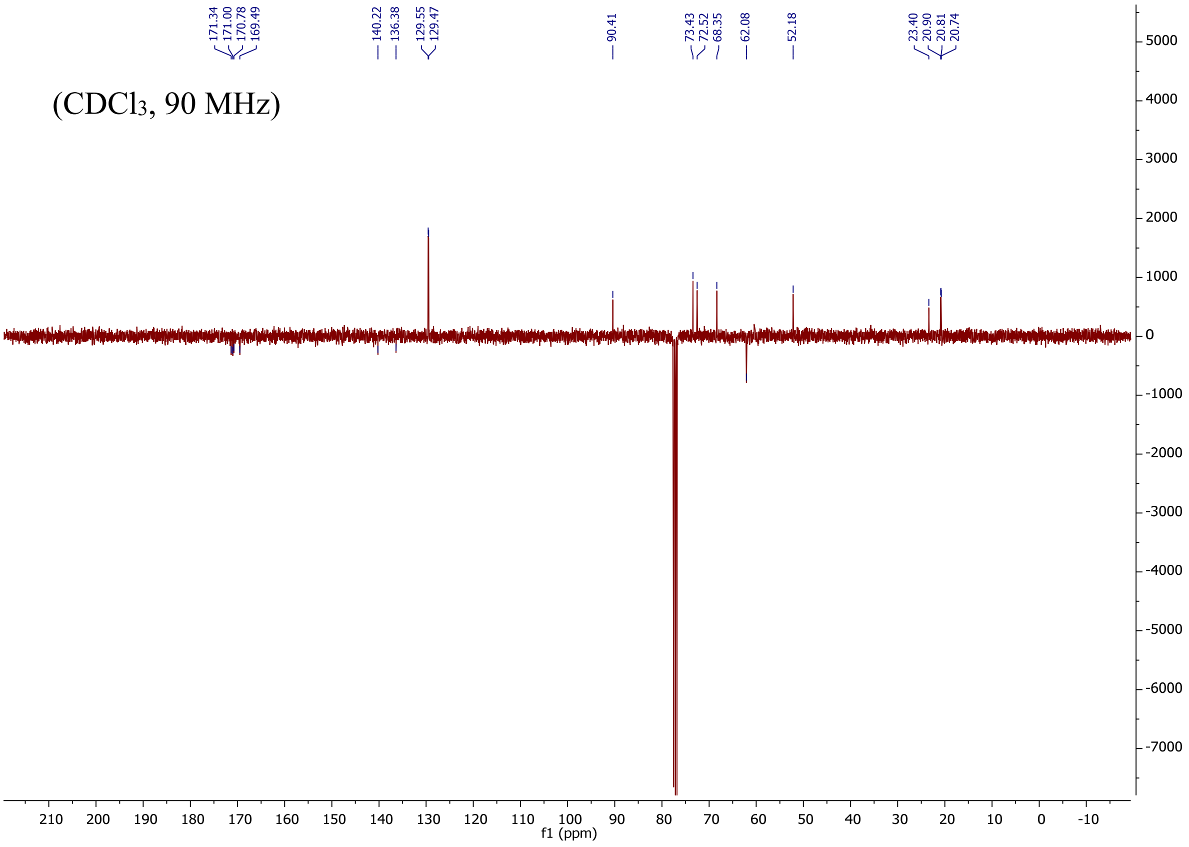

171.34
171.00
170.78
169.49
140.22
136.38
129.55
129.47
90.41
73.43
72.52
68.35
62.08
52.18
23.40
20.90
20.81
20.74
(CDCl3, 90 MHz)
f1 (ppm)

^{1}H and ^{13}C NMR spectra of compound **3e**

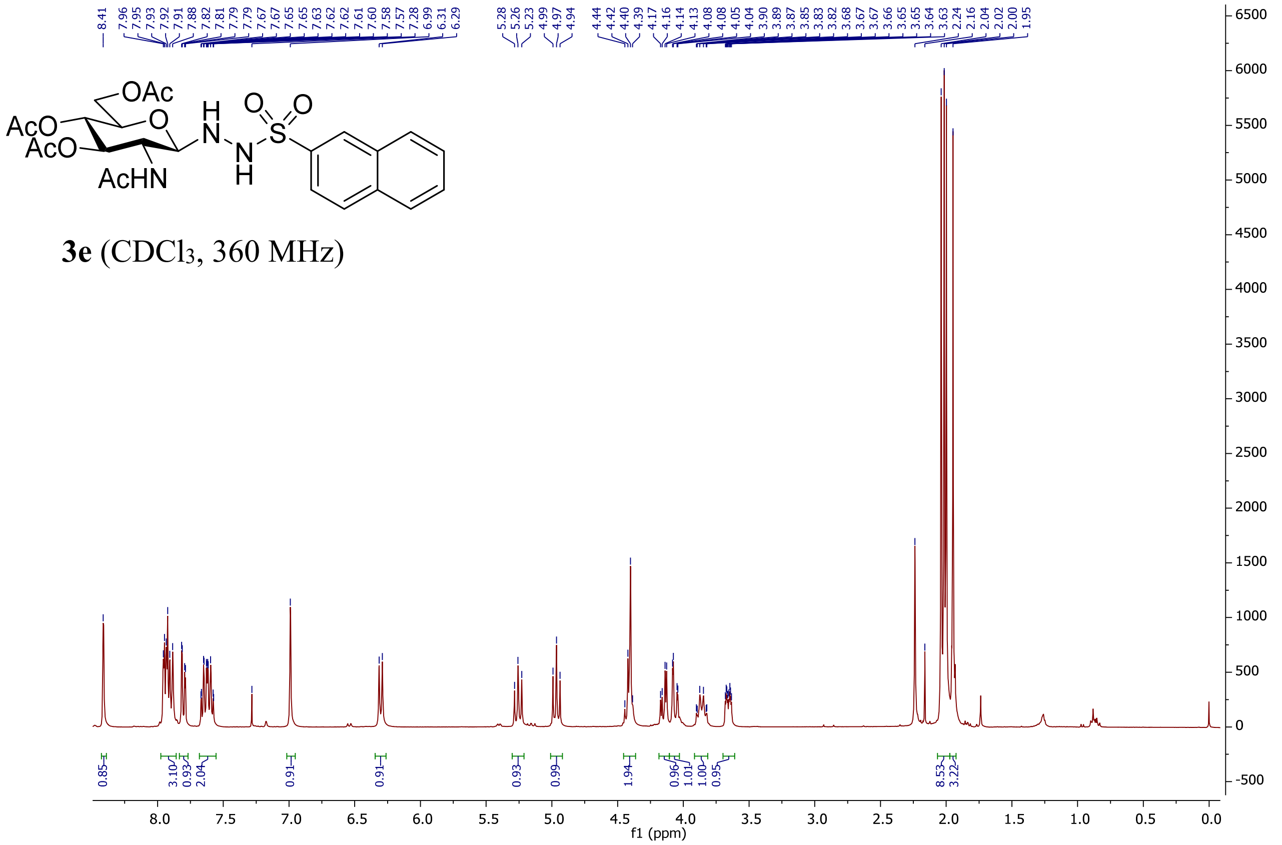

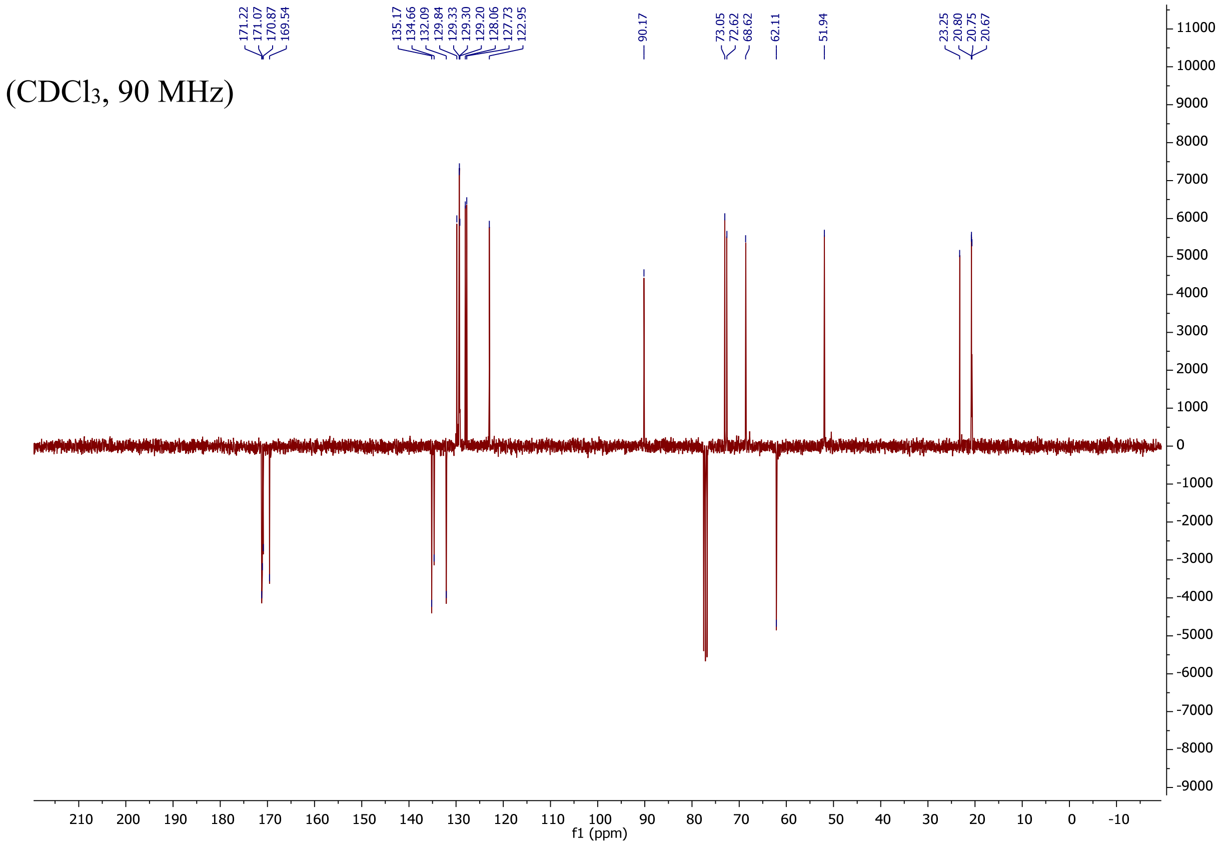

^{1}H and ^{13}C NMR spectra of compound **3f**

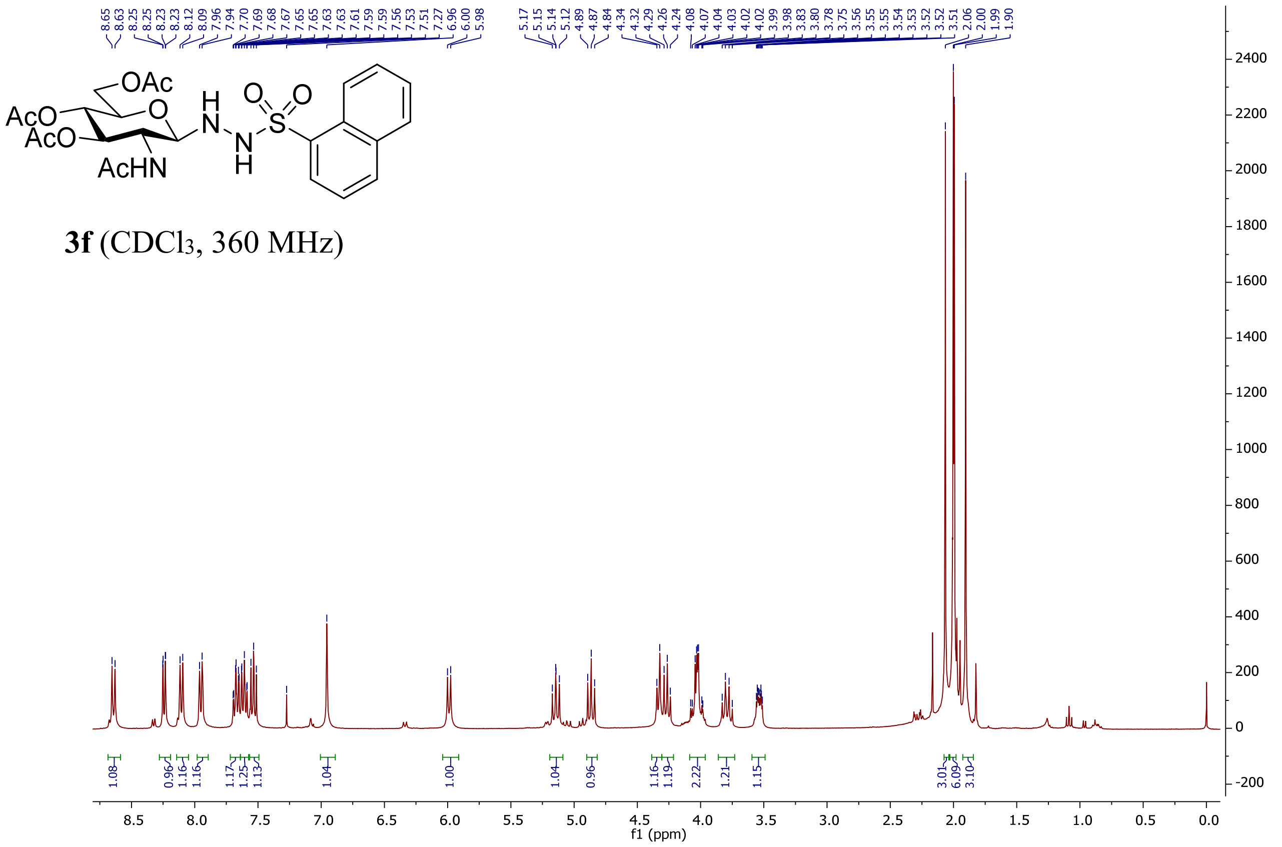

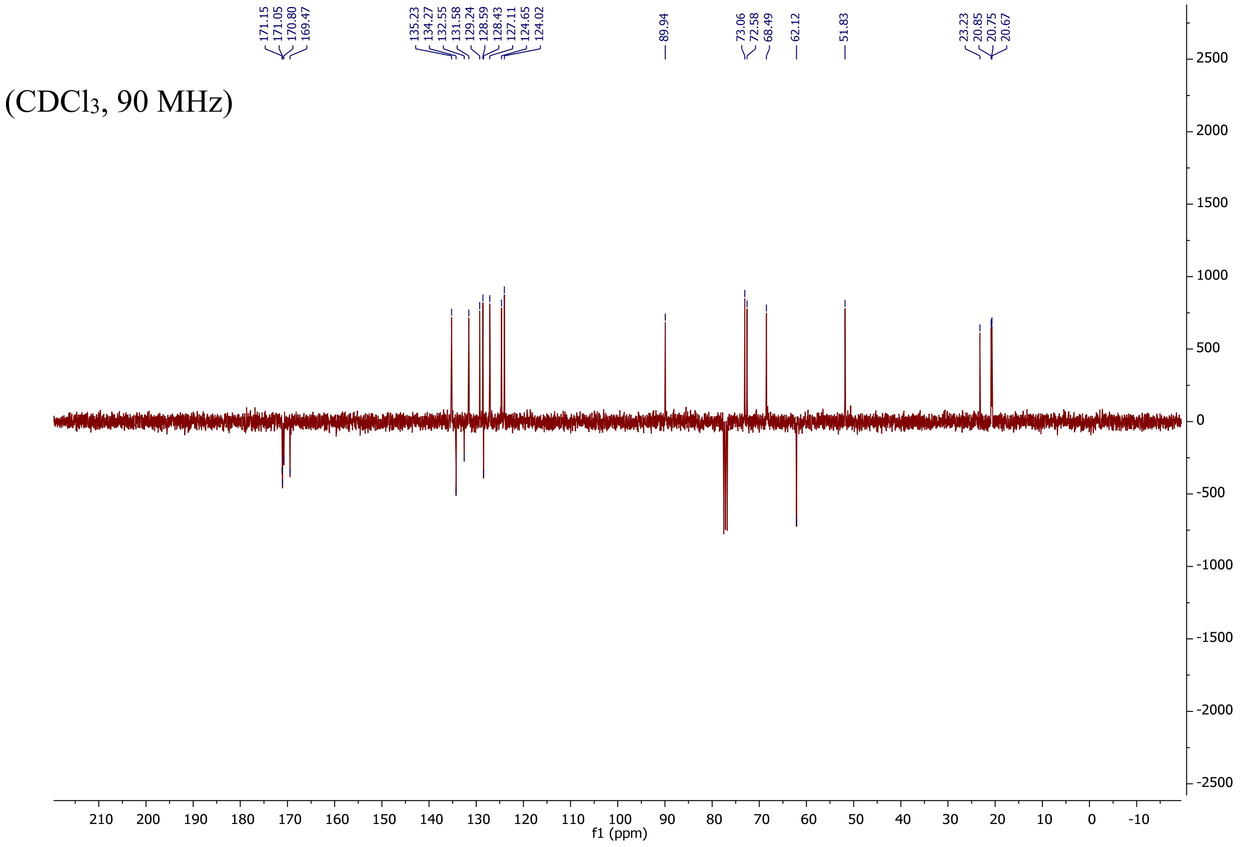

^{1}H and ^{13}C NMR spectra of compound **4a**

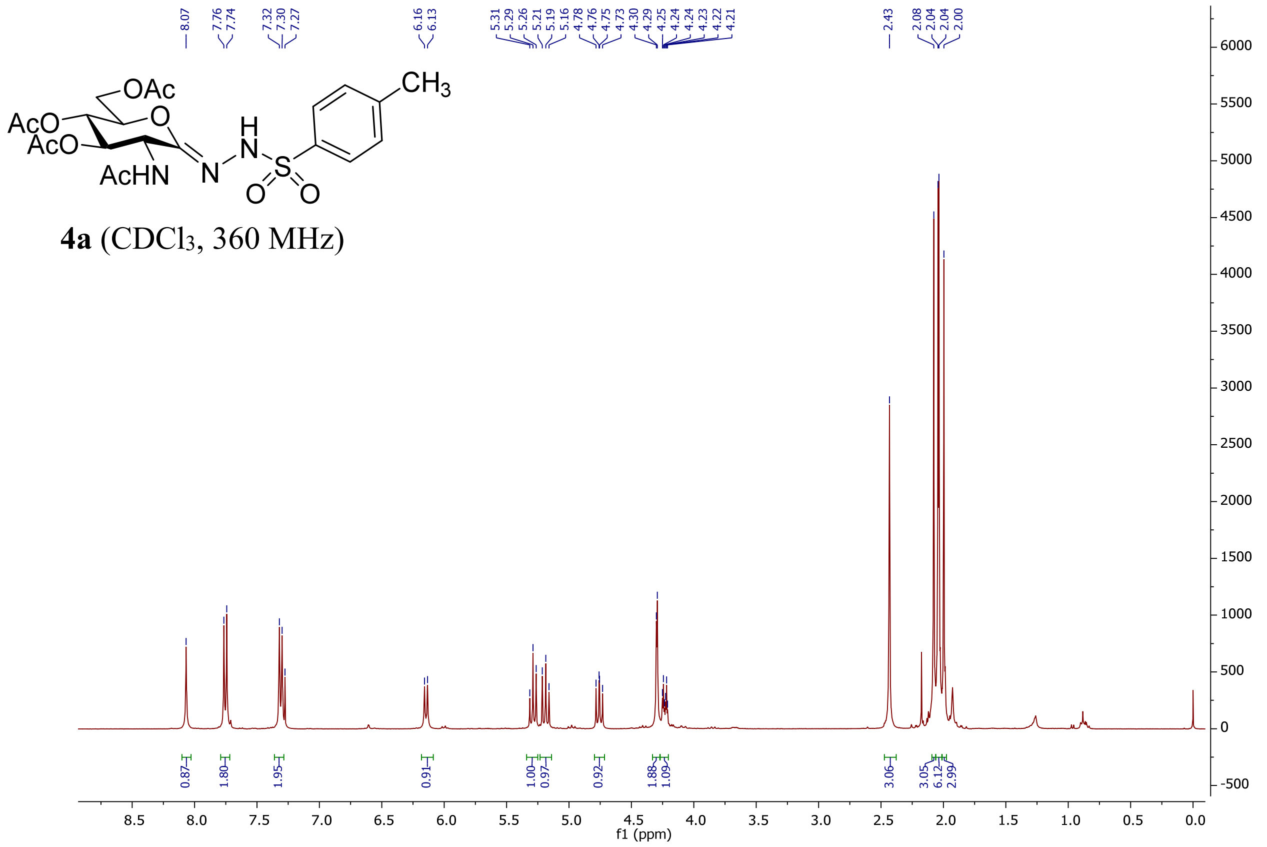

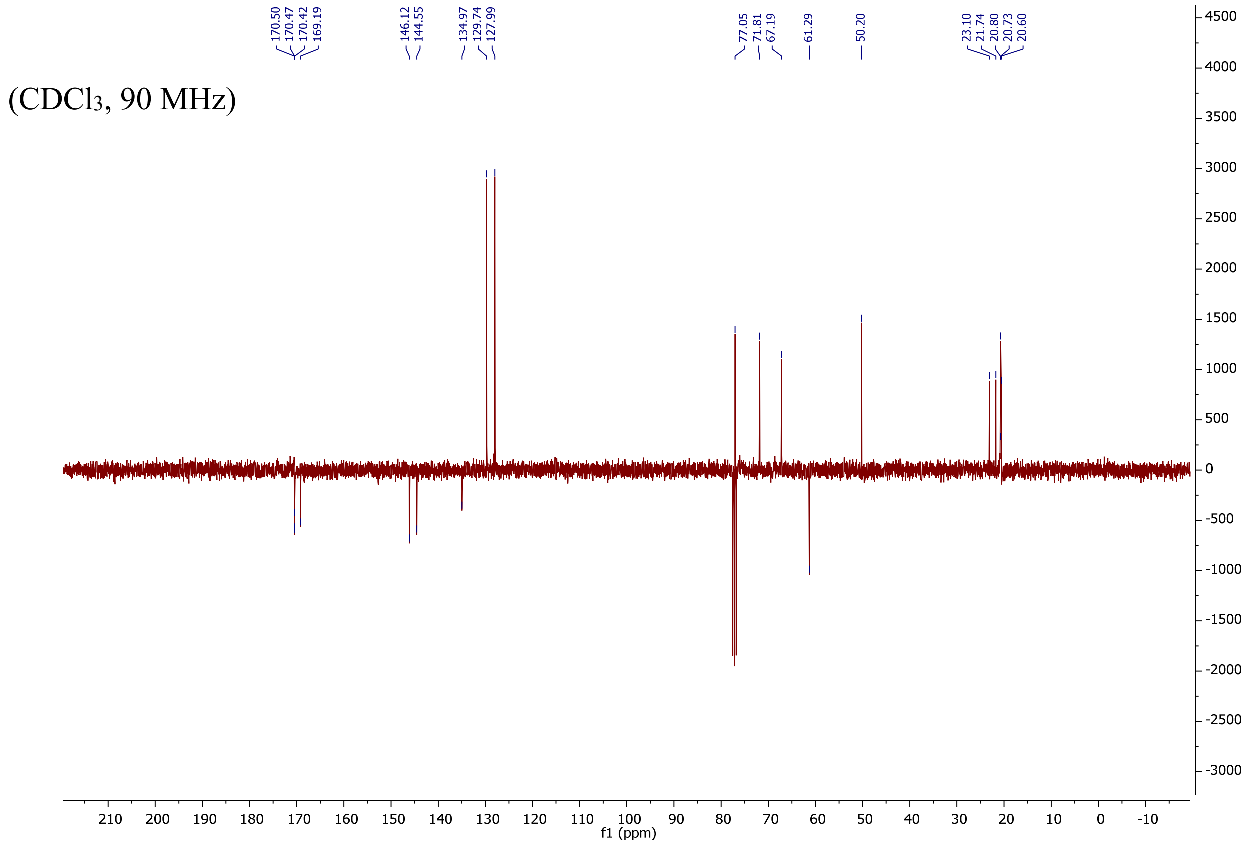

^{1}H and ^{13}C NMR spectra of compound **4b**

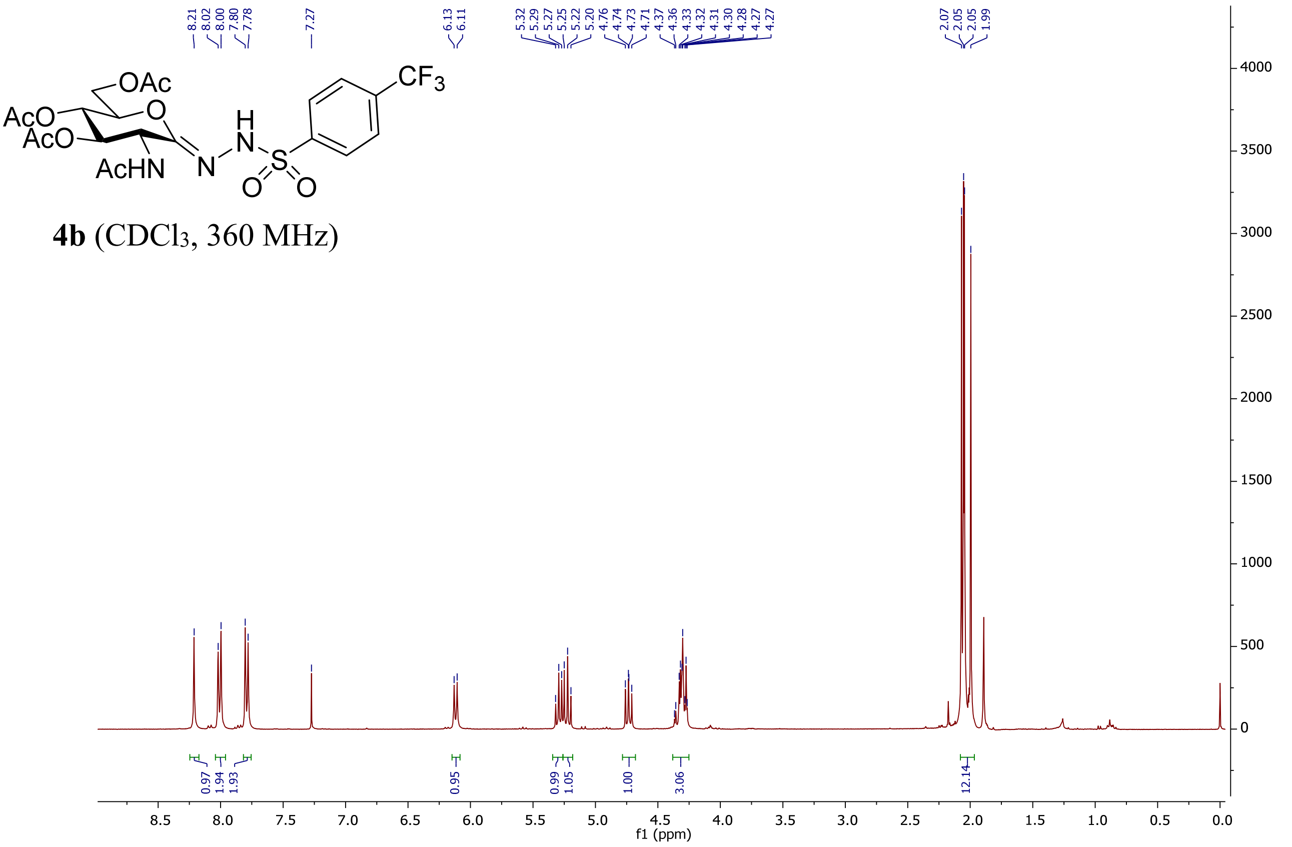

4b ($CDCl_3$, 360 MHz)

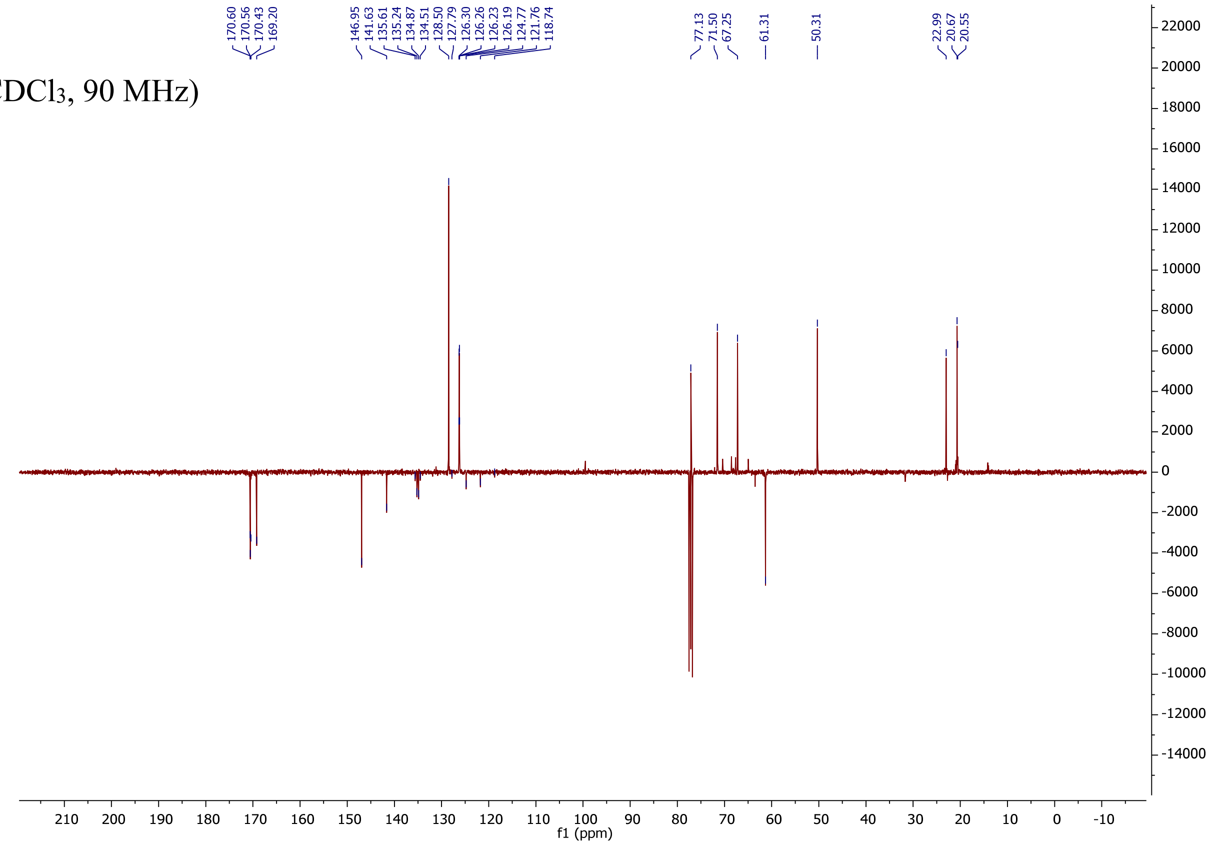

($CDCl_3$, 90 MHz)

^{1}H and ^{13}C NMR spectra of compound **4c**

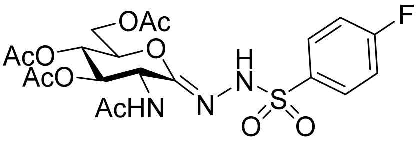

4c ($CDCl_3$, 360 MHz)

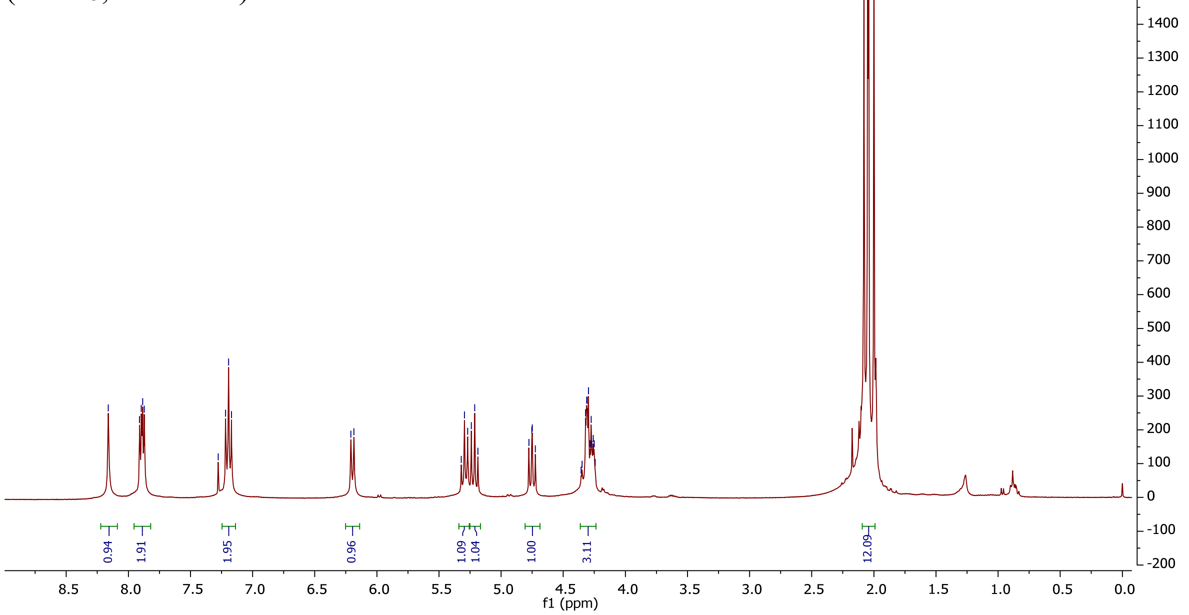

($CDCl_3$, 90 MHz)

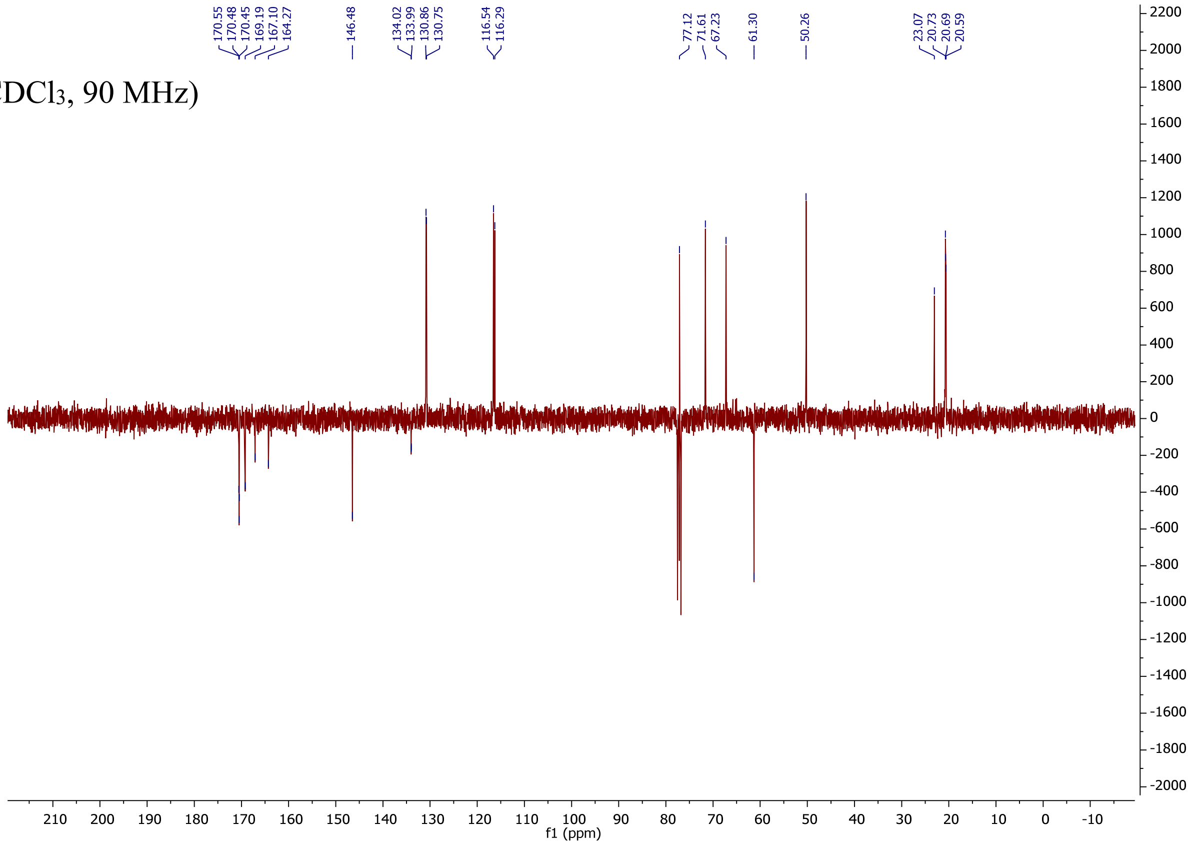

^{1}H and ^{13}C NMR spectra of compound **4d**

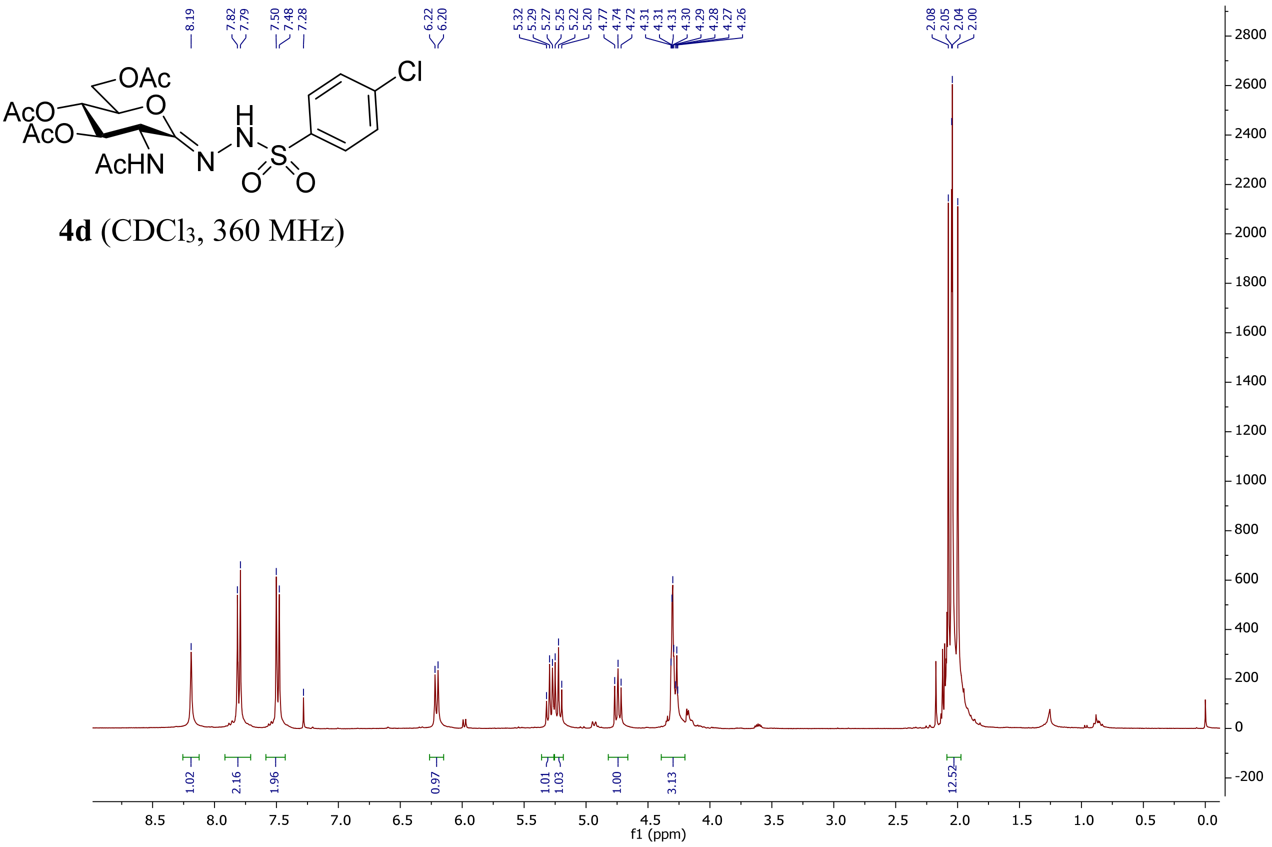

4d ($CDCl_3$, 360 MHz)

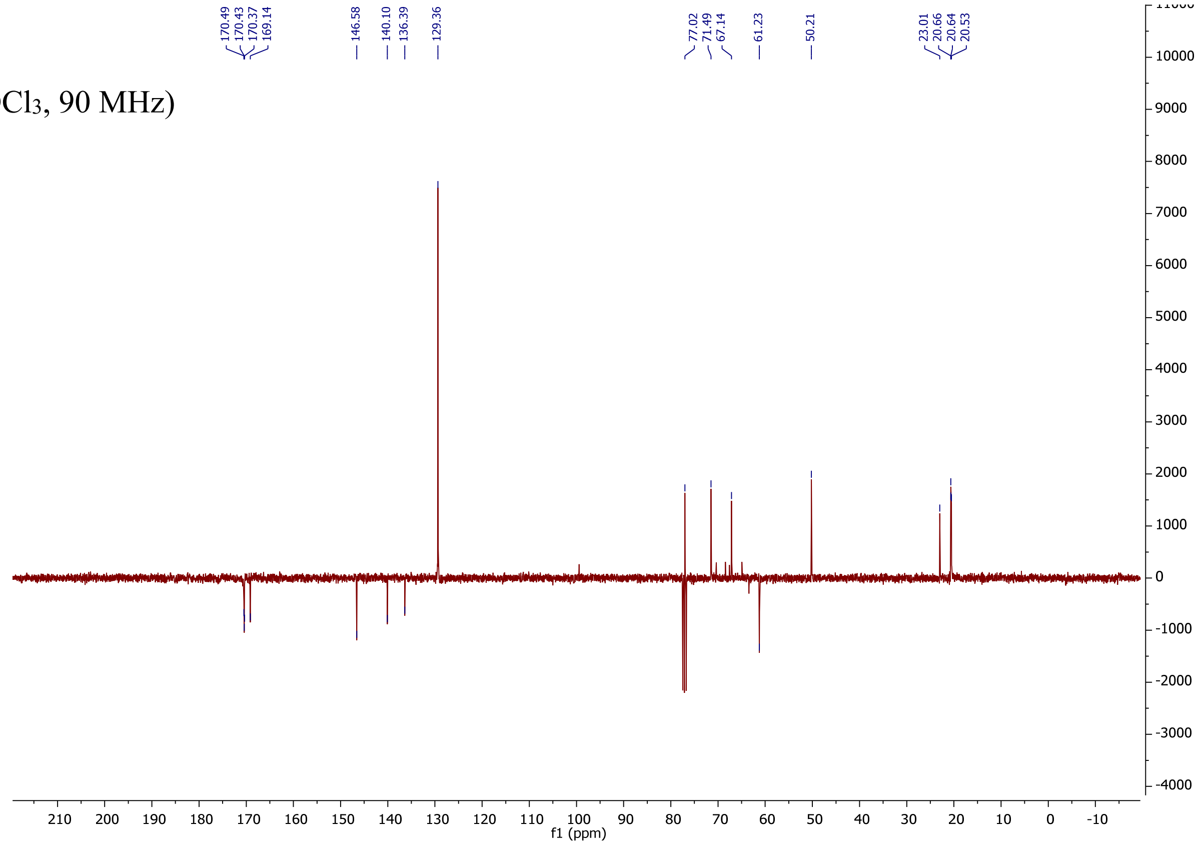

($CDCl_3$, 90 MHz)

^{1}H and ^{13}C NMR spectra of compound **4e**

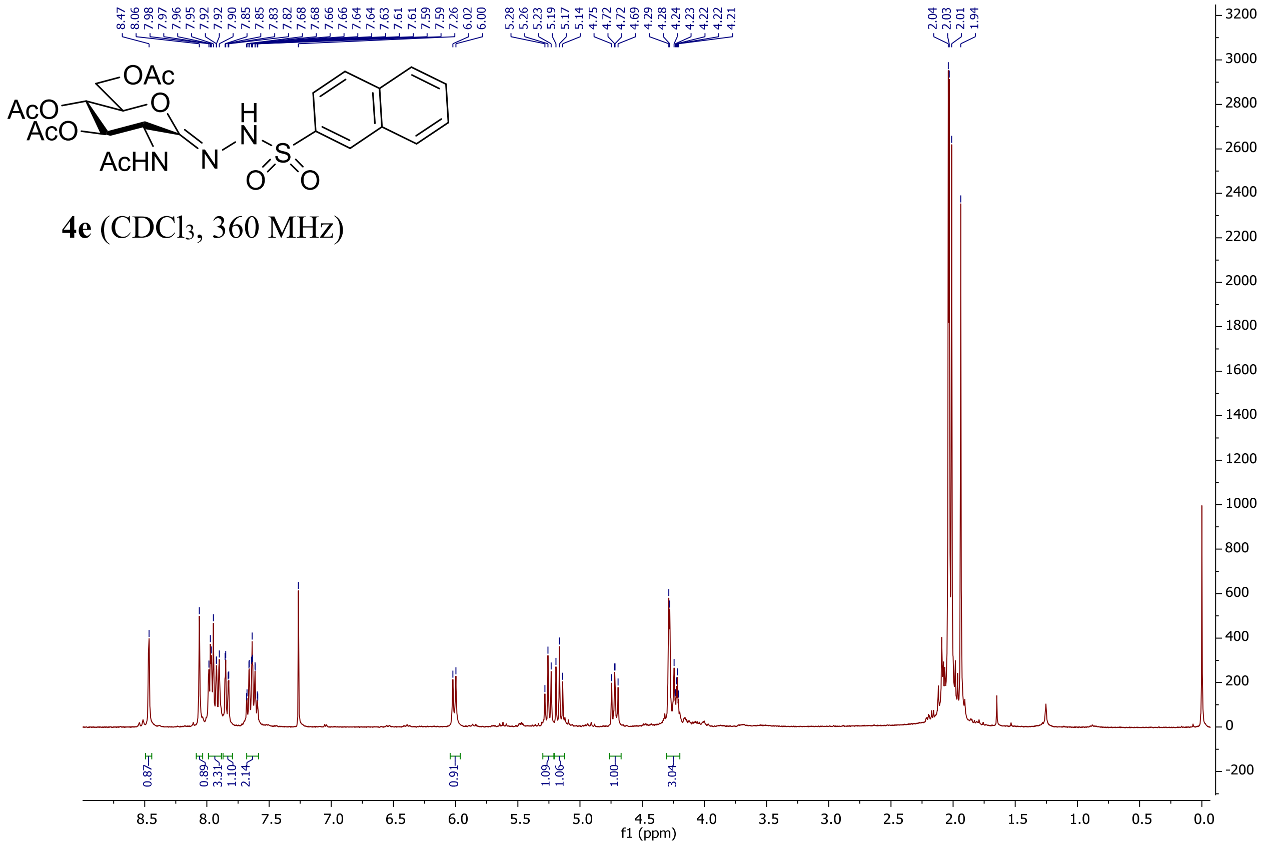

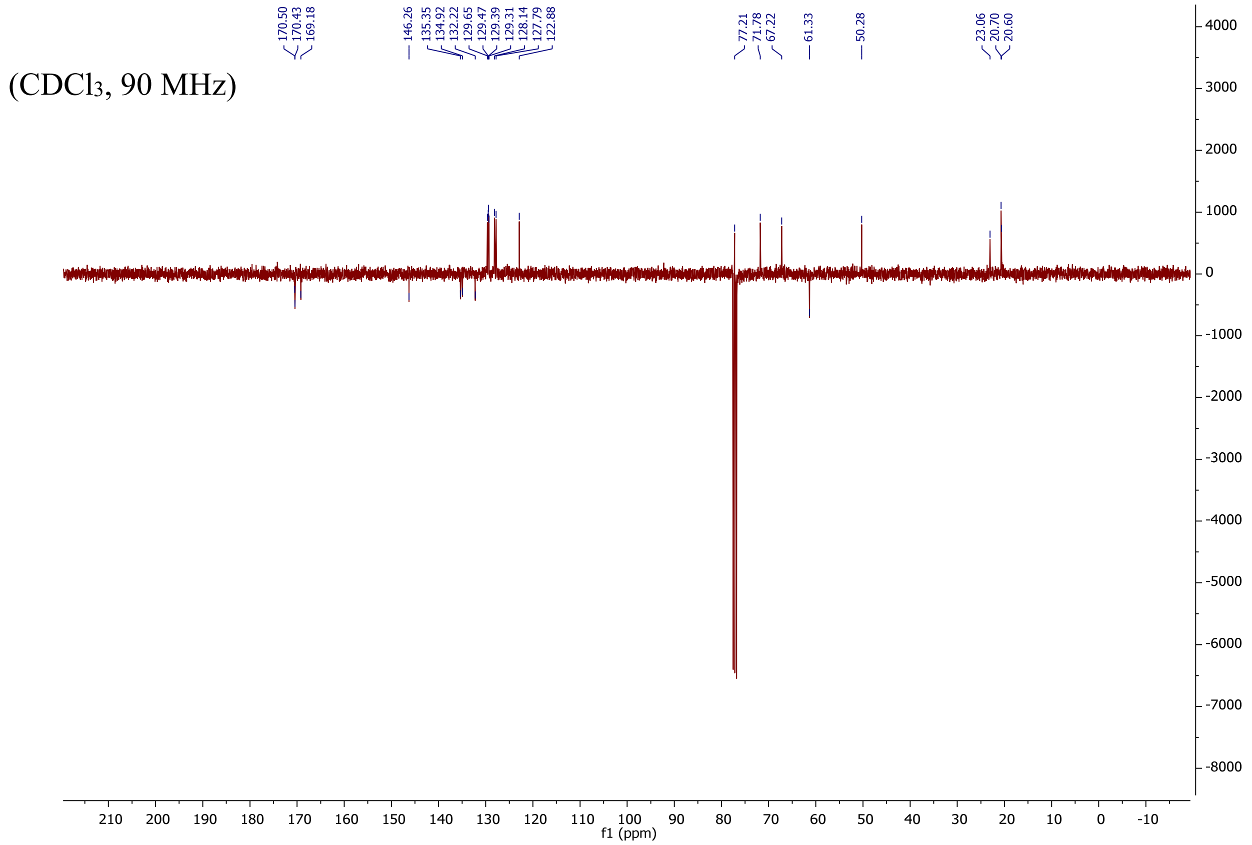

^{1}H and ^{13}C NMR spectra of compound **4f**

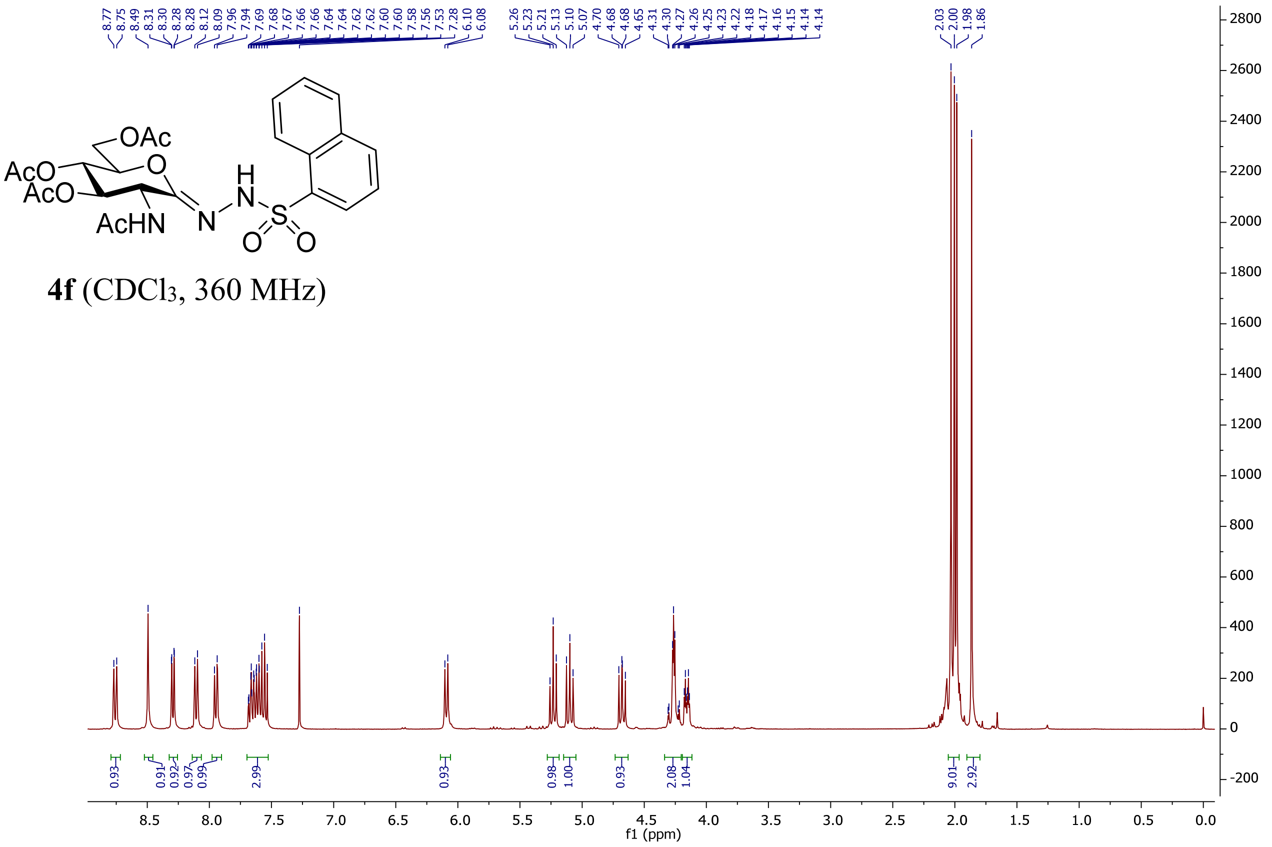

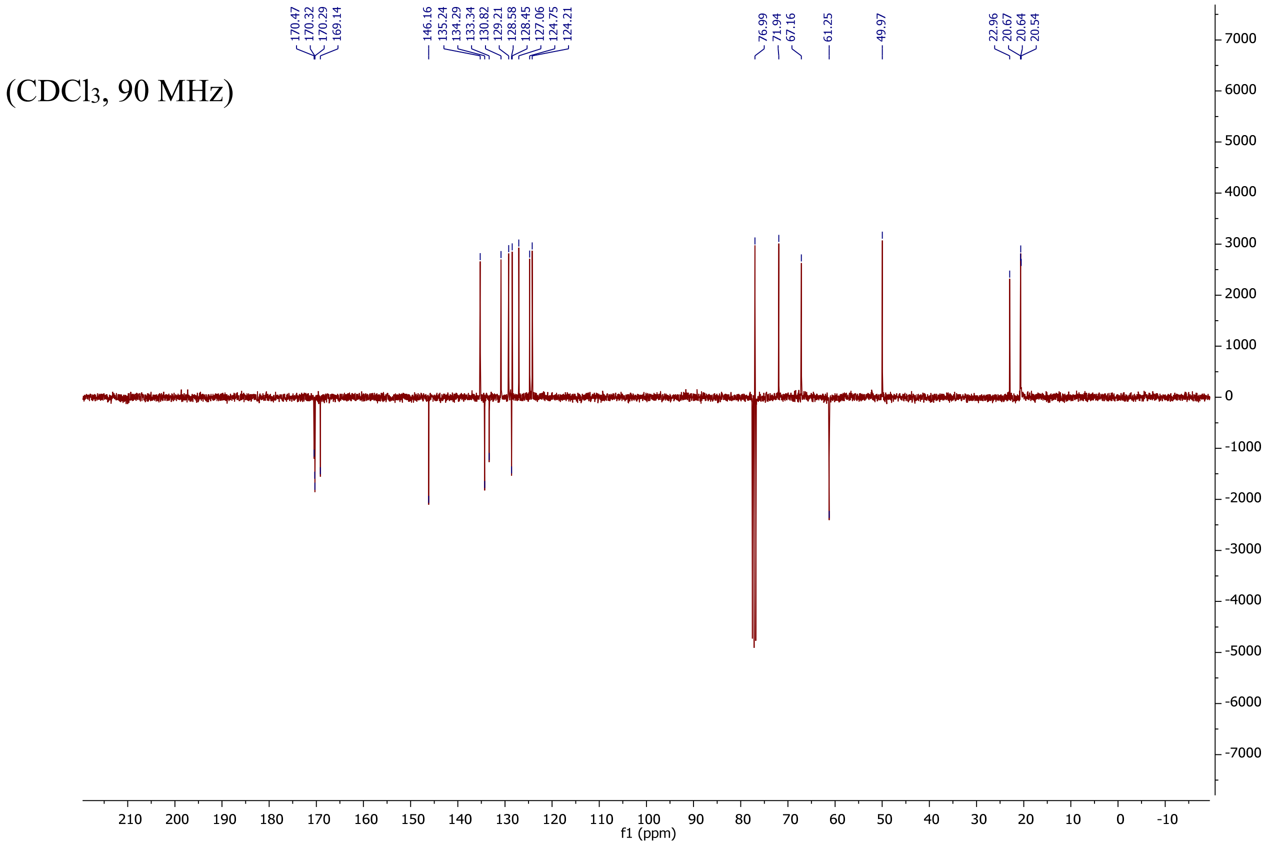

^{1}H and ^{13}C NMR spectra of compound **5a**

5a (MeOD-d4, 360 MHz)

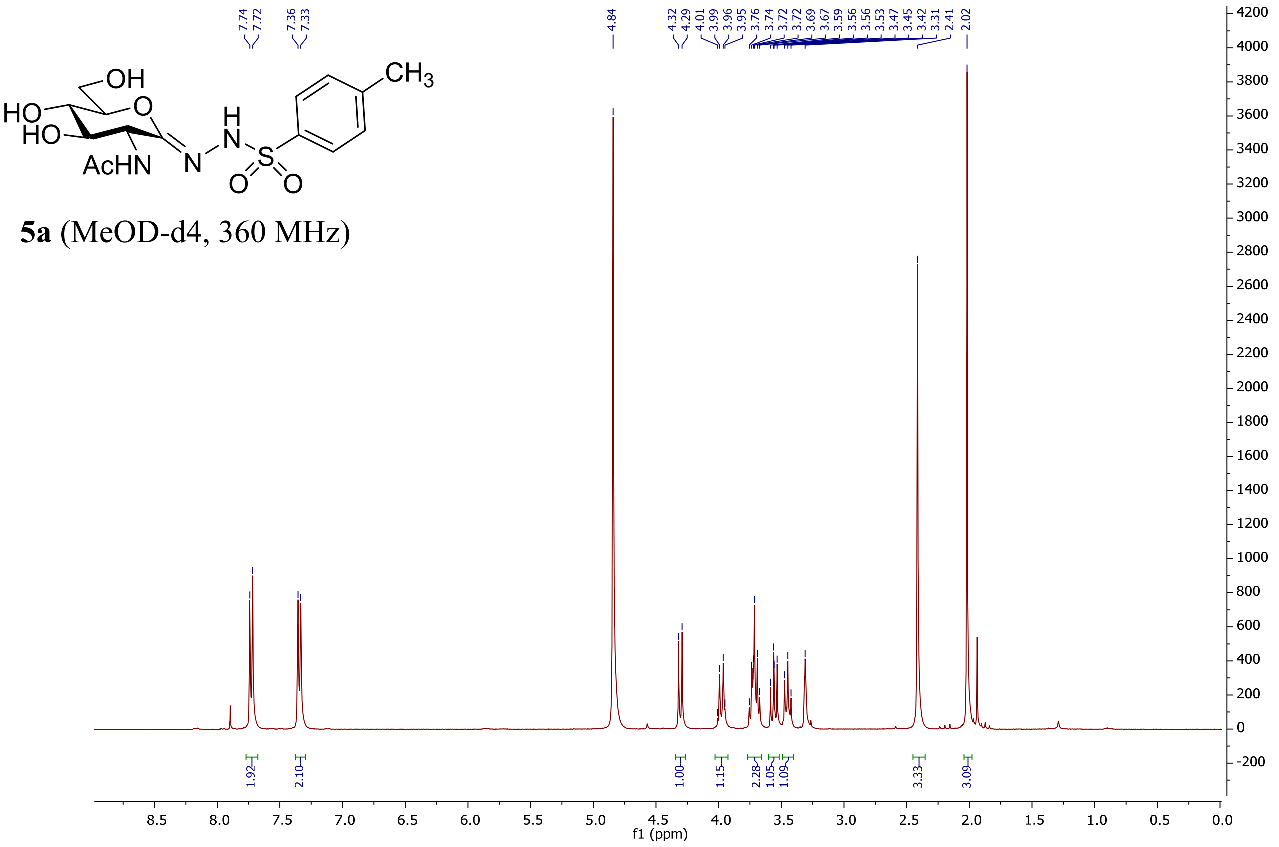

(MeOD-d4, 90 MHz)

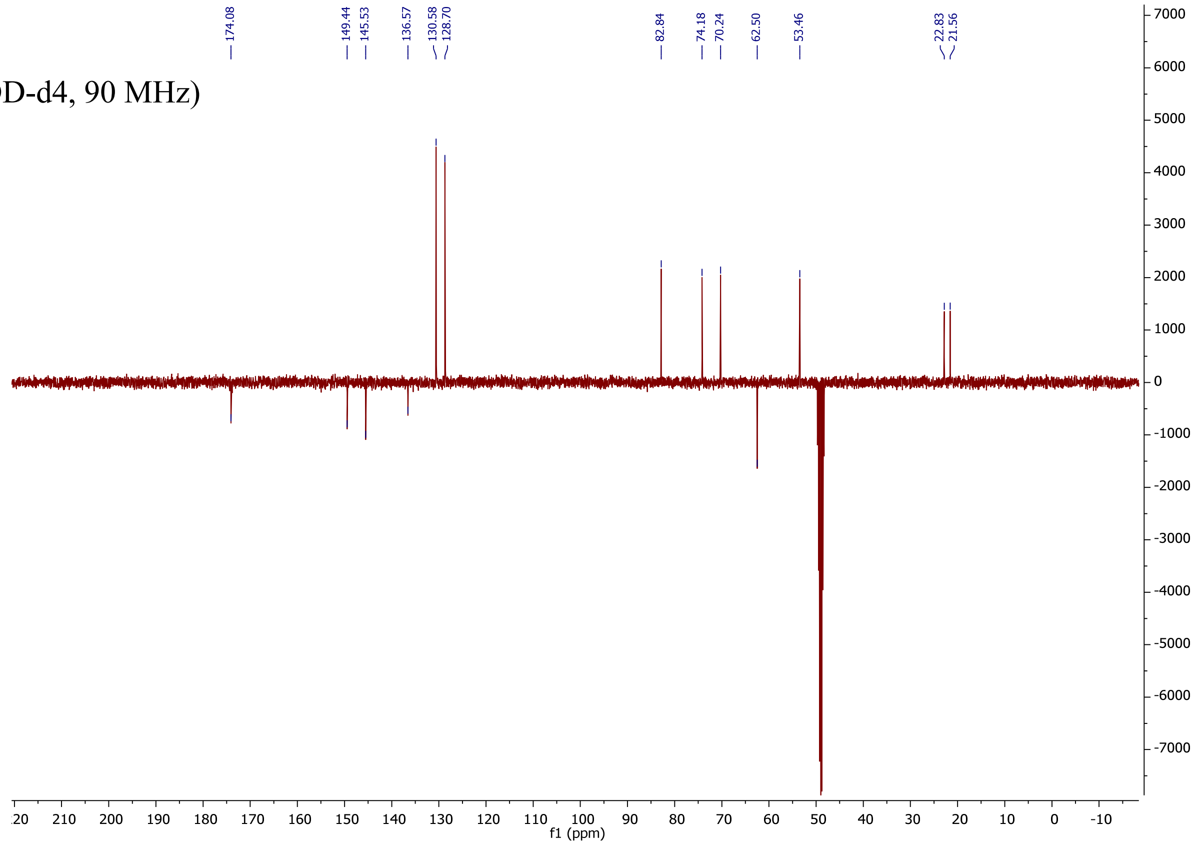

^{1}H and ^{13}C NMR spectra of compound **5b**

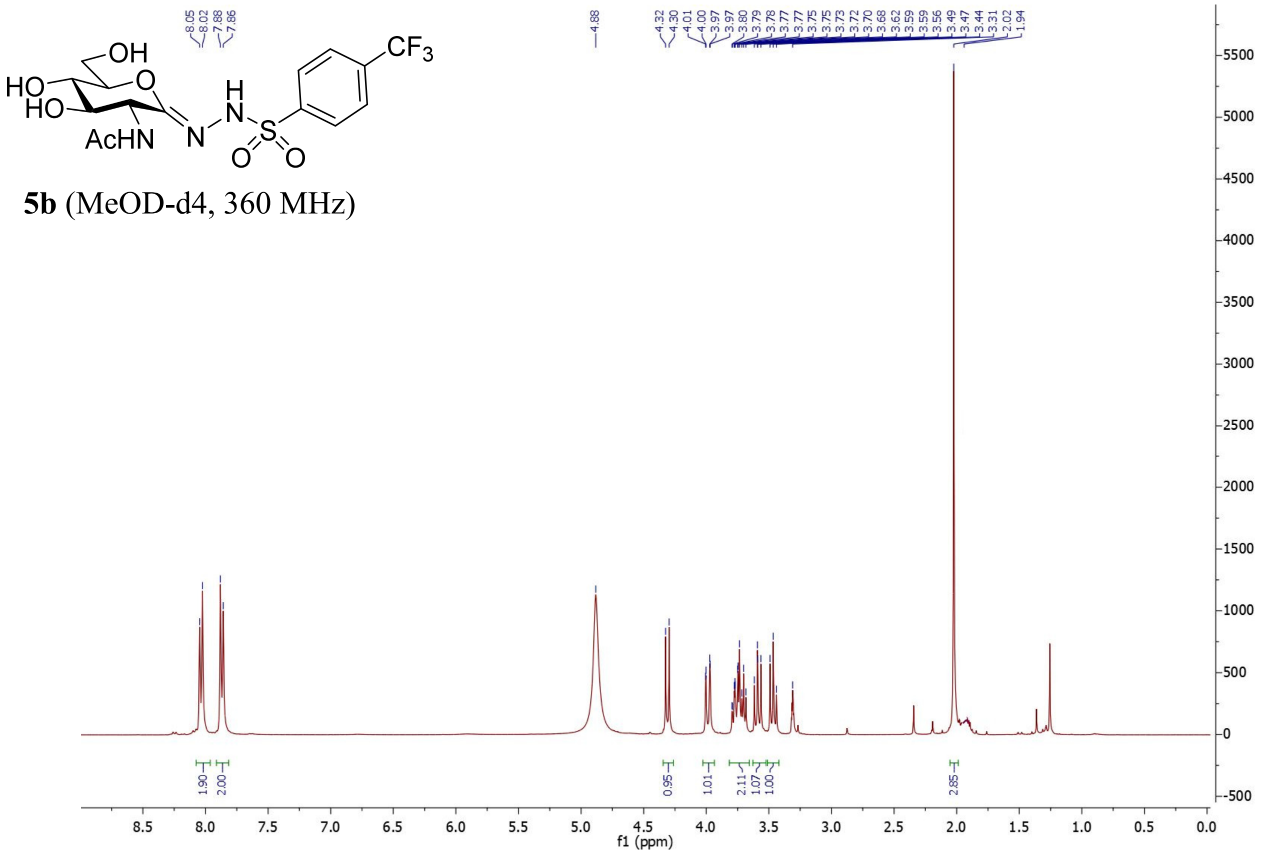

5b (MeOD-d4, 360 MHz)

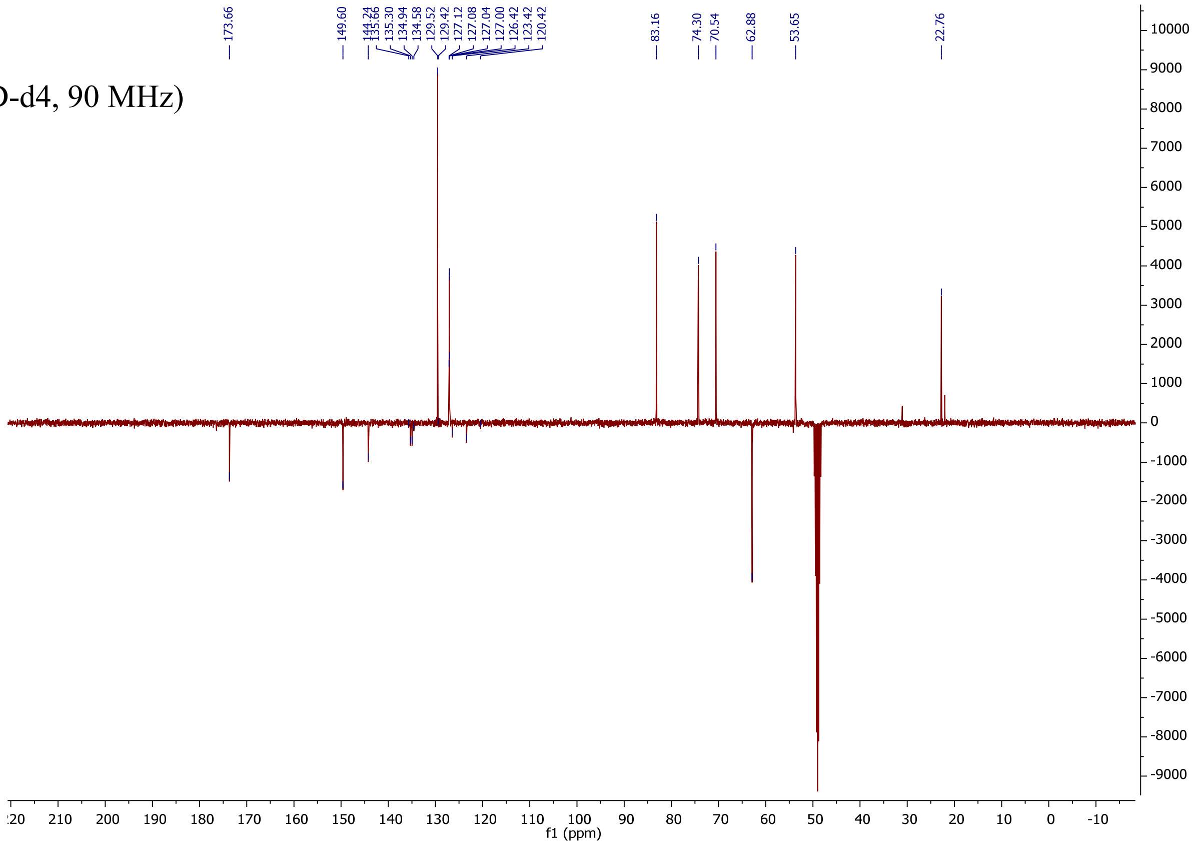

(MeOD-d4, 90 MHz)

^{1}H and ^{13}C NMR spectra of compound **5c**

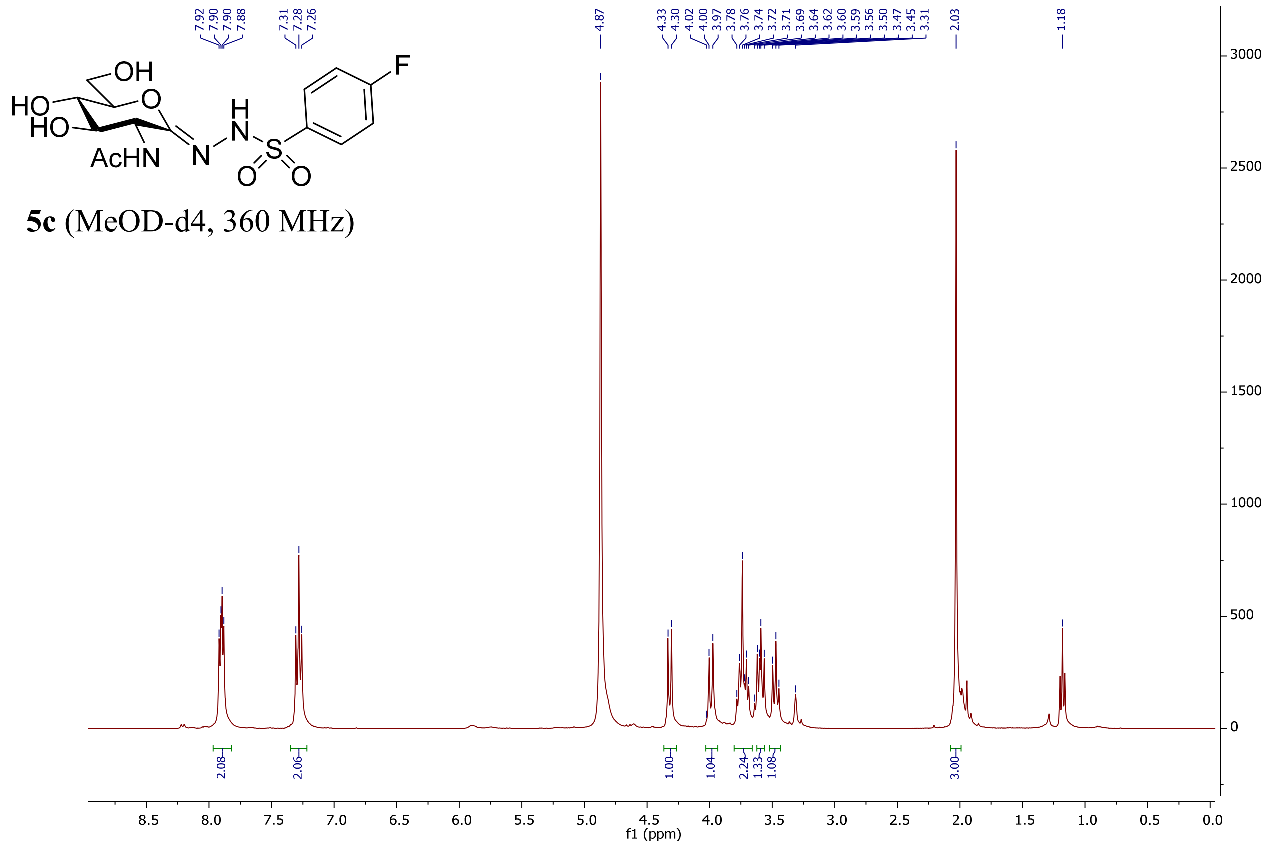

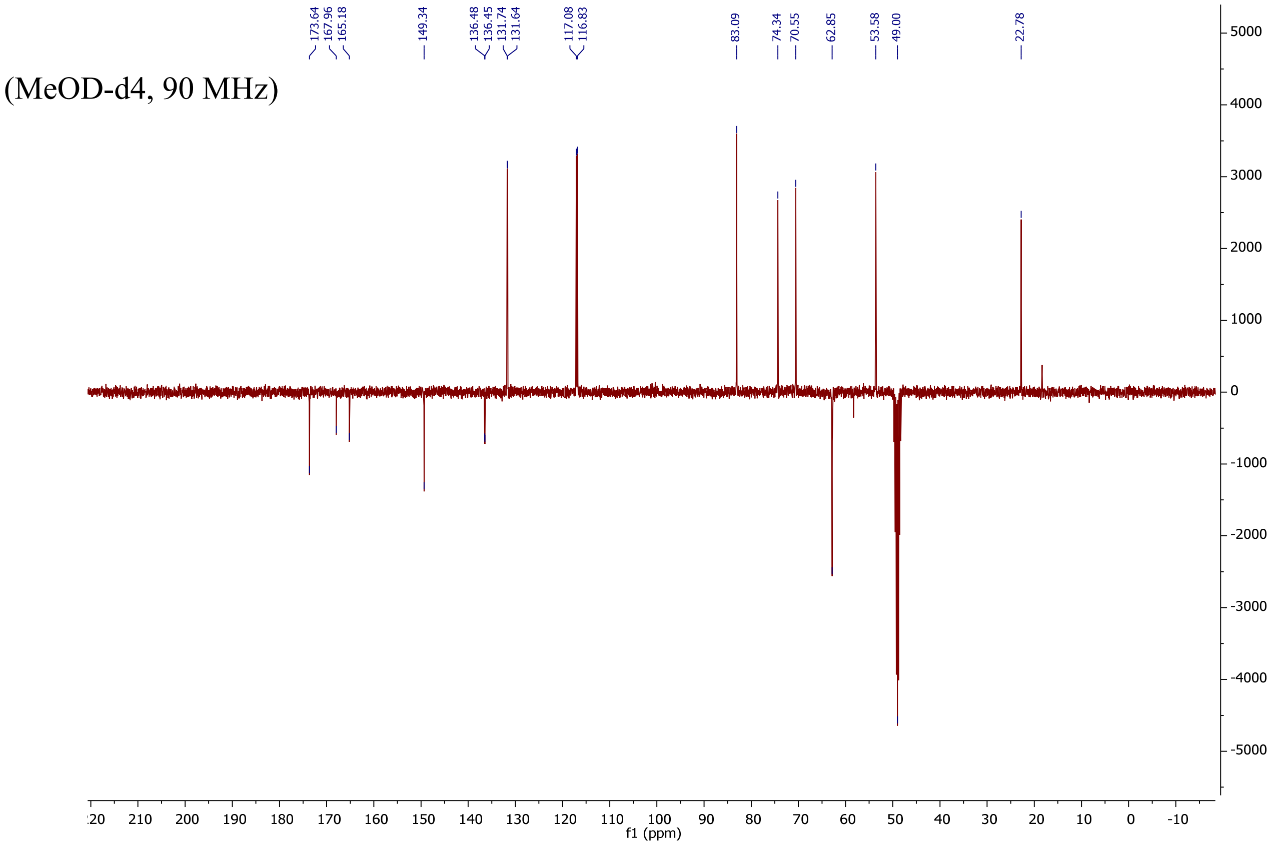

^{1}H and ^{13}C NMR spectra of compound **5d**

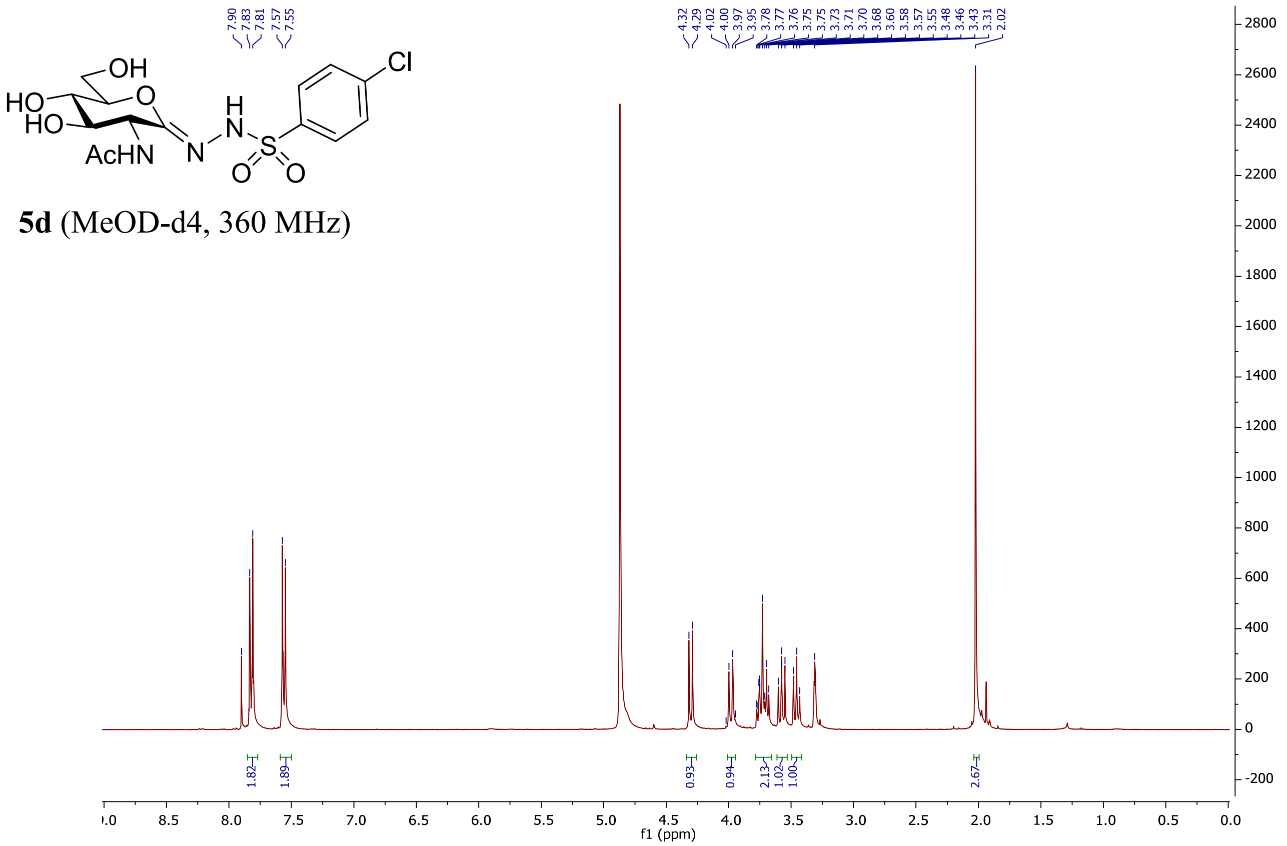

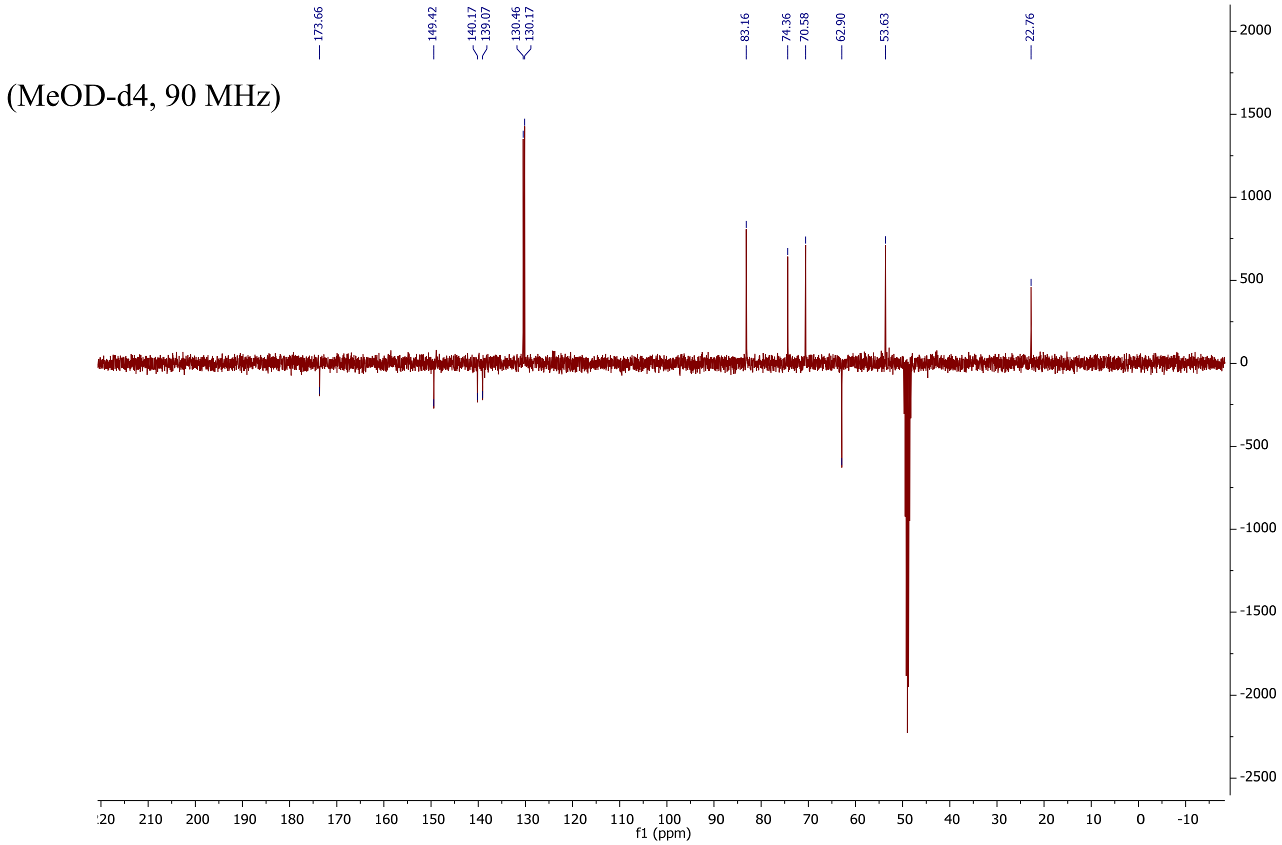

^{1}H and ^{13}C NMR spectra of compound **5e**

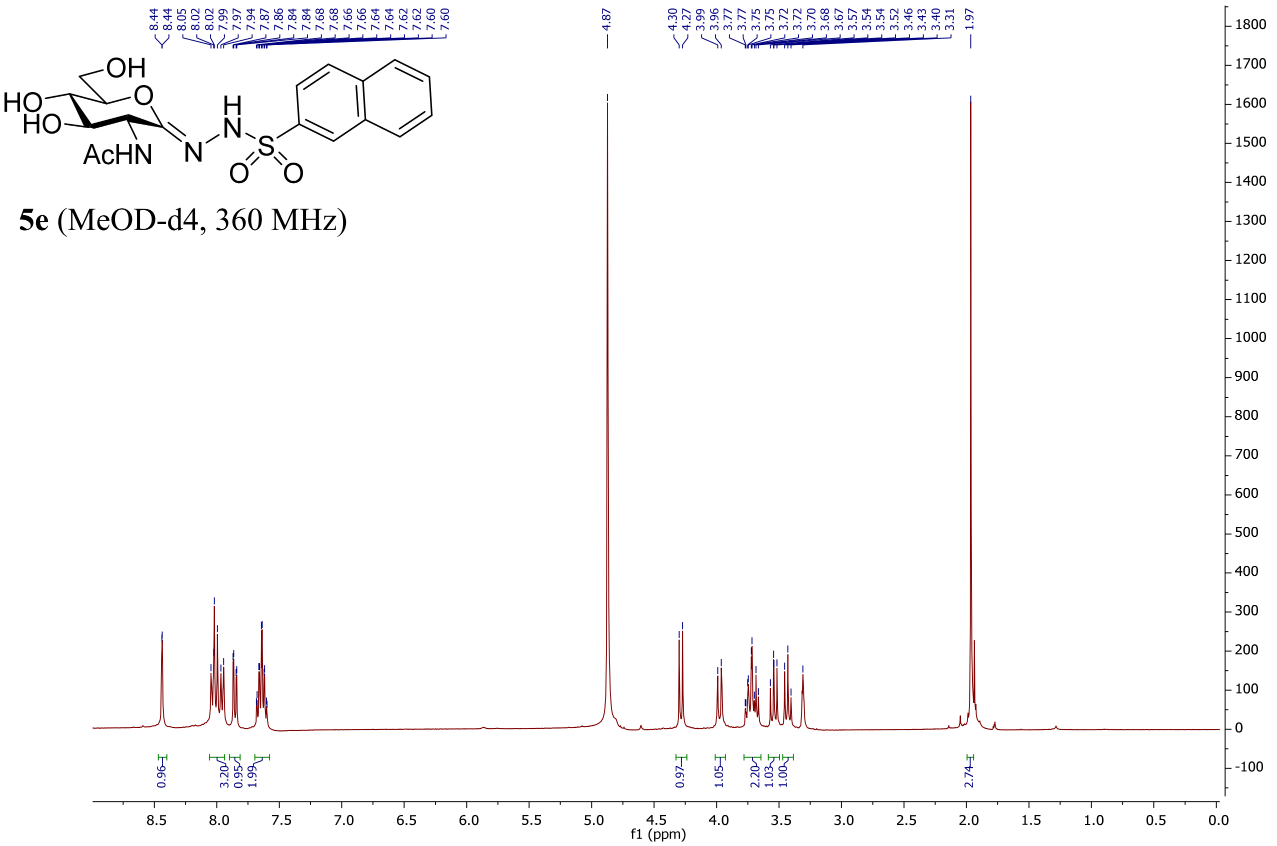

(MeOD-d4, 90 MHz)

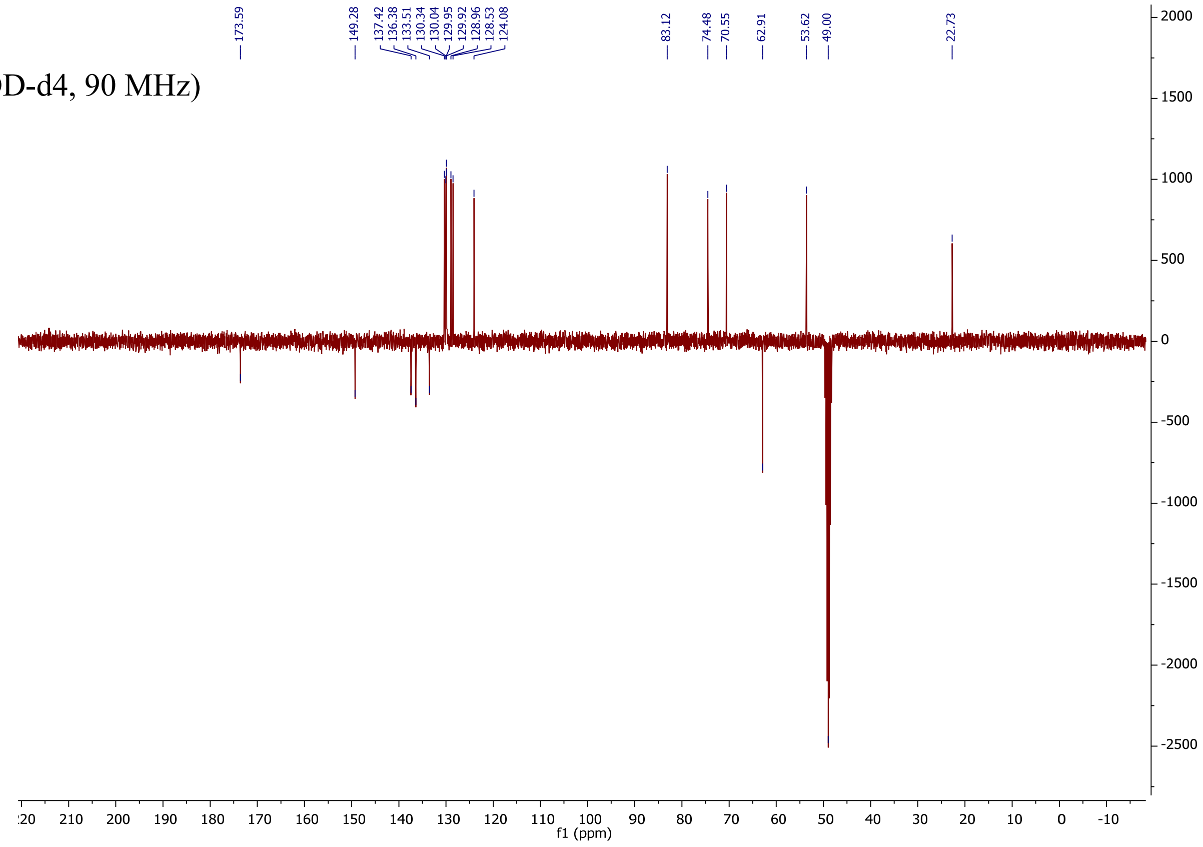

^{1}H and ^{13}C NMR spectra of compound **5f**

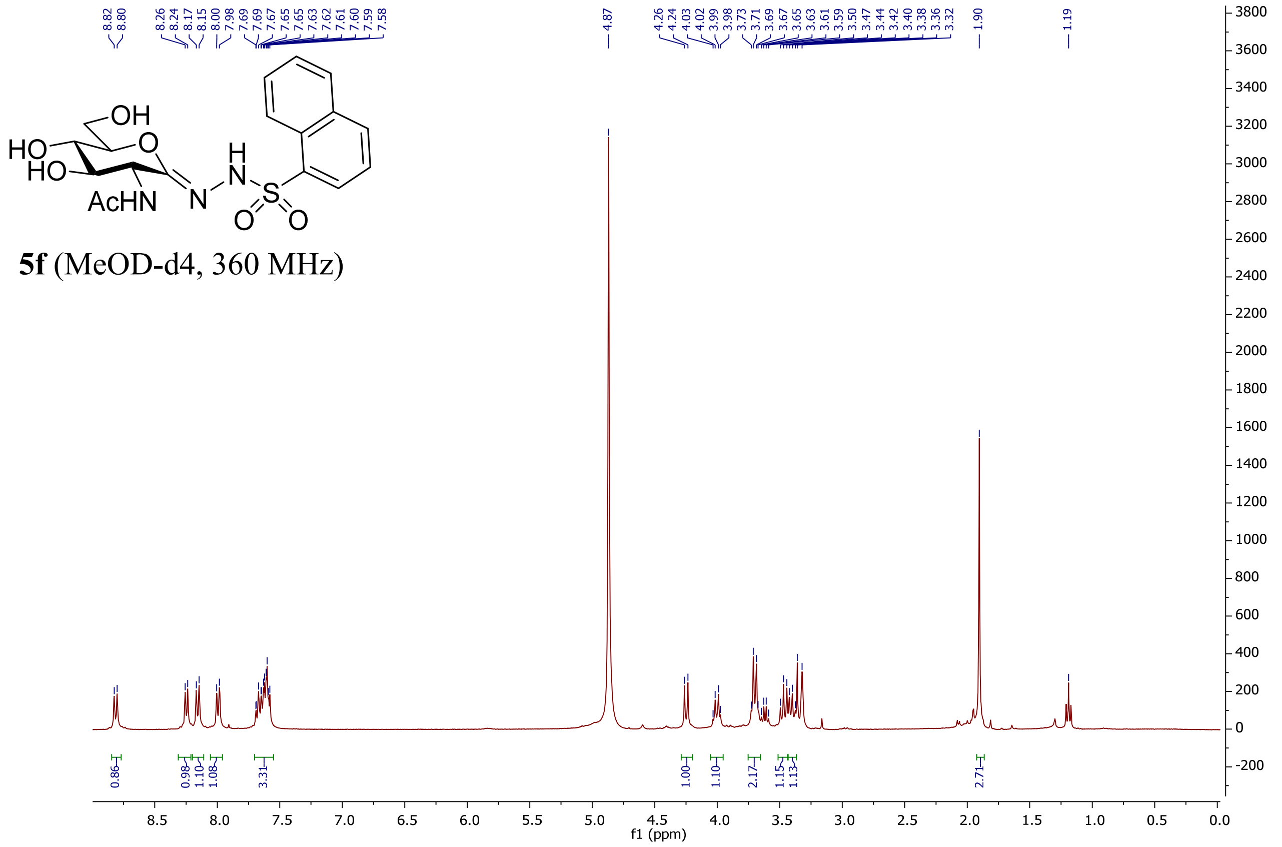

5f (MeOD-d4, 360 MHz)

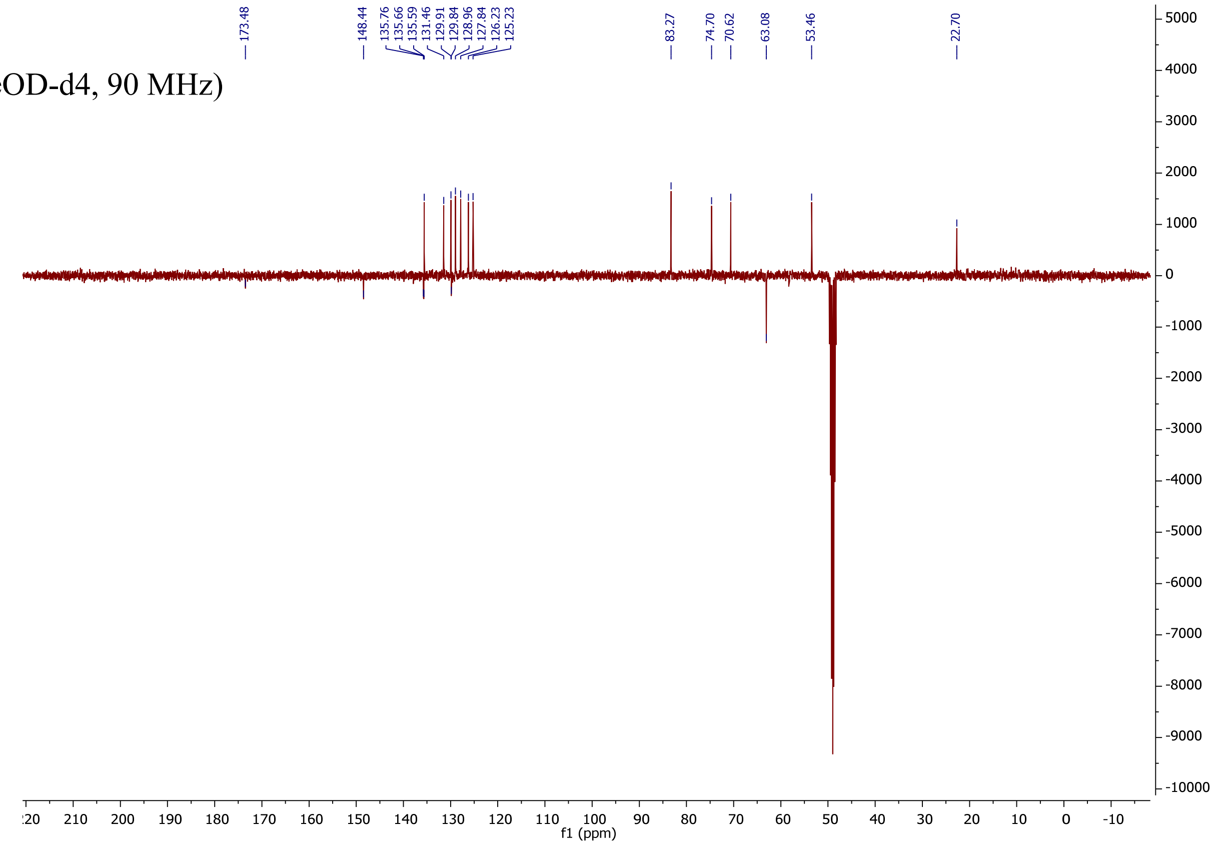

(MeOD-d4, 90 MHz)

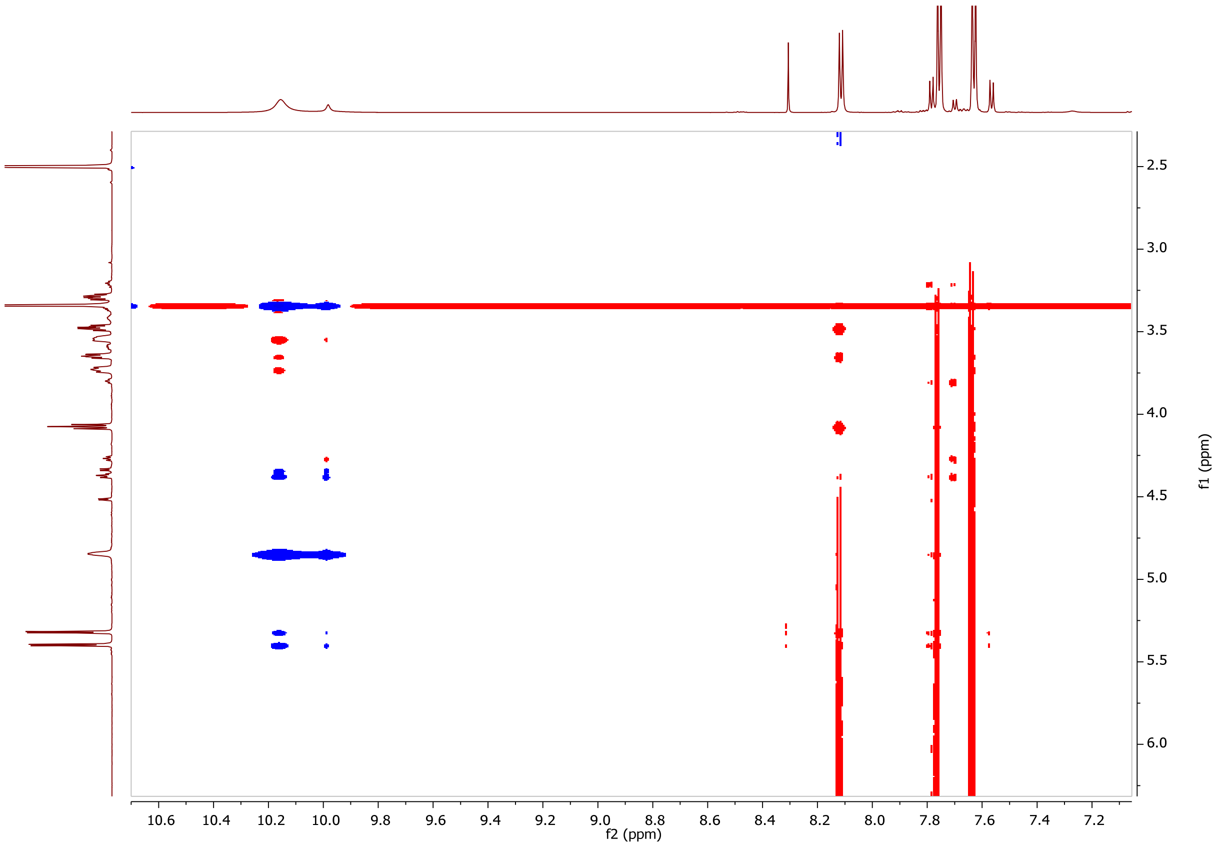

2.5
3.0
3.5
4.0
4.5
5.0
5.5
6.0
f1 (ppm)
10.6
10.4
10.2
10.0
9.8
9.6
9.4
9.2
9.0
8.8
8.6
8.4
8.2
8.0
7.8
7.6
7.4
7.2
f2 (ppm)

Dixon and Cornish-Bowden plots of hOGA inhibition data

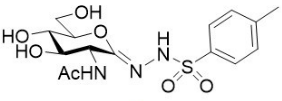

5a

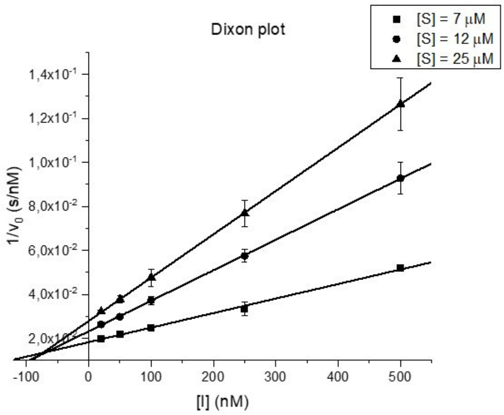

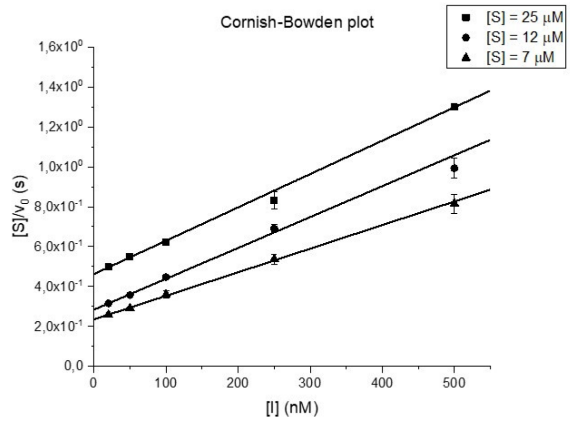

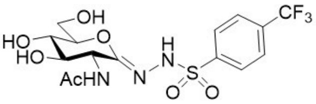

5b

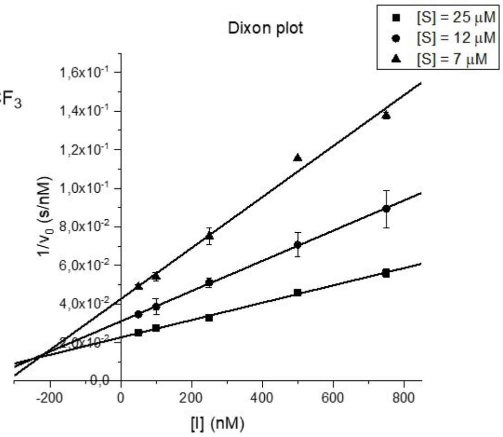

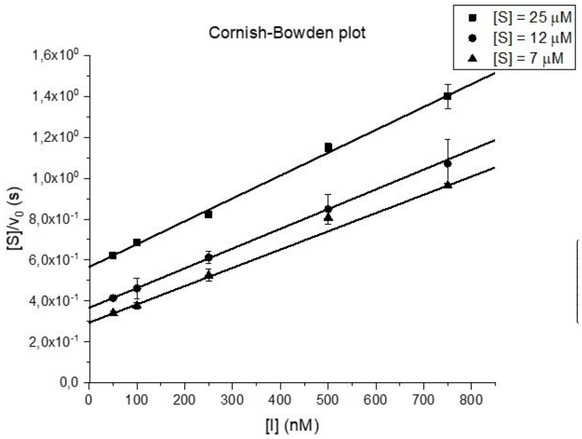

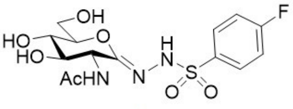

5c

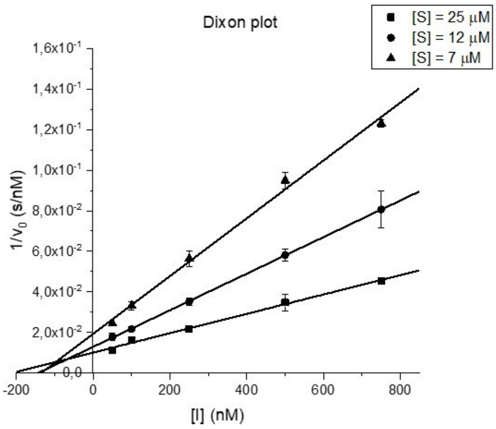

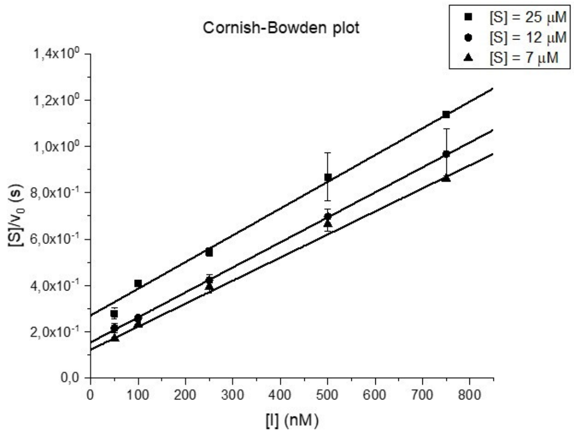

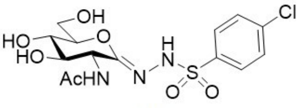

5d

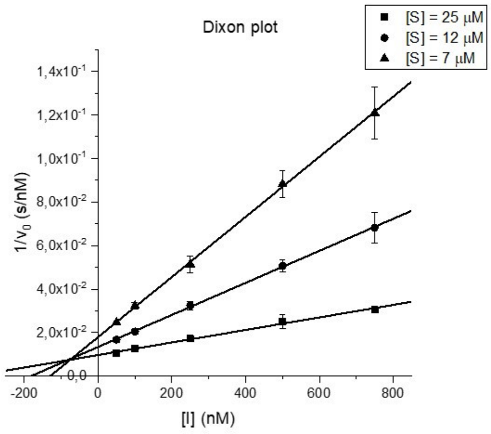

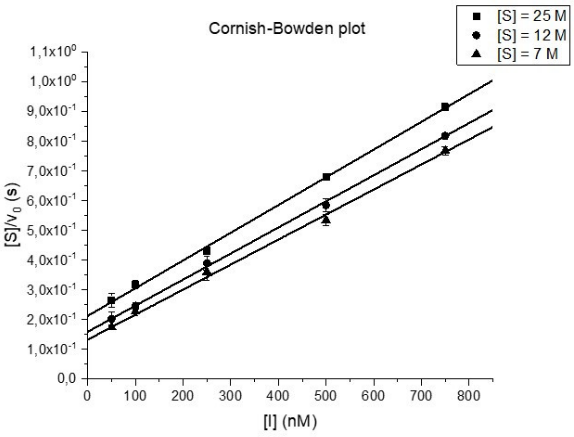

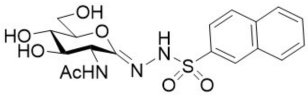

5e

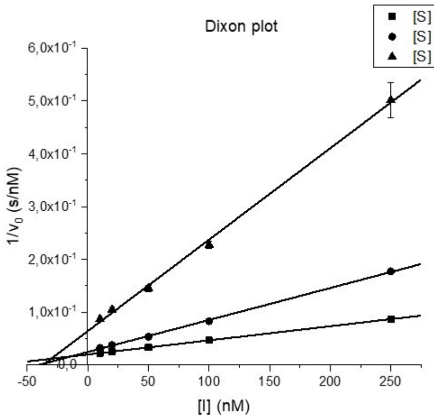
Dixon plot
[S] = 25 μM
[S] = 12 μM
[S] = 7 μM
1/v0 (s/nM)
[I] (nM)
6,0x10-1
5,0x10-1
4,0x10-1
3,0x10-1
2,0x10-1
1,0x10-1
0,0
-50
0
50
100
150
200
250

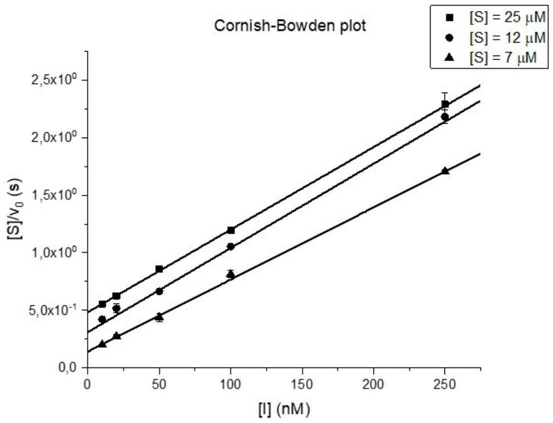
Cornish-Bowden plot
[S] = 25 μM
[S] = 12 μM
[S] = 7 μM
[S]/v0 (s)
[I] (nM)
2,5x10^0
2,0x10^0
1,5x10^0
1,0x10^0
5,0x10-1
0,0
0
50
100
150
200
250

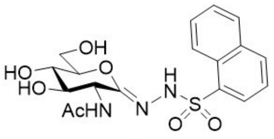

5f

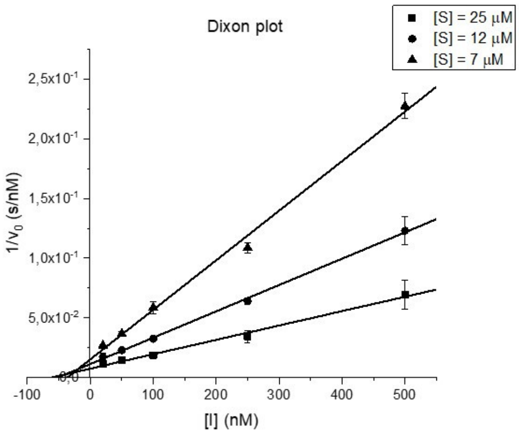
Dixon plot
[S] = 25 μM
[S] = 12 μM
[S] = 7 μM
1/v0 (s/nM)
[I] (nM)
2,5x10-1
2,0x10-1
1,5x10-1
1,0x10-1
5,0x10-2
0,0
-100
0
100
200
300
400
500

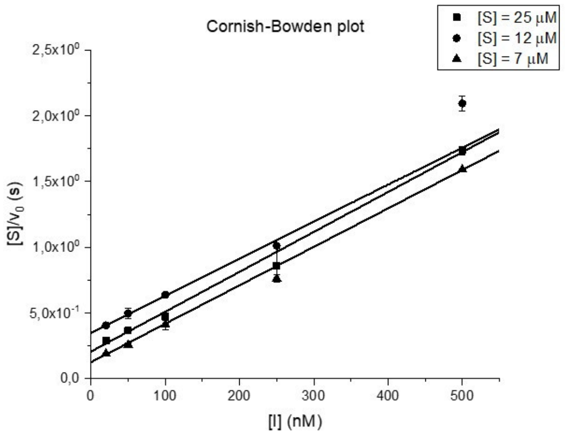
Cornish-Bowden plot
[S] = 25 μM
[S] = 12 μM
[S] = 7 μM
[S]/v0 (s)
[I] (nM)
2,5x10^0
2,0x10^0
1,5x10^0
1,0x10^0
5,0x10-1
0,0
0
100
200
300
400
500

Michaelis-Menten kinetic curves and Lineweaver-Burk plots of hHexB inhibition

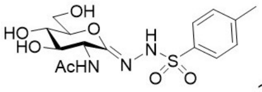

5a

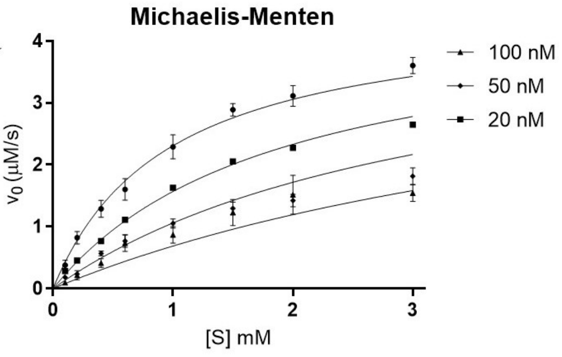

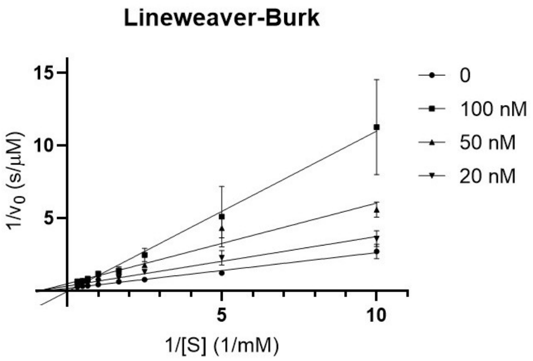

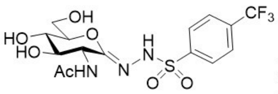

5b

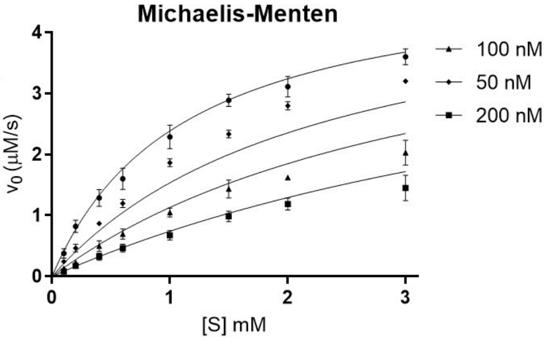

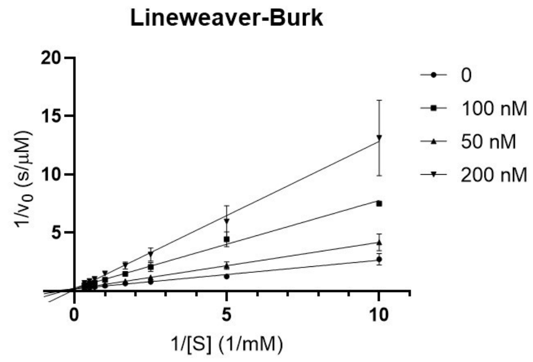

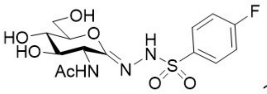

5c

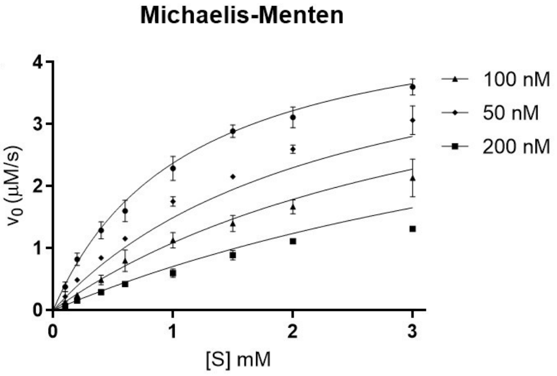

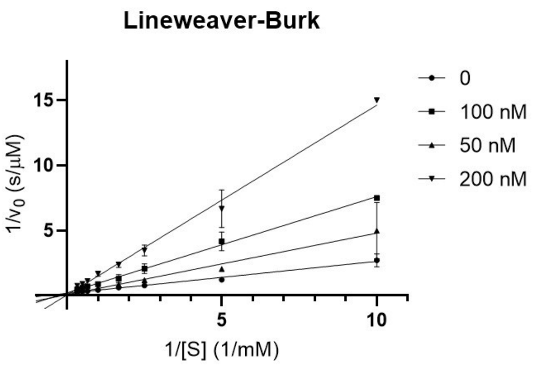

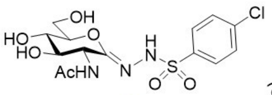

5d

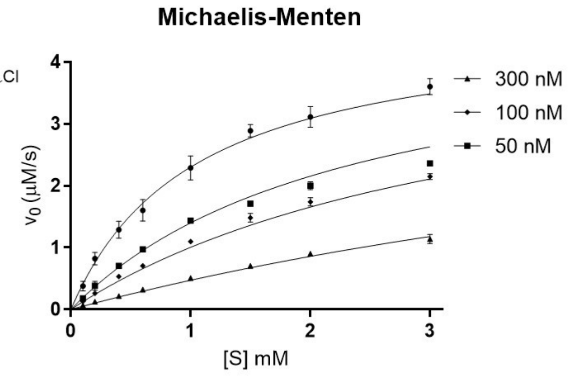

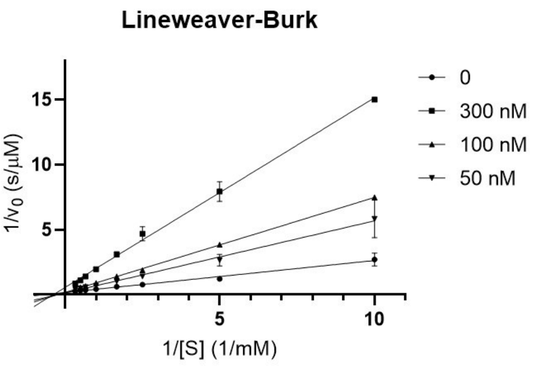

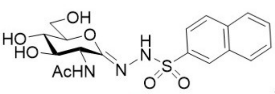

5e

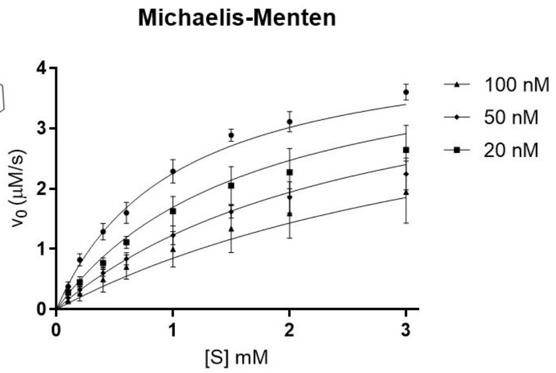

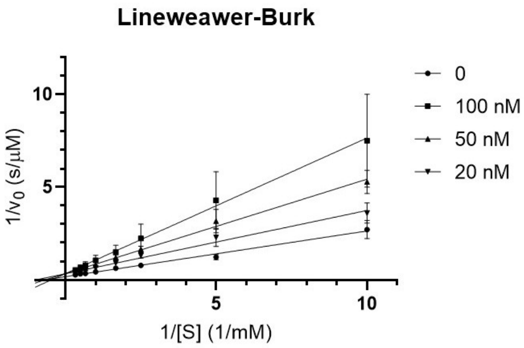

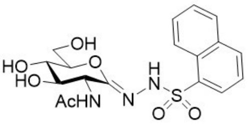

5f

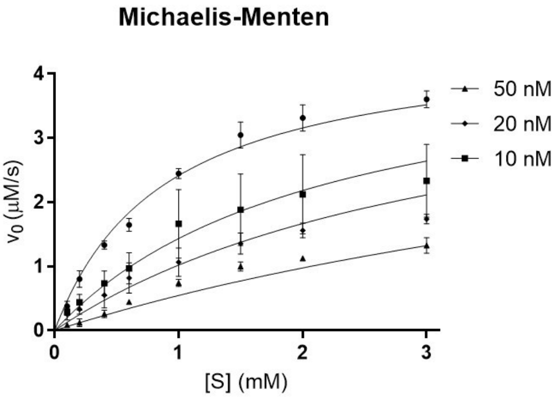

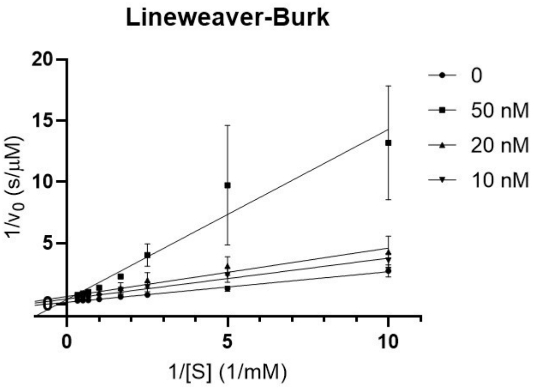

Figure S1. ^{1}H-^{1}H ROESY spectrum of compound **5d** N(2)H (10.16 ppm)
